# Hadrurid Scorpion Toxins: Evolutionary Conservation and Selective Pressures

**DOI:** 10.3390/toxins11110637

**Published:** 2019-11-01

**Authors:** Carlos E. Santibáñez-López, Matthew R. Graham, Prashant P. Sharma, Ernesto Ortiz, Lourival D. Possani

**Affiliations:** 1 Department of Biology, Eastern Connecticut State University, 83 Windham St. Willimantic, CT 06266, USAgrahamm@easternct.edu (M.R.G.); 2 Department of Integrative Biology, University of Wisconsin-Madison, 430 Lincoln Drive, Madison, WI 53706, USA; prashant.sharma@wisc.edu; 3 Departamento de Medicina Molecular y Bioprocesos, Instituto de Biotecnología, Universidad Nacional Autónoma de México, Avenida Universidad 2001, Cuernavaca, Morelos 62210, Mexico; erne@ibt.unam.mx

**Keywords:** evolutionary shifts, Hadruridae, negative selection, phylogenomics, venom transcriptome

## Abstract

Scorpion toxins are thought to have originated from ancestral housekeeping genes that underwent diversification and neofunctionalization, as a result of positive selection. Our understanding of the evolutionary origin of these peptides is hindered by the patchiness of existing taxonomic sampling. While recent studies have shown phylogenetic inertia in some scorpion toxins at higher systematic levels, evolutionary dynamics of toxins among closely related taxa remain unexplored. In this study, we used new and previously published transcriptomic resources to assess evolutionary relationships of closely related scorpions from the family Hadruridae and their toxins. In addition, we surveyed the incidence of scorpine-like peptides (SLP, a type of potassium channel toxin), which were previously known from 21 scorpion species. We demonstrate that scorpine-like peptides exhibit gene duplications. Our molecular analyses demonstrate that only eight sites of two SLP copies found in scorpions are evolving under positive selection, with more sites evolving under negative selection, in contrast to previous findings. These results show evolutionary conservation in toxin diversity at shallow taxonomic scale.

## 1. Introduction

Animal venoms are outstanding evolutionary innovations used to subdue and digest prey, with other important functions, such as self-defense or intraspecific conflicts [1,2,3,4,5]. Venoms are secretions consisting of organic and inorganic components that alter cellular processes in the target organism. Several arthropod groups are well known for their venoms, such as bees and wasps [6,7], centipedes [8,9,10], remipedes [11], and arachnids (e.g., see [12,13]). Some of the most efficient venoms occur in scorpions, which possess venom cocktails rich in peptides that affect ion channels [14,15,16]. Toxins are thought to have arisen from diversification of paralogs of housekeeping genes, followed by neofunctionalization driven by positive selection in coding regions [15,17,18,19,20].

Our knowledge of scorpion toxins has benefitted from high-throughput sequencing technologies, though taxonomically, sampling favors the diverse scorpion family Buthidae. In recent years, however, transcriptomic analyses have expanded beyond the Buthidae to the other 19 scorpion families (e.g., see [21,22,23,24,25,26]). These efforts have demonstrated remarkable phylogenetic inertia in scorpion venom components at higher-level categories, but little is known about how venom composition and toxin evolution vary among species below the family level.

The North American scorpion family Hadruridae is represented by nine species in two genera, including several of the largest species worldwide [27]. Of these, the diversity and effects of venom components from *Hoffmannihadrurus gertschi* have been extensively studied [28,29,30]. More recently, a transcriptome analysis, conducted on the venom gland of *Hadrurus spadix*, was published [31]. Though both species showed similar venom composition, a comparative analysis can shed light on the evolution of toxin components among these and other closely related species.

In this study, we focused on five hadrurid species: *Hadrurus arizonensis*, *Hadrurus spadix*, *Hadrurus concolorous*, *Hoffmannihadrurus aztecus*, and *Hoffmannihadrurus gertschi*. We used RNA-Seq to sequence de novo the venom gland transcriptome of *Hoffmannihadrurus aztecus* and *Hadrurus concolorous*.

We assessed the commonality of venom composition by comparing the number of putative transcripts found in the five species. Specifically, we focused on the evolutionary history of scorpine-like peptides (SLP, sensu [22]). Originally, SLPs were called ‘orphan peptides’ due to the presence of two domains with dual functionality. The N-terminal region has cytolytic activity, whereas the C-terminus blocks potassium channel activity [32,33,34]. SLPs were first isolated from the venom of *Pandinus imperator* [35]. They have now been described in one or two gene copies, from 21 scorpion species, including nine buthids and 12 species from 10 other, non-buthid families [22]. Intriguingly, buthid SLPs (with the exception of one sequence) and non-buthid SLPs form two mutually monophyletic clusters that are phylogenetically restricted to the parvorders Buthida and Iurida, respectively [22], suggesting that the origin of SLPs could predate the diversification of scorpions. To assess SLP evolution in the context of comprehensive hadrurid phylogeny, we inferred internal hadrurid relationships and branch lengths using phylogenomic datasets, and surveyed venom gland transcriptomes to discover and map the distribution of non-buthid SLP homologs. We then inferred the direction of selection acting on the codon sequences of non-buthid SLP genes and evaluated the evolutionary dynamics of paralogs of SLPs within the Hadruridae.

## 2. Results

### 2.1. Hadrurid Phylogenomic Tree and Divergence Time Estimate

**Figure 1:**
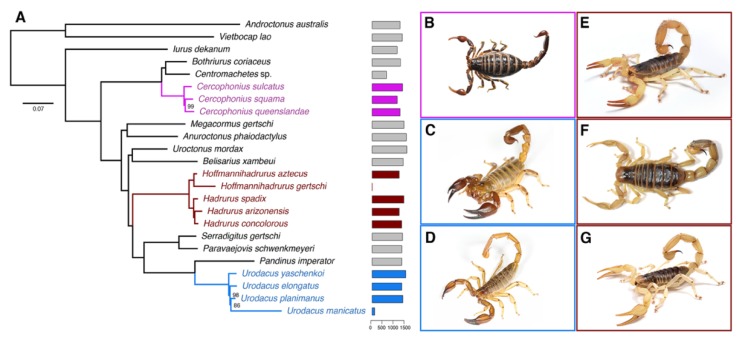
(**A**) Maximum likelihood tree topology recovered from the analysis of 1982 genes, with 24 scorpion species. Bars to the right of termini indicate numbers of orthologs. Numbers on nodes indicate ultrafast bootstrap support values under 100. (**B**–**G**) Representative species of scorpions studied here: (**B**) *Cercophonius squama* (Gervais, 1843); (**C**) *Urodacus yaschenkoi* (Birula, 1903); (**D**) *Urodacus elongatus* Koch, 1977; (**E**) *Hadrurus spadix* Stahnke, 1940; (**F**) *Hadrurus concolorous* Stahnke, 1969; (**G**) *Hadrurus arizonensis* Ewing, 1928. Photos by Nick Volpe (**C**,**D**), Matthew Graham (**B**,**E**,**G**) and Carlos Santibañez (**F**).

Maximum likelihood (ML) analysis and species tree reconciliation of a 1982-locus matrix (727,571 amino acid sites, 35.6% missing data) yielded a topology that was largely congruent with phylogenomic trees previously published by us [27,36]. The monophyly of the two basal clades Buthidae and Iuridae, along with relationships of all superfamilies within the Iuridae, were recovered with 100% nodal support (Figure 1 and Figure S1). Monophyly of the Hadrurinae, *Hadrurus*, and *Hoffmannihadrurus* were all strongly supported (Figure 1). *Cercophonius* and *Urodacus* (two distantly related lineages with sampling of multiple congeners and patristic distances comparable to those within *Hadrurus*) were both monophyletic with strong support (Figure 1). Estimates of the time to the most recent common ancestor (tMRCA) were 7–11 Myr for the Hadrurinae, 8–34 Myr for the Urodacidae, and 86–196 Myr for the Bothriuridae (Figures S2 and S3), in agreement with previous hypotheses [37].

### 2.2. The Repertoire of Venom-Specific Transcripts in the H. concolorous and H. aztecus Libraries

**Figure 2:**
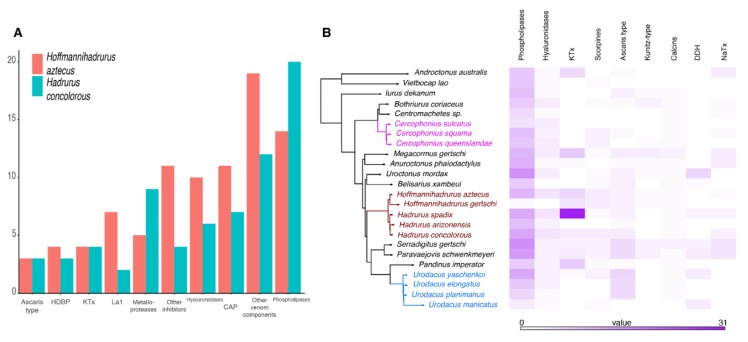
Comparative transcriptomic analyses. (**A**) Distribution of the annotated transcripts from the venom gland transcriptomes of *Hadrurus concolorous* and *Hoffmannihadrurus aztecus*, according to protein families. (**B**) Comparison of selected venom protein families in the libraries of the scorpion species studied herein.

After sequencing, quality assessment, and rRNA and adapter removal, 92,004,852 reads were assembled into 122,574 putative transcripts from *H. concolorous* (N50:1467 bp) and 44,355,818 reads were assembled into 83,643 putative transcripts from *H. aztecus* (N50:709 bp). Of these, 22,189 *H. concolorous* sequences and 28,101 *H. aztecus* sequences were identified, matching gene sequences in databases. We recorded 74 putative coding transcripts for *H. concolorous* and 96 for *H. aztecus*, representing the following venom components: potassium-channel toxins (KTx), sodium-channel toxins (NaTx), enzymes, protease inhibitors, La1-like peptides, host-defense peptides (HDPs), members of the cysteine-rich secretory protein, antigen 5, and pathogenesis-related 1 protein (CAP) superfamily, scorpine-like peptides (SLP), and other venom components (Figure 2A, Tables S1 and S2). Comparative venom transcriptomic analysis showed similar numbers of putative transcripts in the venom repertoires of *H. gertschi* and *H. spadix* (except for KTx), and among *Urodacus* and *Cercophonius* species (Figure 2B).

### 2.3. Molecular Evolution of Scorpine-Like Peptides (SLP)

**Figure 3:**
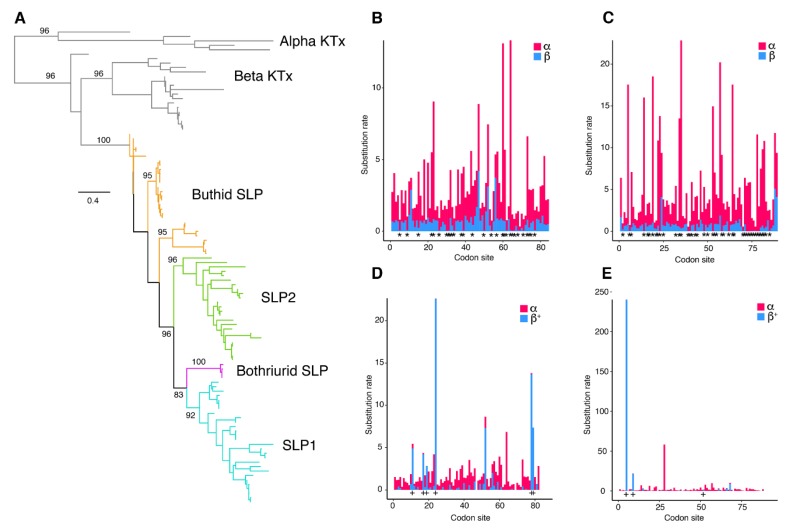
Evolutionary analyses of scorpine-like peptides. (**A**). Maximum likelihood (ML) gene tree topology of scorpine-like peptides (SLPs) and related toxins (αKTx and other βKTx). Ultrafast bootstrap values are shown above selected basal nodes (>75%). (**B**,**C**) Site selection analyses of SLP1 (**B**) and SLP2 (**C**) sequences with FUBAR. Visualization of synonymous substitution rates (α, red) and non-synonymous substitution rates (β, blue) for sites exhibiting positive/neutral evolution. Asterisks indicate sites with α greater than β, *p* > 0.95. (**D**,**E**) Site selection analyses of SLP1 (**D**) and SLP2 (**E**) sequences with MEME. + signs indicate sites with β^+^ values greater than α, *p* > 0.95.

Eighty-four scorpine-like sequences were retrieved from the libraries generated herein, with others from GenBank, UniProt, and previous studies. Maximum Likelihood (ML) and Bayesian Inference (BI) gene trees recovered SLPs as a monophyletic cluster with strong support (Figure 3A). Among SLPs, three clades including only buthid sequences and three clades including mainly non-buthid sequences were recovered, in agreement with previous hypotheses (in [22]; Figure 3A and Figure S4). We observed a distal duplication of SLP (SLP1 and SLP2, Figure 3 and Figure S4) that is retained by a subset of iurid scorpions (the clade excluding the Iuridae and Bothriuridae). Our results showed that *H. concolorous* and *H. aztecus* have three putative SLPs, whereas *H. gertschi* has two and *H. spadix* one (as opposed to four, as reported by [31], missing in our assembly).

Evidence of positively selected sites in these peptides was assessed using MEME and FUBAR. The MEME results suggested that four sites have experienced episodic positive selection (*p* < 0.05; Figure S5). In contrast, the FUBAR analysis identified episodic negative selection at 44 sites, but none evolving under positive selection (*p* < 0.05; Figure S5).

**Figure 4:**
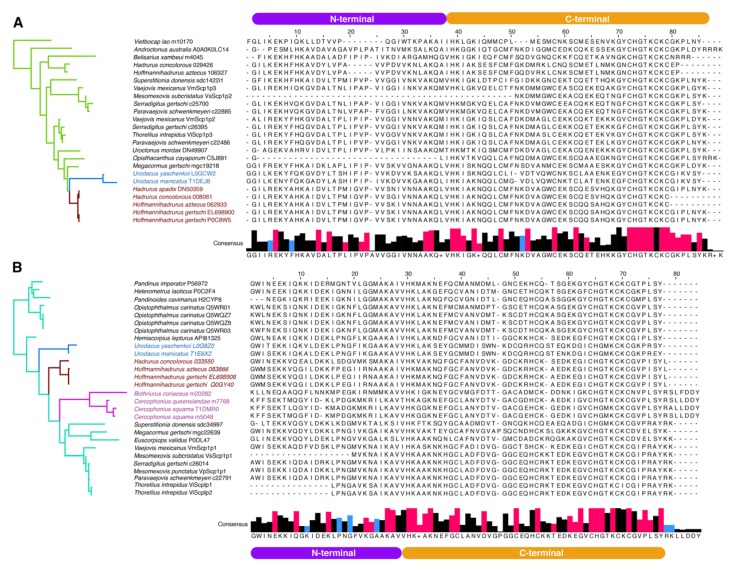
Multiple sequence alignment (MSA) of the mature peptide of SLP2 (**A**) and SLP1 (**B**) in order of appearance in the phylogeny (left, color coded as in previous figure). Consensus sequence histograms of each clade below the MSA (+ sign indicates a highly variable site). In red, sites evolving under negative selection, as detected with FUBAR, and in blue, sites evolving under positive selection as detected with MEME (see Figure 3).

To analyze evolution of scorpine paralogs, MEME and FUBAR analyses were conducted on each gene copy (SLP1 and SLP2). FUBAR analysis detected 30 sites evolving under negative selection in both copies. Three sites in SLP1 and six sites in SLP2 were considered to be evolving under positive selection by MEME analysis (Figure 3C–E). Multiple sequence alignments (MSA) of each SLP (SLP1 and 2) showed consistency among hadrurid toxins (Figure 4A,B). For downstream analyses, we selected only pairs of copies that were recovered as monophyletic with SLPs described previously for *H. gertschi* (Hge-scorpine 1-2).

**Figure 5:**
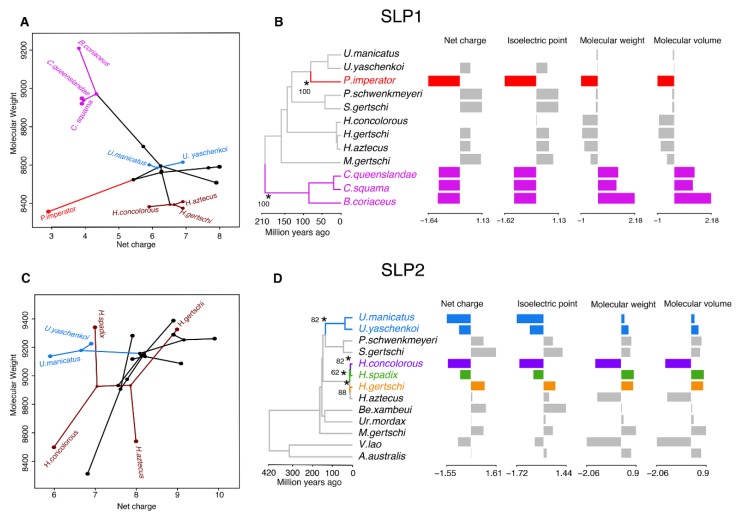
Evolutionary analyses of the chemical properties of SLP homologs. (**A**) Visualization of the phylogenomic tree on the morphospace of the molecular weight and net charge of SLP1 (**A**) and SLP2 (**C**). Hadrurids, urodacids, and *Cercophonius* are colored accordingly to phylogenomic topology. (**B**–**D**) Two (SLP1) and four (SLP2) evolutionary shifts in the optimum chemical properties of SLPs under an Ornstein–Uhlenbeck (OU) process. Edges with a major shift are annotated with asterisk and bootstrap support values.

The effect of molecular weight, molecular volume, net charge, and isoelectric point were evaluated for both copies of SLPs (combined and independently) using a principal component analysis (*prcomp* in R). Combined PCAs of both SLPs showed that 99.63% of the variation was explained by PC1, which segregated the SLP1 from SLP2 by molecular weight and volume, whereas PC2 (0.67%) split them by net charge (Figure S6). Three SLP2s copies were grouped with most of the SLP1s, due to their low molecular weight and volume. Similarly, almost 100% of the variation (99.72%) was explained by PC1 in both independent analyses. By molecular weight and volume, it segregated SLP2s of *Vietbocap lao*, *H. aztecus*, and *H. concolorous* from the rest of the sequences, and the SLP1 from *P. imperator* from other SLP1 sequences. In this analysis, PC2 also separated the sequences by net charge in both analyses. Superimposition of the phylogram onto the principal component space of SLP1 and SLP2 showed that SLP1 more strongly retains a phylogenetic signal (Figure 5A,C). We tested the correlation between these biochemical properties and found that molecular weight and volume are strongly correlated (*p* < 0.05; *r^2^* = 1.00), as are the net charge and the isoelectric point (*p* < 0.05, *r^2^* = 0.92; Figure S6).

We then inferred changes in evolutionary dynamics of the SLP gene family using 84 sequences. Two evolutionary shifts were detected in SLP1 with bootstrap support (BS) above 50% (Figure 5B). These occurred in the branch subtending *P. imperator* and the branch subtending the Bothriuridae, both with BS = 100%. In contrast, four major shifts were detected with BS > 50% across the evolutionary history of SLP2 (Figure 5D). These subtended *Uroctonus* (BS = 82%), *H. concolorous* (BS = 82%), *H. aztecus* (BS = 88%), and *H. spadix* (BS = 62%). However, when only two variables were considered (molecular weight and net charge), no major evolutionary shifts were detected in the gene tree of SLP2, with the same two shifts detected in evolution of SLP1.

## 3. Discussion

The study of the early evolutionary origins of scorpion toxins has been complicated by issues of homology inference and taxonomic sampling. Recently, the use of transcriptomics to resolve the scorpion tree of life has yielded sequence data that can be used to assess venom composition concomitantly with reconstruction of phylogenetic relationships. Our analyses, based on nearly 2000 loci, provide the first resolution of relationships within the scorpion family Hadruridae. Most notably, we provide evidence of the conservation of venom composition up to the family level. Furthermore, our results detected different evolutionary selective pressures on the two domains of scorpine-like peptides. These results strongly contrast with previous hypotheses that scorpion cystine-stabilized alpha/beta fold toxins (CSαβ) peptides are evolving under positive selection to increase potency. Instead, more sites are evolving under negative selection, suggesting an evolutionary conservation in function for these peptides since the estimated Permian diversification of scorpions [37], continuing through the estimated Neogene period of diversification of the Hadruridae. This study provides parameters to test the significance of toxic evolutionary dynamics in the extraordinary diversification of this culturally iconic group of arachnids.

Maximum likelihood and species tree analyses supported the monophyly of the family Hadruridae and its two constituent genera. Our results are congruent with those presented in a more sparsely sampled phylogenomic study [27]. The molecular phylogeny presented here demonstrates the monophyly of *Hoffmannihadrurus* as a lineage distinct from *Hadrurus*, in agreement with previous hypotheses based on morphology [38,39]. Our results suggest that the tradeoff between missing data and number of genes does not adversely impact reconstruction of the major scorpion clades (compare results in [27,36,37,40]). Molecular dating supported the divergence of the Hadruridae 7–11 Mya, in partial agreement with formation of the Trans-Mexican Volcanic Belt, which could have been a vicariance event that isolated *Hoffmannihadrurus* in the south from *Hadrurus* in the north [41,42,43].

Of marked interest to us in the transcriptomic analyses of the Hadruridae were the evolutionary dynamics of, and selective pressures on, scorpine-like peptides, which exhibit paralogous copies in a subset of hadrurid species. Buthid scorpine-like peptides differ from non-buthid SLPs with respect to the size of the mature peptide (<75 sites in buthids vs. >80 sites in non-buthids). Gene tree topologies, however, do not reflect a phylogenetic signal due to additional duplications in two hadrurid species (*H. concolorous* and *H. aztecus*) or other scorpions (e.g., in vaejovids). Only SLP2 was recovered from buthid scorpions. 

Comparative transcriptomic analyses of hadrurid species highlights the evolutionary process that contributes to the diversity of CSαβ scorpion toxins. Results suggest that the SLP gene underwent duplication, which is concordant with the traditional model of venom toxin gene evolution [17,18,19,20]. However, evolutionary analyses of scorpine-like peptides showed that few non-cysteine amino acid substitutions occurred between the cysteines of the C-terminus, including insertion/deletions. Consequently, our results do not support a previous hypothesis [15] that CSαβ toxins have evolved under the influence of positive selection. Fewer than five sites in each SLP copy have experienced diversifying selection. Moreover, the majority of these sites were found at the N-terminus (a putative alpha helix), suggesting that positive selection might play an important role in evolution of this domain, but not at the C-terminus (Figure 4). Thus, this highly conserved conformation of both SLP copies, along with the incidence of several sites evolving under negative selection to reduce alternate states, is consistent with the tendency to preserve function (e.g., venom potency or target specificity [19,36,44]).

Parametric analyses of the predicted peptides also suggest that both SLP copies possess similar biochemical properties. For example, SLP1 homologs have shorter sequences (<84 amino acids) with lower molecular weights and volumes (with two exceptions) and more negative net charges (with three exceptions, *p* < 0.01). In contrast, SLP2 homologs have longer sequences (>84 amino acids) with correspondingly greater molecular weights and volumes (with three exceptions) and more positive net charges (with three exceptions; *p* < 0.01). To test the evolutionary dynamics of these protein traits, we assessed the role of selection during hadrurid evolution by modeling shifts in SLP biochemical traits under an Ornstein–Uhlenbeck (OU) process. Several studies have used this process to model evolution of continuous traits by detecting shifts to different regimes with different adaptive optima, along a time calibrated phylogeny (e.g., in [45,46,47], with a recent application to 3D structures of toxins [32]). The incidence of shifts in hadrurid SLP2s may be biased by sequence lengths from *H. aztecus* and *H. concolorous* (smaller than other SLP2) and the divergence time of these species (between 7 and 11 Myr). In contrast, the incidence of shifts in *Cercophonius* and *Bothriurus* suggests a unique evolutionary regime in the family Bothriuridae.

## 4. Conclusions

Reconstructing scorpion venom evolution is challenging due to the diversity of scorpions, the diversity of venom components, and the relative paucity of genome-scale sampling for various lineages. Application of RNA-Seq in the past decade has transformed both our understanding of scorpion phylogeny and the potential for gene discovery in venom gland transcriptomes. However, while transcriptomics provides distinct advantages, like scalability and rapidly deployable analytical toolkits, care must be taken to validate predictions of transcriptomic approaches using genomic, proteomic, and functional datasets (e.g., see [48,49]). In the absence of high-quality genomes for all surveyed species, conclusions on the number of toxin paralogs should be treated cautiously, as gene absence cannot be equated with gene loss. Similarly, proteomic and functional data are needed to bridge the gap between bioinformatic predictions and validation of venom components. Future efforts should therefore use transcriptomics as a guide for selection of high-priority targets (either specific peptides or in poorly studied species) for assessments using proteomes or functional experiments.

## 5. Materials and Methods

### 5.1. Specimen Collection and RNA Sequencing

Three specimens of *Hadrurus concolorous* were collected at night with the aid of ultraviolet lamps at Guerrero Negro in Baja California Sur (27°59′1.81”N, 114°1′5.12”W) on August 9, 2015 (Col. CESL and G. Contreras-Félix). By the same means, two specimens of *Hoffmannihadrurus aztecus* were collected from Zapotitlán de Salinas in Puebla (18°19′30.68”N, 97°28′37.09”W) on March 9, 2016 (Col. CESL and R. Paredes). One female specimen from each species was selected for RNAseq, while the others were kept under laboratory conditions for venom collection. Telsons were dissected and placed directly into RNA denaturing solution. An SV Total RNA Isolation System (Promega, Madison, WI, USA) was used for Total RNA extraction and purification. RNA quality was assessed using a Bioanalyzer 2100 (Agilent, Santa Clara, CA, USA). Samples lacked a 28S rRNA peak, as previously reported [21], but there was no indication of RNA degradation. Paired-end cDNA libraries were prepared with an Illumina TruSeq Stranded mRNA Sample Preparation Kit (Illumina, Inc., San Diego, CA, USA). A Genome Analyzer IIx (Illumina, Inc., San Diego, CA, USA) at the Massive DNA Sequencing Facility in the Institute of Biotechnology (Cuernavaca, Mexico) was employed, with a 72-bp paired-end sequencing scheme over 200–400-bp cDNA fragments. Raw sequences were deposited in the ENA SRA database under project number PRJEB34464. After sequence cleaning and adapter clipping, reads were assembled de novo into contigs using Trinity (v. 2.8.5, https://trinityrnaseq.github.io [50]) as reported before [51], and annotated with Trinotate (v3.1.1, https://trinotate.github.io, [52]). Transcriptomic data from all other species in this study were retrieved from GenBank, with most of them (19/22) previously sequenced by our team. Protein coding regions within these assemblies were identified and predicted using TransDecoder v. 5.3.0 [50].

### 5.2. Orthology Inference and Phylogenetic Methods

Our phylogenomic dataset consisted of 24 scorpion taxa (five ingroup species, 19 outgroups; Table S3), and our methodology followed recent scorpion phylogenomic studies (i.e., in [53]). Briefly, transcriptomes were combined, and a de novo homology search was conducted using the informed orthology criterion implemented in UPhO v1.0 [54]. Libraries of four representative species, *Cercophonius sulcatus*, *Hoffmannihadrurus aztecus*, *Serradigitus gertschi*, and *Urodacus yaschenkoi*, were combined and used as queries against the database containing all species using Mmseqs2 [55]. Subsequently, resulting sequences were clustered into gene families using mcl [56,57] with the inflation parameter *i* = 6 selected. In total, 13,317 clusters were produced with at least six species, with downstream analyses parallelized implemented through gnu-parallel [58]. A multiple sequence alignment of these gene clusters was performed with MAFFT 7.0 [59], gap masked with trimAl v 1.2 [60], and sanitized by removing sequences having fewer than 50 amino acids or <25% unambiguous sites with the scripts *Al2Phylo.py* (-m 50 -p 0.25) [52] and *paMATRAX+.sh* [54]. Gene family trees (GFT) were calculated using IQ-TREE v. 1.6.10 [61] and the LG+R4 substitution model, with the resulting GFT analyzed in search of groups of orthologs with at least 12 species using UPhO. In-paralogs, duplicates and/or isoforms among retained orthogroups were resolved in favor of the longest sequence, with a total of 1982 orthologs retained. Similarly, these orthologs were aligned and sanitized as described above, but with only one sequence retained per species (option *-r* in the script *Al2Phylo.py*).

Phylogenetic inference of orthologous gene trees (OGT) was computed using 1000 ultrafast bootstrap resampling replicates [62,63] and substitution models and heterogeneity suggested by ModelFinder [64], based on the Bayesian Information Criterion (BIC). Cleaned sequences recovered from the collection of OGTs were concatenated in a supermatrix partitioned by locus, using the script *geneStitcher.py* [54]. Maximum likelihood (ML) analysis of this supermatrix was also conducted with IQ-TREE using the precomputed best substitution models from the collection of OGTs (-spp partition.nex). We estimated a species tree with ASTRAL-II [65] using the collection of OGTs to account for potentially deleterious effect of concatenating loci. The resulting ML tree was calibrated for downstream analyses using the penalized likelihood [66] as implemented in the chronos function of the R package ‘*ape*’ [67,68], under relaxed and correlated models, with lambda = 1.0. We set the crown age of the Scorpiones to 430 Mya, the stem age of the Buthidae to a minimum of 120 Mya [37], and the split of *Hadrurus*-*Hoffmannihadrurus* to a maximum of 11 Mya, based on calibrations from our previous studies [37,69]. To compare venom evolutionary regimes within the Hadrurinae against other families, we aimed to test the monophyly of two more scorpion families (Bothriuridae and Urodacidae) by the inclusion of more than one representative species. Our selection included all species of *Cercophonius* (family Bothriuridae) used in our previous studies, and four species of *Urodacus* (family Urodacidae).

### 5.3. Venom Transcriptome Analyses of Hadrurus Concolorous and Hoffmannihadrurus Aztecus

Transcriptomes of *H. concolorous* and *H. aztecus* were annotated with the Trinotate pipeline [50]. Venom components reported from the transcriptome, proteome, or isolated from the venom of *Megacormus gertschi* [21], *Hoffmannihadrurus gertschi* [29], and *Hadrurus spadix* [31] were used as queries to search our two transcriptomes with the *blastp* algorithm. Matching sequences with low e-values (<1 × 10^−15^) were selected. Subsequently, these sequences were used as queries to search the GenBank and UniProt databases, keeping only hits with low e-values (<1 × 10^−10^), high query cover values (>70%), and percentages of identity (>50%) as definitive matches (Tables S2 and S3 and Figure S7). To assess the diversity and composition of Hadrurinae venoms, we selected sequences encoding, or putatively encoding enzymes (phospholipases, hyaluronidases), protease inhibitors (Ascaris-type, Kunitz-type), ryanodine receptor ligands (calcins, DDH), and sodium channel toxins (NaTx) from 24 scorpion libraries. To test our recent hypothesis that calcins retain a phylogenetic signal above the familial level [36], we retrieved calcin sequences from each species used in the phylogenomic analysis to generate an ML gene tree.

### 5.4. Gene Tree Analysis and Molecular Evolution of Scorpines

Scorpine (long scorpion toxin) homologs were retrieved from the complete dataset generated here, or from UniProt and GenBank (Table S4). Signal and mature peptides were predicted using SpiderP from Arachnoserver [70]. Outgroup taxa for gene tree analysis consisted of scorpion potassium channel toxins (KTx) α and β, with αKTx used to root the tree. Phylogenetic relationships of scorpion KTxs have been addressed elsewhere [16,22]. Multiple sequence alignments for the full precursor (ca 97–124 amino acids) were generated using MAFFT and subsequently trimmed with trimAl, resulting in a matrix of 107 terminals and 77 amino acid sites. ML analysis was performed with IQ-TREE as stated above. Additionally, a phylogeny was generated using Bayesian inference (BI) for the same matrix, with the LG model [71] in MrBayes v 3.2.2 [72]. The analysis was run four times, each with four Markov chains that sampled every 1000 generations for 1 × 10^7^ generations, using default priors and discarding 2 × 10^6^ generations as burn in.

Multiple sequence alignments of nucleotide sequences encoding mature peptides were generated based on their corresponding amino acid sequences using PAL2NAL v. 14 [73]. The resulting codon alignment was used to calculate synonymous and non-synonymous substitution rates under different models implemented in HyPhy 2.6 [74]. Detection of sites evolving under positive or negative selection was accomplished with FUBAR [75] using default parameters, whereas MEME [76] was used to detect episodic or diversifying selection at individual sites in amino acid sequences.

Lastly, molecular weights, net charges, and isoelectric points of SLPs were calculated for mature peptide sequences using the R package ‘*Peptides*’ [77]. We investigated evolutionary dynamics of these protein traits for shifts in trait regimes using a continuous multivariate Ornstein–Uhlenbeck (OU) approach implemented in the R package, *l1ou* [45], as in other studies on venom evolution [36]. These data were mapped to the phylogeny and the best shift configuration was estimated and selected using the phylogenetic Bayesian Information Criterion (pBIC). Edges were painted according to their corresponding regime with statistical support for each regime shift assessed with 100 bootstrap resampling replicates [36].

## Supplementary Materials

The following are available online at https://www.mdpi.com/2072-6651/11/11/637/s1, Table S1: Putative venom sequences encoded by 96 transcripts of the *Hoffmannihadrurus aztecus* transcriptome, Table S2: Putative venom sequences encoded by 74 transcripts of the *Hadrurus concolorus* transcriptome, Table S3: List of the 24 scorpion species used in the phylogenomic analyses, Table S4: One hundred and seven sequences of Scorpine-like Peptides (SLP) isolated from venoms, or deduced from cDNA or transcriptome analyses of 44 scorpion species, Figure S1: ASTRAL-II topology, Figure S2: Chronogram under the correlated model, Figure S3: Chronogram under the relaxed model, Figure S4: ML gene tree SLPs, Figure S5: Site selection analyses of both SLP1-2 combined, Figure S6: Visualization of PCA and Correlation tests, Figure S7: Multiple sequence alignments of calcins, Kunitz-type inhibitors, and La1-like peptides.
