# Supplementary material for: Hadrurid Scorpion Toxins: Evolutionary Conservation and Selective Pressures
Source: Toxins (Basel). 2019 Nov 1;11(11):637. doi: 10.3390/toxins11110637 (PMC6891616; doi:10.3390/toxins11110637)
Supplement: Supplementary file 1 [file toxins-11-00637-s001.zip › toxins-610798-supplementary conversion/toxins-610798-supplementary conversion.docx]

Supplementary Materials: Evolutionary conservation and selective pressures in hadrurid scorpion toxins

Carlos E. Santibáñez-López, Matthew R. Graham, Prashant P. Sharma, Ernesto Ortiz and Lourival D. Possani

**Table S1.** Putative venom sequences encoded by 96 transcripts of the *Hoffmannihadrurus aztecus* transcriptome. In bold: Signal peptide. In italics: propeptide cut. Asterisks indicate stop codons.

| **Ascaris-type inhibitors** | |
| --- | --- |
| Haztecus\|153797 | **MKFNTVVCTFTILVFCSVLENTLG***QRDRR* |
| Haztecus\|251331 | FFFFFSFTVLRMKFNTAFSTFAMFVFCSVLVNSAAQQESRCRRPGEEFTMCGTACPLTCDNYQRPPEACILLCVAGCFCKNGLVRDTQRDGRCVRQSECGK* |
| Haztecus\|375070 | **MKWNISLAALVFLLCCLSAKC***MIMRR* |
| **Calcins** | |
| Haztecus\|000605 | **MKTSSLTIIFIAVIITIICLNIHDVEA***REIEFNAGRVVR*SEKDCIKHLHRCRENKDCCSKKCSRRGTNPEKRCR |
| **CAP Superfamily** | |
| Haztecus\|024941 | VGCGVSVYYDNMDNMNKVLYTCNYGPAGNMRGDAVYNVGAPCSQCPKNTQCSNEYK |
| Haztecus\|122761 | **MTSMIIPILALWIIITGTSA**YYDCDEKYTNITLDHTMCKIANESCRFLRKGKTFAAQLLRTHNNIRNSIHRFVGKEYPLATNMELMNWDEELYKIARIHSIQCIEEPDCNLCHQIGDFPVEQNFAVKKFKKSEVENNGPVKRFQTVIKEWAAELRLYDPSVVSNLTITDGLPVNWINILRATTLFVGCASMNFYSDEPGIFKEVYICNYGPAKLTEGEEIYKTGDMSCSNCKDDGMCDTEFKRLCVPNDFEINIRTTFSQNSTRYPWYYRNESYYKETTTNEENSSWEEITETLMETSIVEENATFTVTEIVPSEETSMEEVTEFLSLEETSMEEVTEFLS |
| Haztecus\|157683 | *MAIFGTKVLIKR*QLLNSAVVQICLILLVSAYPRLQRPKLYGNAIPLRDLDTRHFKTRRKIVLLHNSYRARVDPPASNMLSMAWNDEAAKDAQRWAESCRFLIHDSPTGRWVRNYGSCGQNIFVSNMKVSWSFAIKAWNIERYDFVYGSHKNVPSVVGHYTQMVWYKTHKVGCGFHYCGPNVVKKPFYNYVCNYCPIGNDPVTFNEPYGIGKPCSKCPGKCKYKKLCTNSCKYGDSWSNCAELNATWNNWLCGDPKQQRYQNCLGTCRCSSNYIR* |
| Haztecus\|168586 | KYLLRNRFSNNIHVYETALACQLSFNVAFNITFCYSVTMNLFLAILCLQLSSLPYVPCEVCSRSSYGKYDISLSHENTRDEKIFLKELKMSSWDEESSEEQITEMKECPELYERYSTEHTYCKVSTCDIITEGINEDDKELILKIHNELRNKLANGSEERYRPLPSAANMMELEWDDELSRVAQAHASLCVFRHEKNAQRAVENFSVGQNLILSPRAQKDWNVVEKLYKEEVCFFFPEYIKPFHYEGNFGHFSQLTWATTWKIGCGFAAFRENGKTKSLYTCNYGPMGNMLSGTHYIVGEPCSKCPKNTERSEQYPALCKSKTEDGPQMERPSTEDYILYCDFSDEDPQECNDVKITGSRNLTTRHIYSGNYKTVVLERSEFVSVDFGTAQDEKGICAFLYIRFGPNNANDSIASVLEISYSSDVILPGPPTKIHPLSSSFFTAAVSVNYGGPLKVTAKMRAEENAAPQYFDIKFWGIRKGSCKLFRIIQ* |
| Haztecus\|168601 | **MNLFLAILCLQLSSLPYVPC**EVCSRSSYGKYDISLSHENTRDEKIFLKELKMSSWDEESSEEQITEMKECPELYERYSTEHTYCKVSTCDIITEGINEDDKELILKIHNELRNKLANGSEERYRPLPSAANMMELEWDDELSRVAQAHASLCVFRHEKNAQRAVENFSVGQNLILSPRAQKDWNVVEKLYKEEVCFFFPEYIKPFHYEGNFGHFSQLTWATTWKIGCGFAAFRENGKTKSLYTCNYGPMGNMLSGTHYIVGEPCSKCPKNTERSEQYPALCKSKTEDGPQMERPSTEDYILYCDFSDEDPQECNDVKITGSRNLTTRHIYSGNYKTVVLERSEFVSVDFGTAQDEKGICAFLYIRFGPNNANDSIASVLEISYSSDVILPGPPTKIHPLSSSFFTAAVSVNYGGPLKVTAKMRAEENAAPQYFDIKFWGIRKGSCKLFRIIQ* |
| Haztecus\|232457 | CLLENSDKKCSMAQKFIIIVLLIVSTGMISNAAMERPMEIMVKETAIKLSNEQKLHIVYLHNVYRRNATPSAANMVYVQWDEALAITAQAWANLCVMKHGIPASPKYDKGGYGQNLYFGPSNSTYRAIWLWYNEFHNFHFRSVNCTGIECGHYIQMMWWETKFVGCALSQCGKSEFFFVCHYYPQFVNNTTPYLVGEPCSLCDLGYGGLCYDNLCVTKPACEKYGLDCKCKLKCHNCGRVNTSTCSCKCVDGWDSLDCSSQCRDQDEKCGKKDGFSSILSCSMHKGKVKKKLCRKMCQACGSVNTTNLSRTCCDGIICSPGYVIDLDNRPCSCRPLCPGPECYSSVGKIWKDESFSLAFGILALLWKTWKV* |
| Haztecus\|237566 | RGVTEEDKKIILDIHNKFRNKIALGQETSPRQQPPAANMIQMEWDNELAKIAQAHSDQCVFEHDNAPQRQVENFSV |
| Haztecus\|293319 | **MIVQLLLSFALFSWDVASS**QTCPEIYLRFSKDHTYCLLSNCHVTKRGVTEEDKKIILDIHNKFRNKIALGQETSPRQQPPAANMIQMEWDNELAKIAQAHSDQCVFEHDNAPQRQVENFSVGQNLLITMLSKTINWRKISMWYTSEINYFYPQYRQPFTFATAYGHFSQMVWAETWKVGCGVSVYYDNMDNMNKVLYTCNYGPAGNMRGDAVYNVGAPCSQCPKNTQCSNEYKGLCKSLTPDGPQKEISISSRDFLLYCNFSVNDSPGCRNVQISGSKPFQTKKLYSGEYKTAILNGGESITIKLGKAQDNRGICPFVYGSFGPNRDGDAKRSAVSIGFSAPRIMFGDPVKIEYGSSEFWTIGMLMRFSGEMESTIKLQAYPGASPQYFNVKSFGIGRGKCPKF* |
| Haztecus\|304512 | GQNVAHHWSYNYKDPVQKDKADWVFSIGHWSSEYEKFRFNPAHISPFKFNNKVGHFTQMI |
| Haztecus\|375728 | *SVNRMESYLRSFVTLVVLSTQSKFHFATEVVSFRENSTYSWIHQATDRHSLKPSRWARQAITLSESERKKAVHLHNLYRSMVIPSAANMEYMEWDHR*LAALAQRWAENCQWKHGNPPHKFPNGVGQNLYKGPSAYISTAMGLWFDEYKDYNYFNIKCKPYKKCGHYTQIVWATTRYVGCAVVSNCWPDYKYYTVCNYWPPGNYQNTKPYIEGKPCTRCNFTGNGLCSKNSCVSRDQCKRH |
| Haztecus\|387397 | *FTQMIWGETR*MIGCGYVFYVHPKQGYTKSYICNYAPGGNVIRGIMYKISPEGAKCSFPVLHPSKEYQGLCENGR* |
| **KTx** | |
| Haztecus\|368213 | **MAKSFFAAFLIIMLISSLVDG**KSTVGQKLKKKLNQAVDKVKEVINKSEYMCPVVSSFCKQHCARLGKSGQCDLLECICS* |
| Haztecus\|092578 | **MRLAIILLLMTILVLTVG**APPSGTKCSSSNQCTRPCRYGGGTHGKCMNGRCRCYG* |
| Haztecus\|315193 | **MKFILILFLLSIYMSTCFPSIEG**IKCYDSGWCREPCRHKGATYGKCINNSCMCYR* |
| Haztecus\|139672 | **MNTKLVLMMLMITAVILVFEAETVTG**TGTSCINPKQCTEPCRAKGCKHGKCMNRKCHCMLCNR* |
| Haztecus\|224197 | **MNTKSICFVFLVTLMLAFDRIEVEG**VKKCSVASTCVKYCWKYEKCSRGKCINKECKCYNCRG* |
| Haztecus\|364908 | VKKCSVASTCVKYCWKYEKCSRGKCINKECKCYNCR |
| **Scorpines** | |
| Haztecus\|062933 | **MEIKLTILILLVITSFCSC**GILREKYAHKAIDVLTPMIGVPVVSKIVNNAAKQLVHKIAKNQQLCMFNKDVAGWCEKSCQQSAHQKGYCHGTKCKC |
| Haztecus\|083866 | **MNTKLTVLCFLGIVTIVSC**GWMSEKKVQGILDKKFPEGIIRNAAKAIVHKMAKNQFGCFANVDVKGDCKRHCKAEDKEGICHGTKCKCGVPISYL* |
| Haztecus\|106327 | **MQLKFLVLLLFGMTTFCTC**GILKEKHFHKAVDYLVPAVVPDVVKNLAKQAIHKIAKSESFCMFGQDVRKLCNKSCMETLNMKGNCHGTKCKCEP* |
| **Kunitz-type inhibitors** | |
| Haztecus\|369762 | **MIIFYGLFSILVLTSINVGEA***GHHNR*VNCLLPPKTGPCKGSFARYYFDIETRSCKAFIYGGCEGNSNNFSKKHHCEKRCRGFRNFGGK |
| Haztecus\|016332\| | MSNIKPWMPNMAVINVIYWPLFSDACNQPPNPGLCFARFERYYYDPSTNTCKLFVYGGCKG |
| **La1-like peptides** | |
| Haztecus\|090690 | VQRNIFPIIPETCMTDGITIPLNEEKQDQTKCVLFRCEKNAGRIVLNTITCAPQEPKRGCQNIISPVNLPFPDCCPLVVCKHPIFYNGYK* |
| Haztecus\|090697 | NDFRNMTKSTAATFVILFASTYFVSCLAYGETCMTDGITIPLNEEKQDQTKCVLFRCEKNAGRIVLNTITCAPQEPKRGCQNIISPVNLPFPDCCPLVVCKHPIFYNGYK* |
| Haztecus\|106766 | **MVHAFTPILFGILLTLSFSSLSTG**YGESCQAGKYTIHIGRPVQDSKSCTLYKCINYNRRYSLETLTCAKMTLKTGCRYVPGAATARFPDCCPMVVCRGSG* |
| Haztecus\|166900 | **MKHLHLYLVFGSLSLCALLSMSFG**AGEYCQVGEISIPVGGQKQDSKECMLHKCVNQNNRILLDSFSCAPQEAKRGCRNMPGPIDAPFPDCCPISVCRGKQWDK* |
| Haztecus\|294468 | YTTLFRSGEYCQVGEISIPVGGQKQDSKECMLHKCVNQNNRILLDSFSCAPQEAKRGCRNMPSPNGA |
| Haztecus\|132887 | **MMKTTLVLVFLFAFTYTANA***GLVTRR* |
| Haztecus\|118284 | *FLFAFTYTANAGLVTRR*TCRNRNGGIMQEGEIWKDPNHCSVYRCVIYDNEAELIGMTCAEFQVQNDCKKVPGR |
| **Phospholipases** | |
| Haztecus\|009989 | *SRIRR*SIMQLAGMLRCTTGCDPLAYKSYGCYCGYLGAGRPVDDIDSCCREHDRCYSNTICPQLFLYFFRYPWRCTSLGIPQCGRMPGVTIAQQCARQLCLCDREFVRCIGRFPCPRQSAICRTNPLGVLQSLIVGKR* |
| Haztecus\|027670 | MLKKTELTKRKWNGWIKPSCIPITIIFILIVLVVMLPLLDQKHEDKVHLLTNPSNCTEPCWATLVESIPENMTYNISLNHPSTFEGWLNLIQLAEHTIDIAAFYWTLRGRDVISDPSDWQGEKIFDDLLAAGTERKIKISIVQNLPSHIFPDNDTKELEEKGAAAVRNMNFEKLLGKGILHTKMLIVDDKHFYVGSANMDWRALTQIKELGIIVYNCSCLAKDVKKIFDAYWLLSETEHIPLPWPSYFDTTFNKTHPAVLTINNTSTYAYSSSSPPQFCAKGRTSDINSILSVIHKAKKFIHVAVMDYFPALIYTKHLKYWPVIDNALREAAITRRIKVKVLASYWKHTRPVMQAFLRSLQSLNSTDISIETKLFEVPVYSPSQEKIPYARVNHNKYMVTDNSAYVGTSNWSGDYFISTGGVGFILENTDNSTNSSIQIQLQDIFERDWSSEYAHPITEIDIR* |
| Haztecus\|077087 | **MRNFEERKYGRCIMLLLKLTVFILLFVQPAPS***TDLPLPLPHEKKLTAFYQEKQNPYMLIIGR* |
| Haztecus\|077100 | *MRNFEERKYGRKEADRVLAALHLDDVEKIEHRR*MESLTKFCAKEKHKFIPKTYTKKVFIYPGTKWCGMGDNAANEDELGKEKEADSCCRDHDHCKDSIPAFSIKHNLTNYSPFTKSHCDCDKQFHTCLAMARTKTATIISNLYFNLLNMECFEHKTCSSNETCITWQWKLAKSDGSPIQ* |
| Haztecus\|134320 | *MTASPQPQIKTVHQLQNMLALFWKSVNSNYNKMPSSYVTGLFKTKQWKNAVKNWKISEFAKIVGIMQFHWKFPILLQSKINKNTENLPILKSDTQRAPVTKKTEPTQILELDKALTDWINDDKKPDNNLSFQDNRNSLDIIKSKPTSSVLGNLEKVLSSWFQGDALTFGFQTSKNWMMAQKDESSLTTTYKVTKINVESR*SRFLVKSLSSASSETSILYRLQEMGKHLLRYPDQKDIMVKENAIPVTLRIRESFSNTIQAKAQEILSLLGYSDPPGSAGVRILSIDGGGIRGMIAIEILRYLENVSGKPVYQLFDYICGVSTGAILSMLVGGLHLPLDECERLYVKMSSELFKRSAFLGTSGLIWSQSYYDTSMWVNMLRKTYGEIMLSETCNKKGNPKISCVSAVMNQPILEAYAFRNYSLPYRKQSLYPGSSNHKLWQAIRASAAAPGYFEQYTLAGIVHQDGGLIMN |
| Haztecus\|139911 | **MAFLCLTTLLILISLRNA***RIIQRELYLNLEPVSNQKDLLPVARAAVVNFDEGFETGR* |
| Haztecus\|162449 | **MNILFIAVFLNIITNFDVKS**QSILRGVQKALDSVSNFIDDISFGIKQVRDGLSTVDDIVNYAQGKPCQYICPPGLMLKKNYYYKPIPQGCGAYGIQVVLSLPLLKDTEKCCDKHDICYSTCMTNKTDCDAEFGTCLYKKCEKQAKKLGEDVRKCTGVSKLFHLGVQSLGCNAFKAAQAEACRCASKDEL* |
| Haztecus\|183750 | **MGLIMVLVIGVLSADAVLS***MDNELYLNLEPSQRSSWPVARAVRMQFSKRSEGGRESRR* |
| Haztecus\|305099 | **MTFWCTLILLTLLTVCCRINECRS***LHRKPRSLPQLARMVEATTGR* |
| Haztecus\|311460 | DDYDDLGVYEETDKCCRTHDHCNDSIVGFETKYDLKNKDFYTKSSCNCDLRFHSCLYKKEAIHSDAVGHLFFNILQTQCFKDEYPIVKCLKKWGIPLIRETCQKYKLDCNGTKKYQFFDAKMYKGKNESPFLKKLLSH* |
| Haztecus\|330410 | **DTPCSNEEAIMKTSLVFVLATLSLG**ECVIFDSVSDVLPLTTTFYREKDGHRMVETIEVNPYLSEKKTIDCYMYGNNYIIDRMLELVPESVTKESDKTDISKLVNQCSDLLLNELNNGIFHSVKSPFDSVRNAFKSLLIFPGTKWCGAGNVADNYEDLGRAEQTDKCCRTHDHCNDTIPGYETKYGLKNENFYTKSTCPCDLTFHSCLYEGNNLPSDLVGKTFFNILLMQCFKKEYPQIGCLEKTGIILRSCQEYKFDYNGTKKYQFFDAKTYEPKENASFLSRLLSG* |
| Haztecus\|355671 | VNYTRYNLLEEKCRDFHKLIQASLGRRRIRRELFLYPGTNWCGFGSSVKKFGELGYNTHADRCCRDHDLCPFTISAFSKRYDLFNYRFHTISHCECDERFRACLKLANSPTANIVGRLFFNIVQIKCFSFRVENVCSKWSWWGRCLLNENKTTASLKG |
| Haztecus\|384314 | EDKCCREHDLCDDIIQAGGRKDDLVNDSKYPTLLCSCDDRFWHCLRDINSLTSNAIG |
| Haztecus\|402536 | IEDPVSYISFYGLRNYSMLNDKLVTELVYVHSKLMIVDDRIVIIGSANINDRSMIGK |
| **Hyaluronidases** | |
| Haztecus\|032949 | *WMLKTLELTKKMRPKGR*WCFYHLPDCYNYAGKDKPEQFLCSSLVRKHNDRLIWLWNATTALCPSIYFDERQTKY |
| Haztecus\|032956 | *KYNESQQIWFIYGR*LSEVLRVSQPRTPIYPYINYRIHSSLEEVPKERFWLMLGHLASLGLDGAVIWGSSNYVKTEEECRNLELYVKEVIGPASSTISSNVNRCSEVICNGEGLCTWPHQSYTSWKYLTNQNASAFKREDITCR |
| Haztecus\|047421 | LGEKLVIFYENKLGKYPYIDPKYGDVNGGLPQLGNLEKHLQAAEKDIQTIISNPNFDGLGIIDWEKWRPIWDFNWGKMRIYKTRTMELMQKKHPSWPWKLIENASRNQWEETAKQW |
| Haztecus\|047425 | *ILFDVPELANLSEHLEIARR*DIEKTIPDPNFDGLGIIDWEKWRPIWDFNWGKMRIYKTRTMELMQKKHPSWPWKLIENASRNQWEETAKQW |
| Haztecus\|085528 | LFPDCYNYEGKDQPSQFFCSKNVQTYNDRLSWLWEASTSLCPSIYFYNSQMKYSDHQRLWFLYGRLAEAVRVSSPSTLIYPYVNYKSPDILSDVPEEHFWRMLSLSASMGLDGVVIWGSSNYVQKKEDCKALASYVKKVIGLSSLTVSKNFNFCSKTTCRGQGRCVWPEEPYTSWRYMCNRDLSDFQPGEIICRCQTKKGRYCNLSNVKLN* |
| Haztecus\|136074 | MLWLVRTIAVFSIFIFGGKANFNVYWNVPSAPCSKKYGINVTDDLMKHRILVNNGEQFIGNKIVIFYENKFGKYPYIDTEKGIDVNGGMPQLANLTEHLKTAEKDIDNMIPDPDFDGIGIIDWESWLPIYDYNWGKRSIYRTRSIKLIKKMAPPLHAEIIKRIAGKQWERIAKQWMLETLELAKKKRPKARWCYYLFPDCYNYKDKQKQSEFCCRESVRANNDRLSWLWEASTALCPSIYFYNKHMNYTYHQRLWYVHGRLSEAVRVSSGKALIYPFVNYLCRDKDNNILFDVPEKHFWQMVSFTASMGLDGVVIWGSSNYVKRKKDCEALASSVKKVIGPSSLTVSRNFKRCSVIICSGVGRCFWPEKQFVSLTYLSNKDLPQTFFKREETACRCKKNLGRYCNVSNLDPHHLNEDCPEVH* |
| Haztecus\|136097 | MLWLVRTIAVFSIFIFGGKANFNVYWNVPSAPCSKKYGINVTDDLMKHRILVNNGEQFIGNKIVIFYENKFGKYPYIDTEKGIDVNGGMPQLANLTEHLKTAEKDIDNMIPDPDFDGIGIIDWESWLPIYDYNWGKRSIYRTRSIKLIKKMAPPLHAEIIKRIAGKQWERIAKQWMLETLELAKKKRPKARWCYYLFPDCYNYKDKQKQSEFCCRESVRANNDRNISGKWFLSRHPWV* |
| Haztecus\|136098 | *MVSFTASMGLDGVVIWGSSNYVKR*KKDCEALASSVKKVIGPSSLTVSRNFKRCSVIICSGVGRCFWPEKQFVSLTYLSNKDLPQTFFKREETACRCKKNLGRYCNVSNLDPHHLNEDCPEVH* |
| Haztecus\|178545 | **MLFFVCIVSIFNNIEA**SFDVYWNVPSHLCSIKYDVNMTETLSKYNILVNDGESFTGDKITLIYENGIGKYPHIDPNKGDVNGGLPRLDKLKEHLNLAEKDIEKIIPNPDFSGLGVIDWEAWRPIWEYHWGGLSIYKSRTIDLVKKDHPTESDQFIETTAKNLWENTAKQWMLKTLELAKKLRPKGQWCYYLFPDCYNYFGKDHPSEYFCSTMIQNNNDRLSWLWDASTAFCPSIYFIENQMKYNESQRTWFLYGKLAETARVARPSTKIYPYINYMVHVSQIPVPRDHFWKMLALIASMGFDGAVIWGSSSYLGSEKSCNDLESYIENVIGPAVTTISSNVDRCAQMICNGRGRCTWPNEPFISWQYLTDTNGPNFDSQKITCRCQSHSGRYCS* |
| Haztecus\|317745 | FDGLGIIDWESWRPIYNYNWGTMTIYKTRTVELVRKENPSLKVDLIKSIAEKQWEEIAKQWMLQTL |
| **Metalloproteases** | |
| Haztecus\|025479 | LLHGVHKPVKLCASANGKFDVGRAKSMARMFGQQRALILVFFATIGLSGQQDDNDAHDGRQCDCMEYWQCIGAGGKPYSYCVYTNKVCCFVDRNAKSVGILPRRSKTASCGQKGIDNGREGFSEPGEWAWHAAILESPRDLYVCGATLVDEYWVMTAAHCVDDFSNVLKLKIRLGEYDVTRSSEPLRHEEFEVSRVVLHPGFDNSTLLHDIALLRLSTPAKRRRHINTVCMPETGMTDNQLFGSKCFVTGWGKRNEKSNHSVILKEVNVPLWKNSECEIALRRQFGPHYELPSSVICAGATGRDACDGDGGGPLVCEKNKNWYQIGIVSFGIGCGRPKTPGVYTRVHSYRQWIHDVILHS* |
| Haztecus\|027032 | **TGFQVHKMAVRSMCLFTTWSLLLLFFGSKQS***VSQDRR* |
| Haztecus\|030723 | QFQCGAVIITNRWLLSAGHCFVHTRDSYWVARLGLLRRGSDLPTPYEEVRRITHIEVNPQYINKNFINDIAVLKLETAVPFSNYIRPICLPEPEDDVTKWNGKKCSVVGWGKLGEQGDRFPDTLQEVQLPVISTEECRKRTLFLPIYQITDNMFCAGYERGGRDACLGDSGGPMMCQKENGRWVLIGIISNGDGCARAARP |
| Haztecus\|103421 | **MRWYQVIVVISTLFVQHVVWT***ESRAVSGEDPNEMEVEIVYPRVYHMSRKKRDVGSSDETR* |
| Haztecus\|103449 | **MRWYQVIVVISTLFVQHVVWT***ESRAVSGEDPNEMEVEIVYPRVYHMSRKKRDVGSSDETR* |
| **HDPs** | |
| Haztecus\|049505 | **MNAKIMLVVFMITMFVTDQVEG**GFGSWVKKLWKSKLGKAARKAAGNFIAKKLENAAAPAEGGSKRFDEFMNSLYY* |
| Haztecus\|049972 | **MNAKAFLAIFMIALLVTDQAEA**GWWNALKSIGKKLLKSKLAKDITKMAKQRAKEYVVKKLNSPPEEEVAAIDALMNSLDY* |
| Haztecus\|270640 | IKGPVKGNGHCLSATFVDKKMNAKVFLVVFMIALFVTEKAEAGILDTIKSIASKVWNSKTVQDLKRKGINWVANKLGVSPQAAASMTLDEIMDALEDY* |
| Haztecus\|314185 | **NPQPSVHKKMNVKVFLLVFMIALFVTEQAEA***GIWESAKSIGMQVWKSKPVEELKR* |
| **Other inhibitors** | |
| Haztecus\|048272 | VIEVNEEGSEAAGASAILIVPYSSAFSSIKSFYANHPFIFFIRDDRTGIVLFYGR |
| Haztecus\|133931 | **MLSQINSKMKLLIISCIIVVVYA**REMSECELHREKELEKKPLARLIPECEENGDYKGLQCESGTRYCQCWDKNGTSITALSMKLKACECQREKKTAEDKNLIGKFIPSCEVDGTYSKKQCVASTGMCWCVDETGNKISEPTRDEIHC* |
| Haztecus\|133935 | **MKLLIISCIIVVVYA**REMSECELHREKELEKKPLARLIPECEENGDYKGLQCESGTRYCQCWDKNGTSITALSMKLKACECQREKKTAEDKNLIGKFIPSCEVDGTYSKKQCVASTGMCWCVDETGNKISEPTRDEIHC* |
| Haztecus\|176774 | **MKAALIYVVIFTFVVATFA**KKTTECQESREKALKSKACITAIVPVCDENGDYAALQCQEGSKFCSCWRKDGTPIIQPSEKIKACECHRQKDEKSSKGLLGTFIPQCAEDGKFQKIQCWGSTGYCWCADPKTGRNTSVSIRGRPNC* |
| Haztecus\|176784 | MKLTKMIREKTMKAALIYVVIFTFVVATFAKKTTECQESREKALKSKACITAIVPVCDENGDYAALQCQEGSKFCSCWRKDGTPIIQPSEKIKACECHRQKDEKSSKGLLGTFIPQCAEDGMFQKIQCWSSTGYCWCADPKTGRNTTDSVRGRPNC* |
| Haztecus\|176796 | MKLTKMIREKTMKAALIYVVIFTFVVATFAKKTTECQESREKALKSKACITAIVPVCDENGDYAALQCQEGSKFCSCWRKDGTPIIQPSEKIKACECHRQKDEKSSKGLLGTFIPQCAEDGKFQKIQCWGSTGYCWCADPKTGRNTSVSIRGRPNC* |
| Haztecus\|190709 | LHSIISQTSGQVIYKYDTKTGYPLSNFCINFAVSSQTITVLLSRSFFRLTMKILLALAVLTVTVSCQPQFGGTGCKPYEEYREASCEVTCDKVLSVTCLTAEKRPGCYCKIGTIRDEDGQCISTEVCSKRVCTKKNQRLDMSGCFTVCTGLGTSYFGCPFVQQPGCNCERGFAVAGGGIRGDCIPVSSCRDPNLDN* |
| Haztecus\|349912 | MKSAFSDEADFSGISEKYELQISAVIHKAVVEVNEKGSEVAAGTGIVIKPCSARLPSRHYFRDGNRAGMILFLGRVNDL* |
| Haztecus\|365265 | *WEETVFFSPASLFIGLAMLYRGAR*SKTSAEMSHALSYDVAGLEATNLHRRIR |
| Haztecus\|377457 | MKPNLETLGMRSTFRNDANFKGISTKANLKVSKVIHKVIVEVNEAGTEAAGATALTVVPYMLQETWYFNVDRPFLFYIRDDVNGVILFAGRINQL* |
| Haztecus\|382099 | **YSCLSSRSDKIKMKLFILVCFMLVTLSLA**EQTPCQAKREKILNQNLDVEVIPECDENGNYKAKQCKKNGVDCQCWRTDGTPINDFSPNLKACSCVRSRDDANRPHLIGNYKPQCEADGTYSLTQCWGSVGGCWCVDAEGRKLTDKHFP |
| **Other venom components** | |
| Haztecus\|018156 | **MRLYLFVLAVLIASCHC**APRNRCTQECGPVPSNCRAGVTKDYDGCCAICAKAEGEECGGMWNAYGVCGIDLICDTNGNLISNYDLPIGICTSARRIASRNILKRTLRGLH* |
| Haztecus\|031281 | **MGFKFCFTAFILTAIFVNVMT**LRCRVCGTYECLPPPTNCPVGTVTDVCNCCLVCGKAENEICGGDWDLRGKCGNGLRCVKTGKTGVCKKE* |
| Haztecus\|068800 | VSDSIRVFSVFSGVITDEGLICSCNDVLCQETGKCALGEVKGVCECCNECARVRDEPCGGIYNYAGICGTGLKCEPNDFKQLPGICIPEK* |
| Haztecus\|068805 | **ELLTILAYKLSLFFVDLTSQIKDLKMLYFLTFLTISIA**LCRSGVITDEGLICSCNDVLCQETGKCALGEVKGVCECCNECARVRDEPCGGIYNYAGICGTGLKCEPNDFKQLPGICIPEK* |
| Haztecus\|075169 | **MFRTTLLSLLVASAYS**LICEPCIFEDCNDVPTCPLGVTKDVCKCCDECFKMEGEICGGPYNVSGICGEGLRCNKGVELYPFHFRNAFGICTLKT* |
| Haztecus\|076767 | **MLRLILLCILVASVYS**LSCPCWYDEDKTKYCPPPPTNCPIGLTLGPCGCCLECYKDKGEVCGGSWQILGKCGGGLKCEKGFNDLGSDYYYANHKEGVCQPIEPIDPLIE* |
| Haztecus\|078916 | **SCKQDRSKMWFRFIVLFLFVAGVYS**LSCPCHTNRLLCKPAPTNCKLGLTKDACGCCDVCYKIEGEECGGPWKTSGNCGKGLKCVVPENLPEHVKWQAIGICKVE* |
| Haztecus\|110763 | **MFRLILLCMLVASVYTL**SCPCLHELDRTKNCPPPPANCPLGLTTDACGCCPVCYKDKGEACGGPWKIIGKCGKGLTCVKETSVSKPKGYYIDQIAGVCKPIAIN* |
| Haztecus\|110769 | **MFRLILLFISVASVYSL**SCPCWREPDKTKYCPPPPTNCPLGLTTGPCGCCLQCYKDKGEACGGPWKIIGKCGKGLRCVKETNVGKPKRYYIDQSEGICQPIDTY* |
| Haztecus\|133931 | **MLSQINSKMKLLIISCIIVVVYA**REMSECELHREKELEKKPLARLIPECEENGDYKGLQCESGTRYCQCWDKNGTSITALSMKLKACECQREKKTAEDKNLIGKFIPSCEVDGTYSKKQCVASTGMCWCVDETGNKISEPTRDEIHC* |
| Haztecus\|133935 | **MKLLIISCIIVVVYA**REMSECELHREKELEKKPLARLIPECEENGDYKGLQCESGTRYCQCWDKNGTSITALSMKLKACECQREKKTAEDKNLIGKFIPSCEVDGTYSKKQCVASTGMCWCVDETGNKISEPTRDEIHC* |
| Haztecus\|159042 | **MRIKFCLITFVFLGIFSSATS**LTCLICGTFECSPPPNNCPAGLVKDACGCCLVCAKAENESCGGLSNIFGKCGHGLKCVFEGNPVITSGICKKSG* |
| Haztecus\|176774 | **MKAALIYVVIFTFVVATFA**KKTTECQESREKALKSKACITAIVPVCDENGDYAALQCQEGSKFCSCWRKDGTPIIQPSEKIKACECHRQKDEKSSKGLLGTFIPQCAEDGKFQKIQCWGSTGYCWCADPKTGRNTSVSIRGRPNC* |
| Haztecus\|176784 | **MKLTKMIREKTMKAALIYVVIFTFVVATFA**KKTTECQESREKALKSKACITAIVPVCDENGDYAALQCQEGSKFCSCWRKDGTPIIQPSEKIKACECHRQKDEKSSKGLLGTFIPQCAEDGMFQKIQCWSSTGYCWCADPKTGRNTTDSVRGRPNC* |
| Haztecus\|176796 | **MKLTKMIREKTMKAALIYVVIFTFVVATFA**KKTTECQESREKALKSKACITAIVPVCDENGDYAALQCQEGSKFCSCWRKDGTPIIQPSEKIKACECHRQKDEKSSKGLLGTFIPQCAEDGKFQKIQCWGSTGYCWCADPKTGRNTSVSIRGRPNC* |
| Haztecus\|300057 | **MGVKFYFIAFILNVVFLNTMA**LKCKECGTYECRPPPNNCPVGTVTDVCNCCLICGKAENEICGGEWNLLGKCGEGLKCVKKPGSNNQLIPPKAGICKKIDW* |
| Haztecus\|339074 | **LHCITPHCIIFIDINLTNVQHSPASLFIDSQTSTVFPKLYSYTEKATHQDMGRKLCLVVFVLLGIFTSTMA**FTCLACGSYECPPLPLCTAGVVKDVCDCCVECAKNENENCGGMWEQYGKCGKGLKCVSTGTTTPDPFMWDWPLPGICQKE* |
| Haztecus\|352810 | **MEIKFCFIGCVFFGIFINALA**LTCRDCNTFTCPPPPDNCPAGLVNSMCRCCLVCGKAEDEICGGDWNLFGTCGNGLTCVMTRFNNNSPRP |
| Haztecus\|382099 | **YSCLSSRSDKIKMKLFILVCFMLVTLSLA**EQTPCQAKREKILNQNLDVEVIPECDENGNYKAKQCKKNGVDCQCWRTDGTPINDFSPNLKACSCVRSRDDANRPHLIGNYKPQCEADGTYSLTQCWGSVGGCWCVDAEGRKLTDKHFP |
|  |  |

**Table S2.** Putative venom sequences encoded by 74 transcripts of the *Hadrurus concolorus* transcriptome. In bold: Signal peptide. In italics: propeptide cut. Asterisk indicate stop condon.

| **Ascaris-type inhibitors** | | |
| --- | --- | --- |
| Hconcolorous\|008487 | **CGDNALEMKLRWVILAALLIMLTEE**IKTPSDCNPETEEWQECGTACPLTCKNYNNRPKICTRQCIMECFCKDGLIRGYNNDCVIPENCEMTQ* |  |
| Hconcolorous\|012894 | FKVRTILKMKFNTVVSTFAILVFCSVLENTLGQRDRRCGLPTEEFLTCGTACPLTCDNYQRPPRDCILPCVRGCFCRRGLVRDTRRGGRCVRPSECRR* | |
| Hconcolorous\|028862 | *VAKQGKTSHPNFIKSR*SQKHKSFQSFTILGMNFNTAFSTFAMFVFCSVLMNSAAQQDTTVAYLSEDAAKSSKLPESRCRRLGEEFTMCGTACPLTCDNYQRPPEACILLCVAGCFCKNGLVRDTQRDGRCIRPSECGK* | |
| **Calcins** | | |
| Hconcolorous\|c28713 | **MKTSRLTTIFIAVVITIICLNIHDVEA***REIEFNAGRVVR*SEKDCIKHLHLCRENKQCCSKKCTRRGTNPEKRCR | |
| **CAP Superfamily** | | |
| Hconcolorous\|001850 | **PRLFQMEKHLSIIFLLQFFCYFSVG**CKYEKLGIIHTMCVYSSGACSNGRLLHSGAISAEDKSIILMMHNRIRSYLATGNISGLPPAADMLVVEWDNELAAIAQRWADQCTNGHDELRNTEQYYVGQNVAHYWSYNYKDPVLKDKADWVFSIEHWSSEYEKFRFNPAHISPFKFNNKVGHFTQMIWGETRMIGCGYVFYVHPKQGYTKSYICNYAPGGNVIRGIMYKISPEGAKCSFPVLHPSREYQGLCEHGR* | |
| Hconcolorous\|002358 | ANSDKKCSMAQKCVIIVLLIVSTGMISNAVMERPMEIMVKETAIKLSNEQKLHIVHLHNVYRRNATPSAANMVYVQWDEVLARTAQAWANLCVMKHGIPASPKYDKGGYGQNLYFGPSNSTYRAIWLWYNEFHNFHFRSVNCTGIECGHYIQMMWWETKFVGCALSQCGKSEFFFVCHYYPQFVNNTTPYLVGEPCSLCDLGYGGLCYDNLCVTKPACEKYGLDCKCKLKCHNCGRVNTSTCSCECVDGWDSLDCSSQCRDQDKQCGKKGGFSSILNCPMHKGTVKKKLCRKMCQACGTVNITNLSRTCCDGTICSPGYVIDLDNRPCSCKPLCPGPECYSSVGKIWKDESFSFAIGIFALLWKTWKV* | |
| Hconcolorous\|002447 | **MTSMILPILALWIIIAGTSA**YYDCDEKYTNITLDHTMCKIANESCRFLRKGKTFAAQLLRMHNNIRNSIHRFVGKEYPLATNMELMNWDEELYKIARIHSLQCVEEPDCNLCHQIGDFPVEQNFAVKKFKKSEVDNNGPVKRFQTVIKEWAAELQLYDPSVVSNLTITDGLPVNWINILRATTLFVGCASMNFYSDESGIFKEVYICNYGPAKLTEGEEIYKTGDVSCSNCKDDGMCDTEFKRLCVPADFEINIRTTSSQNSTRYPWYYRNESYYVGNATNEENSSWEEVTETLMETSIVEENATFVVTEIVPSEETSMEEVIELLPSDETSMEEDIEFLPSTAPSKENTYLIKRKIRPLVSKMLKYSAKLPKGMLQKQFVTDMKRVIRHLI* | |
| Hconcolorous\|014412 | **MNLVLAILCLQLSSLPYILC**EECSRTSYGKYDIALSHENSRDEKIFLTELKMSSGDEESPEEEITEMKECPELYQRYSTEHTYCKVSTCNIITEGVNEDDKELILKIHNELRNKLANGSEERYRQLPSAANMMELEWDDELARVAQAHASLCVFKHDKSAQRQVENFSVGQNLILSSGAQKDWNAAQNLYKEEVCFFFPEYIKPFHFEDNFGHFSQVTWANTWKIGCGFAAFRENGKTKSIYTCNYGPSGNVIGQTHYIVGEPCSKCPKNTECSEQYPALCKSKTEDGPQVERPSTEDYILFCDFSDDDPQECNDVKITGSRNLTTRHIYSGNYKTVVLERGESVSVDFGTAQNENGICPFLHIRFGPNNANDSTGSVLEISYSSNVIVPMPPTIIHPHGSSFFTAAVLINYGGPLKLSVKMRAEENAAPQYFDVKFWGIRKGSCKLFV* | |
| Hconcolorous\|014413 | **VFGAIQNFTPVTMNLVLAILCLQLSSLPYILC**EECSRTSYGKYDIALSHENSRDEKIFLTELKMSSGDEESPEEEITEMKECPELYQRYSTEHTYCKVSTCNIITEGVNEDDKELILKIHNELRNKLANGSEERYRQLPSAANMMELEWDDELARVAQAHASLCVFKHDKSAQRQVENFSVGQNLILSSGAQKDWNAAQNLYKEEVCFFFPEYIKPFHFEDNFGHFSQVTWANTWKIGCGFAAFRENGKTKSIYTCNYGPSGNVIGQTHYIVGEPCSKCPKNTECSEQYPALCKSKTEDGPQVERPSTEDYILFCDFSDDDPQECNDVKITGSRNLTTRHIYSGNYKTVVLERGESVSVDFGTAQNENGICPFLHIRFGPNNANDSIGSVLEISYSSNVIVPMPPTIIHPHGSSFFTAAVLINYGGPLKLSVKMRAEENAAPQYFDVKFWGIRKGSCKLFV* | |
| Hconcolorous\|023377 | *MESYLRSFVTLVVVSTQSQFHIATEIVSFRENSTYSWIHQATNRHFSKPSRWARQAVMMSESERQKVVHLHNLYRSMVIPSAANMEYMEWDHR*LAALAERWGQNCDWKHGNPPHKFPQGVGQNLYKGPSAYISTAMGLWFDEYKDYNYFNLKCKPKKMCGHYTQIVWATTRYVGCGIVNNCWPDYKYYIVCNYWPPGNYQNTKPYIEGKPCTRCNFTGNGLCSKNSCVNRDQCKRHKLDCACDLKCYNCGEFDKENCNCKCKDGWKSHDCTEPCVDSRRCEKHECWRYKLSRTNPCESTCGICKGVNQSNLRNTCCDGVLCPYGQVYHSGDRPCVCRILCPGPKCGAFLHGPYFVLLVVMILFSGYYNKL* | |
| Hconcolorous\|032202 | **MIVQLLLSFALFSWDVVSS**QTCPEIYLRFSKDHTYCLRSNCHVIKRGVTEEDKKIILDIHNEFRNKIALGQETSPRQQPPAANMIQMEWDNELAKIAQAHSDQCIFEHDNAPQRQVENFPVGQNLLITMLSKTINWRKIRMWYTSEINYFYPQYRQPFTFATAYGHFSQMVWAKTWKVGCGVSVYYDNMDNMDKVLYTCNYGPAGNMRGDAIYSVGAPCSQCPKNTQCSNEYKGLCKSLTPDGPQKEISISSRDFLLYCNFSVNDSPGCRNVQISGSKPFQTKKLYSGEYKTAILNGGESITIKLGKAQDNRGICPFVYGSFGPNRDGDAKRSAVSIGFSAPRIMFGDPVKIEYGSSEFWTIGILMRFSGEMESTIKLQAYPGASPQYFNVKSFGIGRGKCPKF* | |
| **KTx** | | |
| Hconcolorous\|028795 | **MVKNFFAAFLIIMLISSLVDG**KSTVGQKLKKKLNQMFVKVKEVVNKSEYMCPLVSSYCKQHCARLGKSGECDLLECICS* | |
| Hconcolorous\|024751 | **MNTKLVLMMLMITAVILVFEAETVIG**TGTPCKNPKQCAGPCQAKGCKHGKCMNGKCHCMLCKRS* | |
| Hconcolorous\|024749 | **MNTKLVLMMLMITAVILLFEAETVSG**TGTPCRTPKHCAEPCKAKGCKHGKCMNGKCHCMLCKRS* | |
| **Scorpines** | | |
| Hconcolorous\|008061 | **MEIKLTILILLVITSFCSC**GILREKYAHKAIDVLTPMIGVPVVSGIVNNAAKQLVHKIAKNQQLCMFNKDVAGWCEKSCQESVHQKGYCHGTKCKC | |
| Hconcolorous\|029426 | **MQLKFIIFFLLGMATFCTC**GILKEKHFHKAVDYLVPAVVPDVVKNLAKQAIHKIAKSESFCMFGKDMRKLCNQSCMETLNMKGNCHGTKCKCEP* | |
| Hconcolorous\|033550 | **MNTKLTILCFLGIITIVSC**GWINEKKVQEALDKKLSDGVMKSMAKAIVHKVAKNQFGCFANVDVKGDCKRHCKSEDKEGICHGTKCKCGVPISYL* | |
| **Kunitz-type inhibitors** | | |
| Hconcolorous\|102082 | **MIIFYSLFSILVLTSINVAEA***GHHNR*VNCLLPPKTGPCKGSFARYYFDIETRSCKAFVYGGCQGNSNNFSKKHHCEKQCRAFRYFGGK | |
| **La1-like peptides** | | |
| Hconcolorous\|010016 | **MKHLHLYLVFGCLSLCALLSMSVGAG**EYCQVGEMSIPVGKQKQDSRECILHKCVNQNNRIVLDSFSCAPQEAKRGCRNVPGPVDAPFPDCCPISVCRGKQWDD* | |
| Hconcolorous\|031288 | **MAHALTPILFGILIMFSISSLSTG**YGESCQAGKYTIHVGRSVQDSKSCILYKCINYNRRYSLETLTCAKMTLKSGCRYVPGPATARFPNCCPMVVCRGSG* | |
| **NaTx** | | |
| Hconcolorous\|028874 | **MMERRFVFILFLAAFIYEIRNVEG**KDGYPLTVKGLKYSCVAGMVIGDNRFCESICYDEGSTYGYCYGFGCYCEGMRDDVKVWGE | |
| **Phospholipases** | | |
| Hconcolorous\|000043 | **MWTTTAILFLSAFLVAES**GIFDIVDKVLPITTTFYREKNGHRMVEIIEVNTYIGGKKLVDCYLYGHLYIIDKMMELVPSDIVKYVNKKEMSKLVNTCSDLHVKNLREEVFNIIKTPFDFARKLFKSLLIFPGTKWCGAGDVADDYDDLGIYEKTDKCCRTHDHCNDSIVGFETKYDLKNKDFYTKSSCSCDLRFHSCLYKKEAIHSDAVGHLFFNILQTQCFKDEYPIVKCLKKWGIPLIRETCQKYKLDYNGTKKYQFFDAKMYKGKNESPFLKKLLSH* | |
| Hconcolorous\|003898 | PAGKTKYGLKNEGTYTMMNCKCEEAFDKCLSDIPGYFTQKAVSMVRYTYFELYGN | |
| Hconcolorous\|004391 | **MAFLCLTSLLILISLRNA***RIIQRELYVNFEPVSNQKDSWPVARAAIVNFDEGFETGR*EFSECRMLNSIQEIARETVNFPQHTIKRVSKEEMDVLERTCSRPLETERFFIYKGTKWCGPGNIAENEFDLGILQADKCCYAHDHCDSIAAGETKYGLVNNGYYTLLNCDCEESFDRCLKATADKVEGSEKEDTLKIRHIYFNTIKSKCYRLYCRNRRSGTDNTCINKTALWKESYHEF* | |
| Hconcolorous\|005385 | **MVKKKCLVSLVICFALTTPPSSS***KDGETLIVNQLSIPQDTHSPTTNSRNKHSEGHKGAKKGVDLASNRPSRIRR*SIMQLAGMLRCTTGCDPLAYKSYGCYCGYLGAGRPVDDIDSCCREHDRCYSNTICPQLFLYFFRYPWRCTSLGIPQCGRMPGITIAQQCARQLCLCDREFVRCIGRFPCPRQSAICRTNPLGVLQSLIVGKR* | |
| Hconcolorous\|007421 | MKRQKSALFEMMNLSLSGCGFLCIYHVGVASCFREYAPHVLVDKIAGASGGSLAACALICSVPLGETTSDVLKIALQARSRTLGPLHPGFDLNKILHEGLVRMLPEDAHLRCNGRLHISVTRVKDFKNVLLSEFNSRDDLIQALLCSCFIPFYSGIVPPKFCGVAYVDGGFSDNLPVLDDHTITVSPFAGESDICPQDTSFNILQFSMSNTSISISAGNLYRFVSTLFPPHPEVLSQMCQQGFDDALKFLQRNNIISCTRCLAVQSSFGIAESGITQQTDTKEIDHPDDDCIDCRYRRQMALLDSLPEAVVKAIEDCCDQMNKGVINWLFRHNPVKILPFFTLPYVLPIDITIVIFAKIWETLPYVQREMKSSLSEFLTFIRNLITSFDKKSQYSAKFSCQLAITEFDYANKEKKSTVAPVIKVLQSSPDGESTAAKRQLKKRMSYAGCANISRQLPMRRKSMVETSSPERVIKNMKVDFTVDLSETNMVSENKKKKKVIDAFQSLKENDDTNVFDIANKVLELEKDYIEYIEPQKSDFVEALEVTNTNEAVMAYFYKEGKKVKVTEIFNISEEDSSIAMTDDEKELNTNLQWDSDWDLVSSSLPDYVPTIEDEQDLFPLEEYTGPTTSAFGTVDAHGLDTSGNEQRRSRKKSVISKLPFTCMEK* | |
| Hconcolorous\|007422 | MKRQKSALFEMMNLSLSGCGFLCIYHVGVASCFREYAPHVLVDKIAGASGGSLAACALICSVPLGETTSDVLKIALQARSRTLGPLHPGFDLNKILHEGLVRMLPEDAHLRCNGRLHISVTRVKDFKNVLLSEFNSRDDLIQALLCSCFIPFYSGIVPPKFCGVAYVDGGFSDNLPVLDDHTITVSPFAGESDICPQDTSFNILQFSMSNTSISISAGNLYRFVSTLFPPHPEVLSQMCQQGFDDALKFLQRNNIISCTRCLAVQSSFGIAESGITQQTDTKEIDHPDDDCIDCRYRRQMALLDSLPEAVVKAIEDCCDQMNKGVINWLFRHNPVKILPFFTLPYVLPIDITIVIFAKIWETLPYVQREMKSSLSEFLTFIRNLITSFDKKSQYSAKFSCQLAITEFDYANKEKKSTVAPVIKVLQSSPDGESTAAKRQLKKRMSYAGCANISRQLPMRRKSMVETSSPERVIKNMKVDFTVDLSETNMVSF* | |
| Hconcolorous\|009441 | **MLLLKLTVFLLLFVQPAPS***TDLPLPLPHEKKLTAFYQEEQNPYMLIIGR*TGKVIHCHQYEDKKEADRVLAALHLDDVEKIEHRRMESLTKFCAKEKHKFIPKTYTKKVFIYPGTKWCGMGDNAANEDELGKEKEADSCCRDHDHCKDSIPAFSIKHNLTNYSPFTKSHCDCDKQFHTCLAKARTKTATIISNLYFNLLNMECFEHKTCSSNETCITWQWKLAKSDGFAIQ* | |
| Hconcolorous\|010247 | **MNCKLLMLTMLAALSGCHQ***TNDFVSWTVLEDNEKEMR*VRIGHIVALQTSVDWETGLNSTHNGLVLRQVTDGKHLIQLIYDSSWTLLDCEYLKHPEYVKKFLNKFVRDFECVRILNSGQRSPDCNNSTYRLLKDGVPPDLRDLLNYRSLRSDCRQLHIAIRKEVKRRRQIDYDRYKRDLFLFPGTNWCGFGNSAKKFNELGYNAATDRCCRDHDLCPYTVESFSHNYGFFNYRFHTISHCDCDERFRACLKLANTAISNLVGKLYFNIVQTKCFVFKTEEVCLKRSWWGKCMKSKRQKNAYPRDCLSY* | |
| Hconcolorous\|014187 | **MSSIILLVGLLSLVNLALS***TENELYLNFEPLTSQRDGWPVARAVRVQFSNRFEEGRESRR*MEGCQILESLNDIAREALHTPRHAMKRISKEEMEFFEGRCLSVGESERTIWGTKWCGAGNEAANYSDLGLFDNVDRCCREHDHCDNIPAGKTKYGLKNEGVFTMMNCKCEEKFGKCLDDIDGIISSKPVSAVKYVYFELYGNGCYNVKCENGRSTSGECANGVAEYTGENGVVAKFINLFGD* | |
| Hconcolorous\|019239 | **MNFWCTLVLVTLLTVCCRINECRS***LHRKPRSLPQLARMVEATTGR*KATDFVPYGNWCGIGGSGKVVDPVDKCCQTHDNCYTRASENGICKAIVSLYIGKYTWKFEKGEIICSTPEDSNECDAASCICDKEVAMCLAKNIGSYQKDHRFVRSLFKS* | |
| Hconcolorous\|021750 | MTASPQPQIKTVHQLQNMLALFWKSVNSNYNKMPSSYVTGLFKTKQWKNAVKNCKISEFAKIVGIMQFHWKFPILLQSNINKNTENLPILKSDTQHAPVTKKTEPTQILELDKALTDWINDDKKPDNNLSFQDNRNSLDIAKSKPTSSVLGNLEKVLSSWFQGDALTFGFQTSKNWMMAQKDESSLTTTYKVTKINVESRSRFLVKSLSSASSETSILYRLQEMGKHLLRYPDQKDIMVKENAIPVTLRIRESFSNTIQAKAQEILSLLGYSDPPGSAGVRILSIDGGGIRGMIAIEILRYLENVSGKPVYQLFDYICGVSTGAILSMLVGGLHLPLDECERLYVKMSSELFKRSAFLGTSGLIWSQSYYDTSMWVNMLRKTYGEIMLSETCNKKGNPKISCVSAVMNQPILEAYAFRNYSLPYRKQSLYPGSSNHKLWQAIRASAAAPGYFEQYTLAGIVHQDGGLIMNNPTPLAVHEAKLLWPKENIQCVLSVGNGRFIPSPEKMAASAGLTTVMMKVIDSATDTESFHAVLQDHLPSNVYFRINPYMTERVSLDEIRTEKLNLLKQDAKKYISWNEDKIQQAVQALTKPRSHLQKFGDWIKLWSLKHQKKSL* | |
| Hconcolorous\|023760 | *MNFLEIGPLSFINDLGEKGREGLVLKR*SGGHFTKPVCLKLRRAVTDCCGFWRKRWLVSKDNFVAYIRPKDGIIKSVLLMDSAFTVDCGLTATGVHHGLLISNMCRQLLVKCWTRRKAREWMQHMTETANTLARDYTQQNRHGSFAPVRDSVEARWFIDGGTYFEAVADALEKAKVEIFIADWWLSPEIYLKRPVIQGELWRLDRVLKRKAEEGVKIFVLLYKEVELALGINSYYSKRQLAQLHPNIKVLRHPDHVTGGVLLWAHHEKIVVVDQIYAFLGGIDLCYGRWDDYLHRLTDLGGIFKTSQNTKTSEYQLLPRRCCSTSDLSEVSHLENCQKIIIQAKQMPKIEIHKHHSAASLPASHDTGIAISDDSQQPETNVESRHVPSTDLLYTIEDERIISKKDKDTSDGIMPSRPRFTSKLRTQRVMQAVTRFQALRHKLQHKDYSQDSLKMGALSDNLGLPTTELRRTASEIALHQMGLEGSCKLWFGKDYSNFIMKDFVHLHQPYQDLVDRTTTPRMPWHDIGILVQGLAAKDVARHFIQRWNFTKMEKAKSYGSYPWLIPKSTGSFTHISPLPKSSTGVIFCTNCQILRSVSTWSSGIRNTEKSIHAAYIDAIKNAKHFIYIENQFFITQAAGNKDVFNEIGEALYQRIIQAYKNNEMFRVYVVMPLLPAFEGEIGTNTGTAIQAITHWNYASICRGGSSLLHRLAQEIEDPVSYISFYGLRNYSMLNDK | |
| Hconcolorous\|023761 | *MSASGGGFAVTHPSEADSDYEDLRPPDSEDDEIDDGAHKDGQPYIPYSKIHETPFGFEDLRQQLLVPNYPINLRIVDAQRLFGTSVMNPNLYIIKLQHGNFEWTIKKRYKHFQRLHQQLLLFRASLSFPLPGRRYRERRKSYRSKDKKSLPQFPRRPEPLIQQEHLPHRAKQLEEYLRKLLRIPLYRSHYETMNFLEIGPLSFINDLGEKGREGLVLKR*SGGHFTKPVCLKLRRAVTDCCGFWRKRWLVSKDNFVAYIRPKDGIIKSVLLMDSAFTVDCGLTATGVHHGLLISNMCRQLLVKCWTRRKAREWMQHMTETANTLARDYTQQNRHGSFAPVRDSVEARWFIDGGTYFEAVADALEKAKVEIFIADWWLSPEIYLKRPVIQGELWRLDRVLKRKAEEGVKIFVLLYKEVELALGINSYYSKRQLAQLHPNIKVLRHPDHVTGGVLLWAHHEKIVVVDQIYAFLGGIDLCYGRWDDYLHRLTDLGGIFKTSQNTKTSEYQLLPRRCCSTSDLSEVSHLENCQKIIIQAKQMPKIEIHKHHSAASLPASHDTGIAISDDSQQPETNVESRHVPSTDLLYTIEDERIISKKDKDTSDGIMPSRPRFTSKLRTQRVMQAVTRFQALRHKLQHKDYSQDSLKMGALSDNLGLPTTELRRTASEIALHQMGLEGSCKLWFGKDYSNFIMKDFVHLHQPYQDLVDRTTTPRMPWHDIGILVQGLAAKDVARHFIQRWNFTKMEKAKSYGSYPWLIPKSTGSFTHISPLPKSSTGVIFCTNCQILRSVSTWSSGIRNTEKSIHAAYIDAIKNAKHFIYIENQFFITQAAGNKDVFNEIGEALYQRIIQAYKNNEMFRVYVVMPLLPAFEGEIGTNTGTAIQAITHWNYASICRGGSSLLHRLAQEIEDPVSYISFYGLRNYSMLNDK | |
| Hconcolorous\|023762 | *MLQVTELVYVHSKLMIVDDRIVIIGSANINDRSMIGKRDSEIAAVVEDVDFEKSLMNEIPYDAGAFAGSLRR*SLFREHLGLMSKDHESCDIRDPISEHFFKEVWMKTAETNTAIYEKVFRCIPTDNVHTFSKLRQYLSQSVMAKTEPEIASKFLQQVKGYLVLLPLNFLCSENLTPAAGTKEALMPVSLWT* | |
| Hconcolorous\|023763 | *PLSDKASLASDLVDRTTTPRMPWHDIGILVQGLAAKDVA*HFIQRWNFTKMEKAKSYGSYPWLIPKSTGSFTHISPLPKSSTGVIFCTNCQILRSVSTWSSGIRNTEKSIHAAYIDAIKNAKHFIYIENQFFITQAAGNKDVFNEIGEALYQRIIQAYKNNEMFRVYVVMPLLPAFEGEIGTNTGTAIQAITHWNYASICRGGSSLLHRLAQEIEDPVSYISFYGLRNYSMLNDKLVTELVYVHSKLMIVDDRIVIIGSANINDRSMIGKRDSEIAAVVEDVDFEKSLMNEIPYDAGAFAGSLRRSLFREHLGLMSKDHESCDIRDPISEHFFKEVWMKTAETNTAIYEKVFRCIPTDNVHTFSKLRQYLSQSVMAKTEPEIASKFLQQVKGYLVLLPLNFLCSENLTPAAGTKEALMPVSLWT* | |
| Hconcolorous\|026891 | MLKKTELTKRKWNGWIKPSCIPITIIFILIVLVVMLPLLDQKHDDKVHLLTEPSNCTEPCWATLVESIPENMTYNISLNHPSTFEGWLNLIQLAEHTIDIAAFYWTLRGGDVISDPSDWQGEKIFDDLLAAGTERKIKISIVQNLASHRFPNNDTKELEEKGAAAVRNINFKKLVGKGILHTKMLIVDDKHFYVGSANMDWRALTQVKELGIIVYNCSCLAKDVKKIFDAYWLLSETEHIPLPWPPYFDTTFNKTHPAVLTINNTSTYAYSSSSPPQFCAKGRTSDINSILSVIHKAKEFIHVAVMDYFPALIYTKHLKYWPVIDNALREAAITRRIKVKVLASYWKNTRPVMQPFLRSLQSLNSTDISIETKLFVVPIYSPSQAKIPYARVNHNKYMVTDNSAYIGTSNWSGDYFISTGGVGFILENTDNSTNSSIQIQLQDIFERDWFSEYAYPITEIDIR* | |
| Hconcolorous\|027415 | **MNILFIAVFLNIITNFDVKS**QSILSGVQKALDSVSNFIDDISFGIKQVRDGLSTVDDIVNYAQGKPCEYKCPPGLKLKKNYYYKPIPQGCGAYGIQVVLSLPLLKDTEKCCDKHDICYSTCMTNKTHCDAEFGTCLYKKCEKQAKKLGEDVRKCTGVSKLFHLGVQSLGCNAFKAAQAEACRCASKDEL* | |
| Hconcolorous\|032907 | *DIISKAKR*YIHISVMDYMPTTCHNQNLRYWSILDGALRQAAIDRRVSIKILVSDWNETPPSMFYFLKSLLALNSTLIHIDVKLFIMP | |
| Hconcolorous\|036712 | NERGIRVRIVRNHPFTSSPRPYGDEFLDSENVQTQNINLRKLQGAGLVPGTQIWIVDNTHLYIGSSALDWMMVNQMKDIGIAMYNC | |
| Hconcolorous\|036713 | AMYNCSCVAEDLQKIFQLYWMLTGTDSSIPKDWPITLETTINKEVPLNMEINDTYTQLYISNSPEILCPEGRSSNIDSILDIISKAKQIGR | |
| **Hyaluronidases** | | |
| Hconcolorous\|007008 | MLWLVRTIAVIIFIFGGKANFNVYWNVPSALCSKKYGINVTDDLMKHRILVNNGEQFIGDKIVIFYENKLGKYPYIDTKKGIYVNGGIPQLANLTEHLKAAEKDIDNMIPDPNFDGIGIIDWESWSPIYDYNWNKKNIYRTRSIELVKKITPPFPAETIKRIAKKQWEEIAKQWMLQTLELAKKKRPKARWCYYLFPDCYNYKEKQTQSEFACRESVRTNNDRLSWLWEASTALCPSIYFYNKHVNYSYHQRLWYLHGRLSEAVRVSSGKALIYPFLNYLCKDKDKNILFDVPEKQFWQMVSFTASMSLDGVVIWGSSSYVKRKENCEALASSVKKVIGPSSLTVSSNFNHCSVTICSGAGRCFWPDKQFISLTYMSKDPPRLQPEEIFCRCKKNLGRYCNVSNLDPHHFIEDCSAEVDQQ* | |
| Hconcolorous\|007009 | *MLMEEYRRQWMLQTLELAKKKRPKAR*WCYYLFPDCYNYKEKQTQSEFACRESVRTNNDRLSWLWEASTALCPSIYFYNKHVNYSYHQRLWYLHGRLSEAVRVSSGKALIYPFLNYLCKDKDKNILFDVPEKQFWQMVSFTASMSLDGVVIWGSSSYVKRKENCEALASSVKKVIGPSSLTVSSNFNHCSVTICSGAGRCFWPDKQFISLTYMSKDPPRLQPEEIFCRCKKNLGRYCNVSNLDPHHFIEDCSAEVDQQ* | |
| Hconcolorous\|021774 | **MLFFVCIVSIFNNIEA**SFDVFWNVPSSLCSIKYDVNMTETLLKYNILVNDGESFTGDKITLIYENGIGKYPHIDPNKGDINGGLPRLDKLKEHLNLAEKDIQKIIPNPAFTGLGIIDWEAWRPIWEYHWGGLSIYQKRTIDLVKKDHPTESDQFIQTTAKNLWENTAKQWMLKTLELAKKLRPQGQWCYYLFPDCYNYFGKDQPSEYFCSAMIQNNNDRLSWLWDASTALCPSIYFIENQMKYNESQRTWFLYGKLAEAARVARPSTKIYPYINYMVHVSQIPVPRDHFWKMLSLIASMGFDGAVIWGSSSYLGSKKSCDDLEAYIENVIGPAVTTISSNVNRCAQEICNGRGRCTWPNEPFISWQYLIDTNGPNFDSQKITCKCQSHSGRYCN* | |
| Hconcolorous\|030771 | **MNSYILIGIILNFIAHVIEA**SFKVYWNVPSSLCSVKFGINVTETLINHDVLVNYDEHFLGEKLVIFYENKLGKYPYIDPKYGDVNGGLPQLGNLEKHLEAAEKDIQTIISNPNFDGLGIIDWEKWRPIWDFNWGKMRIYKTRTMELMQKKHPSWPWKLIENASRNQWEETAKQWMLKTLELTKKIRPEGRWCFYHLPDCYNYAGKDKPEQFLCSSLVRKHNDRLIWLWNATTALCPSIYFDERQTKYNESQQIWFLYGRLSEVLRVSQPRTPIYPYINYRIHSSLEEVPKERFWLMLGHLASLGLDGAVIWGSSNYVKTEEECRNLELYVKEVIGPASSTISSNVNRCSEVICNGEGMCTWPHQSYTSWKYLTNQNASAFKREDITCRCQMYKGRYCDLYHSNSTDLF* | |
| Hconcolorous\|033175 | IPDPNFDGLGVIDWESWRPIYNYNWGTMTIYKTRTVELVRKENPSLKVDLIQSIAEKQWEEIA | |
| Hconcolorous\|033907 | GGNCKEHFWRMLSLSASMGLDGIVIWGSSNYVRKKEDCEALASYVKKVIGPSSLTVSTNFNFCSKTICRGQGRCVWPDEPYTSWRYMCNRDLSDFQPGEIICRCKKKERQIL* | |
| **Metalloproteases** | | |
| Hconcolorous\|004160 | **MAVRSMCPFTTLSLLLLSFGSKQSVS***QDRR*PNTGLVFPEANTQYCRTRDGSAGSCVQISECKHDIDYQRGILPELCYWDNSRPIVCCLRNDRTETPVTVPDRDRVTSVTGCGKRTIPKDASRSPQIAGGRISLPSAWPWMISIHRSNFGIESFLCGGTMVSVRYILTAAHCFGRNGNDRRKIPTSRFVIRVGSNINEEGVAHRIKNIIVHEDYKVGQHYNDLAVIEVTELIKLSPMVQPICLPSSEMQGRQLVGRDVTVIGWGDQSFGGIRDRKLREVNISVIDRQQCDESYSALSSLAIPRGITSQFLCAGDVKGGKDACQADSGGPLMMHSSDWTIVGIVSFGYGCAQKGYPGVYTQVVSYLQWIKDNTRM* | |
| Hconcolorous\|006453 | IISLRKLPRSSESSTCQSHICKKTGEKLKSWINSNIDPCEDFYNHCCGGWLKENPLPNDTEIYSVFNKLESEISEYIKQLLEESNYKQSPRFINQTLMFYKACLHKENIEAGKAKSLLSFLEELGGWPLLTNDWKGEDYDWVEVISKLVWKTASGYLIRFIISPDVKNTSNTIIQLDRPSLMMNADELLNPNGTSD | |
| Hconcolorous\|012106 | *IVKFITAR*PMGDKQEGSNKANPGVVGIAYVGGVCDRDYKCGVSEDNGLDFDGSSTYAHEVAHLVGCPHDGDPPVSYLPGSPGAKSCSWDLGYIMSYVRKNEKQFAFSSCSEECVSHLATLSGSECLFRQDVTNSLNNQENRLPGDFFSDTIGTTDMKSIYEGKCKIKRKDFTLYKIPNNSCDYSCKTPTVNNRFYYWIFQCSDGDICDTNKVCING | |
| Hconcolorous\|018739 | *MGNISSSQRLALNVERTSSKR*LYFPIWPGGRIPYEIDQQSVCPIPNIRDAMREIEFYTKCIQFVPKRNEVDYLYITTSKNQISSKGCSSQYVGRLGGKQTLILNLECCDRLIYLHELCHAIGLHHEHQRWIRDHYITIDFCNIIPGHHHNFDKLPPDDLGDFDFDSIMMYEPYAFSLDECKPVIKPLDQKMRIREMREKTSLSRGDICKIRRLYK* | |
| Hconcolorous\|018740 | *MGNISSSQRLALNVERTSSKR*LYFPIWPGGRIPYEIDQQSVCPIPNIRDAMREIEFYTKCIQFVPKRNEVDYLYITTSKNQISKGCSSQYVGRLGGKQTLILNLECCDRLIYLHELCHAIGLHHEHQRWIRDHYITIDFCNIIPGHHHNFDKLPPDDLGDFDFDSIMMYEPYAFSLDECKPVIKPLDQKMRIREMREKTSLSRGDICKIRRLYK* | |
| Hconcolorous\|019720 | **MLWYRCFLFLLAHFNCTVWA***LHKLRDAEAQEAIPILTDSRGQPLPIER*FDLYSKLFFKFVAFNFTFFIELYPANEFISPTFHVNNIETVGNSTNSRMQQHKSFKNCYFRGRIVDEVNSLVSVSLCQGMLGFFRTSRGDYMIEPSIGRLSSVHFIKSVPVPNGNKTTQICEARDSFHDTMLNRAMHRHPPGRRRKRSVSVERNLEVMVVADSKMARYHKSNLRHYILTLMSTVALIYKDPSIGNDINIVVVKLVVMDESEDIDIIFPSASKTLRNFCRWQQQYNDYDDSSPHHHDTAVLLTREDLCRLAKTCDTLGLAQSGMVCNHKSSCAIVEDNGLSAAFTIAHELGHVLSIPHDDDHKCNRFQGDGQRLHVMARMLDYNSHPWSWSPCSRHYITAFLDSGKGHCLLDKPITNEIQSPEHETHQPGQLYDMDHQCELVFGKGSKICPYMPVCKRLWCTMEDLTQGGCRTQHMPWADGTLCGREKWCQHGECIRMRAATQYPVDGQWGKWQSFGQCSRSCGGGVKRSERYCDNPSPAGGGKYCVGKRVRYKSCSSQECPNGIQDFREEQCSAFNGNSDFLDSRTNVKWTPQYDGIHMDERCKLYCRAVGTSAYFPLKDKVIDGTPCGVDTFDMCVNGKCLPAGCDHRLDSKKKLDICGICGGDNSTCKIVAGHFNKVEYGYNHVVLIPAGASNLDIRQFGYQNSNDDNYLALKDSSGRYLLNGDFMVSMFPKTIQYGGAMLEYSGSDKIVERINTTKQLKKDLIIMVLTVGKLRSPDVRFQYTVSLENAQFYEWDIEKKWSTCSHACRGEQYRDPICVDIYSRRVVNDAHCYSNQKPPRLAQVCNLQCSLSWQILKKSDCSSNCGKGTRHRIFKCVQQSGEDSQLLEDSFCEHLESKPQEIEECEGPCSNIQWEFGEWSECSQTCGEGIQTRSAICIDFQNEKQNDALCDASAKITTKSCNLSICPEWEVGDWTQCSVTCGIGERHRPYWCRYADQVVSVTFCNRETVPIHKEKCNMGRCLQWNYGNWGPCSKSCGQGYSKRVVHCQLTNGTVVDDDMCDVDTKPTSAKECNLPSCPTTATPSSTFYTTDTTDSNFLPPIPLALVQDDMPKSATEKTVHTSTTDMPETAKWKSGSWSECSVTCGRGTRKRHLVCHDQVKHQIVSDSECDILLQPPILEVCEVQDCGQWEIGDWSQCPVTCGEGLSIRTVVCIKLNGEKGENHECDQSTRPSTERLCSMKPCEILPIGVPEMPIISHNSVEGIFYWRTGHWGECSKTCGEGKKRRQVACYDEAGHISSQCSSHQKPDEISTCNREPCPDWVVSDWSVCSHTCGGGIQIRSVHCQSSLRTFADEYCDSSMKPFTRQECNSQQCTINLNQYRWEKEEWSQCNVSCGTGFMHRHVYCIDSQHFVVSDKRCTTKRPRVRRKCKGTRCHYVWKTEDWSTCSEVCGKGTMKRKISCHQLNLYGWMDPTPIPQPLGNREHWCDVNNRPSDTKSCNLGKCEKNASWKVGPWNRCSHDCGKGKQRRRVQCFNLQGRKISFRKCERDLRPRRKRNCFIRSCAPISCSELYHRNRIRENGEYKLQVRGRPVQIYCAGMNTTSHRHYISLVSGEGDNYSEIYDKRLVKPETCPHGGARQENCNNECVDAQEGAGLTTFFRIGINLTSLQVLTHDFAFSQTHHGQQVPYGESGDCYSKKNCPQGRFSINLMGTGFVVSQKTTWVKQGNEPDAKVQRLKHGQVVQGKCGGYCGKCGPDSNVGLLLDIAPP* | |
| Hconcolorous\|023528 | **MRWYQVIVVISTLFVQHVVWT***ESRTVSGEDPNEMEGEIVYPRVYHMSRKKRDVGSSDETR*LIVIKADNTTFYLELKPNDNLVIDEIDYNDTSDPCIFQGKILSHPGGMAAISTCEGGGNMNGLLITPEESLLLQPLSKFKPHHVLHPIEKDIVAHVLFKAEDGVEEFCGTDHLGNNTYHLVPEEENEIPKENSRVKRSEKAVYTIETAVFVDKYLYNRYSEHSPRRTSQYAREMVFTIMNQVQLIYKYKSMKTNINIVIVKLEILPNSFQESVQSSEGDIDVYLDEWCTWQGKRTDRVWDHAVLLTGLDLFKMQNGSKNKKVLGLAWVNGMCRPKYSCTLNEGMNFEAAFVIAHEMGHCLAMLHDGTGNTCDDNTYIMSPKTGPGKTHWSSCSNKYLADFIKSGYAPCLEDGGKPVSSDLDLQNPPSLPGERISVNEQCKLALGPEYKVYEKSSSPYNDICRELWCVKGLWATSAHPALEGSKCGRNKKCLEGQCQGRTMKQTNQARYSSNMIQTATLFQQMLDKMRMLSREFLRFLHLNYT* | |
| Hconcolorous\|023662 | **MIAKCLILFHLMDQLVC***LSLSELTFDKKQGDYVNGLKHYEVIVPQKVNENGLLIDNDLTHYYNFRSKRSTRISPLHYRLITTEHDMFLSLHPNHAAVSPGYVVER*YLGIDNGTIRLTSKELKNKHCYYRGDVRNLSGSSVALSTCNGLMGVIRVKGEDYFIEPVKGHPLNGTSRHLHLLYKRSAVSNGPYEYLPKEGKCGNKDDIGQAITKRAQWEKKRESGRRKKRKKRSVSLERNVETLVVADRKMVEYYLDDDIETYILTVMNVVSSLYHDASIGNAVNIIVVRLILLENEENKKEDPEISHDADNTLKSFCKWQRYINYKDETHPNHHDVAILLTRYNICTKINEPCSTLGLAEVAGMCQPHRSCNVNEDTGLGLAYTIAHEMGHNFGMSHDGPHNGCQALLGERQHVMSPHLNSDASPFIWSNCSRLEITKFLDRDWGSCLDDEPSDLNFNIPELPPGTMYNADHQCRLQYGPDAEFCEGIEDICQTLWCRQDNRCVTRLEPAADGTLCDRNMWCYMGKCVPVGERPTSINGEWGPWSSWTECSRSCDAGVMHSERHCNNPMPANGGRFCIGERKRYRICKSEPCLDDALSFRSVQCSKFDSIPYKEELHTWLPISTPLTPCQLHCKPKGKFFSVMLSDTVEDGTPCNPGTNDMCINGKCRKVACDWIIDSTAQEDRCGICHGDGTLCKTIRGKFLQRRGIGYIEIVKVPKGARNIRVEELGDATNYIAIQDQHGEFQLNGQWFIQWSGEYIAAGTIFYYHREGEKEELHAPGPTKEVVRILLLFQTENPGLTYDYTIPNKNVTRKPEFHWEYTDWSVCSSTCNGGIQVSRAKCFEKEAGLVENSYCNESIKPPDRTQICNRHECPARWWSGPWQHCSVSCGDNGIRRRTVICIRSLGPEEQVALPDEYCDMNIRPPPQQSCHHTHPCLMDARWETGNWTDNCDEDPCSYQTRHVYCNIPNGHCHEKDKPVSIRQCNNITCGVWTVGNWSECSQSCGEGHQQRDITCVGGSACHRATQPPHTQICNPTRCSSLSDFQISDNTIEETHKDSDIKVSIHKHRHHHGDNKNKNSKEISQRSHDVAKDIVAYPIYEVPDNKYKDFDIDVIHPIKKEPIIVAQHNNHHTELEFRKYEWKVGMWSECSAPCGGGIIKREVLCFDTITGHMVVTDLCDPFQMPNNEDSCNMDLCAEWIHSEWNECSSSCGQGWQHREVYCPRKYKCNYHSKPIESRPCSVKPCEQWIAGPWSQCSVTCGEGYQTRHVKCVNLKTQALVMDCSNEDRPKHYQVCRNEECREEKSEFNQCYDKLEVSTCRSLPHMCNTWYFKAKCCQTCNRLEISRRRIRKSKDET* | |
| Hconcolorous\|029707 | MVMVWNEGIIRYTFDDKLSDSIQEMVTFAMRKITNCTNCIKFFYRKHARDYLFITEGERCSSHVGRLGGKQELYLDTNHIALSVILHELCHAIGLYHEHQRSIRDDYIKVYYENIKEEHRMHFIRMSREDDIEGFGFDFYSIMLYGNH | |
| **HDPs** | | |
| Hconcolorous\|004367 | **MNAKILLVVFMITMFVTEQVEG***KFSFSWIKR*IWKSKIGKAARKAAGNYVAKKLENAAAPAEGGPKRFDEFMDSLYY* | |
| Hconcolorous\|021934 | **MNAKVFLAIFMIALLVTDQAEA**GWWKALKSIGKKVWKSKLAKDIKNMAKQRAKEYIVKKLNPPPEEEVAAIDALMNSLDY* | |
| Hconcolorous\|027436 | *LTPFLFR*SIVNLQPSVHKKMNAKVFLVVFMIALFVTEKAEAGILDTIKSIASKVWNSKTVQDLKRKGVNWIANKLGVSPQVAASMTLDEIMDAFENY* | |
| **Other inhibitors** | | |
| Hconcolorous\|002969 | **MKTALIYIVILTFVVATFA**QKKTECQESREKALKSNARIKAIVPVCDENGDYAALQCHEGSKFCSCWRKDGTPITQPSGKIKACECHRQKDEKSSKGLLGAFIPQCAEDGKFQKIQCWSSTGYCWCADPDTGRNTTASARGTLNC* | |
| Hconcolorous\|009579 | **MKILLGLFVLTVTVSC**QPQSGGIGCKPYEEYREAWCEVTCDNVRRVTCLTAEKRPGCYCKIGTIRDEDGQCISTEACSKRVCTKKNQRLDMSGCFTVCTGLGTSYFGCPFVQQPSCNCKRGFAVARGIRGDCIPVSSCRKPNWGN* | |
| Hconcolorous\|010515 | **MKLFILVCFMLVLVTLSLA**EQTPCQEKREKILSQNLDVEVIPECEENGSYKAKQCKKNGVDCQCWRTDGTPINDFSPNLKACSCIRSKDNANRPHLIGNYKPQCEADGTYSLTQCWGSVGGCWCVDAEGRKLPNKHFPVDC* | |
| Hconcolorous\|010516 | **MISKCLRSDKIKMKLFILVCFMLVLVTLSLA**EQTPCQEKREKILSQNLDVEVIPECEENGSYKAKQCKKNGVDCQCWRTDGTPINDFSPNLKACSCIRSKDNANRPHLIGNYKPQCEADGTYSLTQCWGSVGGCWCVDAEGRKLPNKHFPVDC* | |
| **Other venom components** | | |
| Hconcolorous\|002969 | **MKTALIYIVILTFVVATFA**QKKTECQESREKALKSNARIKAIVPVCDENGDYAALQCHEGSKFCSCWRKDGTPITQPSGKIKACECHRQKDEKSSKGLLGAFIPQCAEDGKFQKIQCWSSTGYCWCADPDTGRNTTASARGTLNC* | |
| Hconcolorous\|002972 | **MKTALIYIVILTFVVATFA**QKKTECQESREKALKSNARIKAIVPVCDENGDYAALQCHEGSKFCSCWRKDGTPITQPSGKIKACECHRQKDEKSSKGLLGAFIPQCAEDGKFQKIQCWSSTGYCWCADPDTGRNTTASARGTLNC* | |
| Hconcolorous\|009196 | **MFRLILFFISVASVYS**LSCPCWREPDKTKYCPPPPTNCPLGLTTGPCGCCLQCYKDNGEACGGPWRIIGKCGKGLRCVKETNVGEPKRYYINQMEGVCKPIDTY* | |
| Hconcolorous\|010515 | **MKLFILVCFMLVLVTLSLA**EQTPCQEKREKILSQNLDVEVIPECEENGSYKAKQCKKNGVDCQCWRTDGTPINDFSPNLKACSCIRSKDNANRPHLIGNYKPQCEADGTYSLTQCWGSVGGCWCVDAEGRKLPNKHFPVDC* | |
| Hconcolorous\|010516 | **MISKCLRSDKIKMKLFILVCFMLVLVTLSLA**EQTPCQEKREKILSQNLDVEVIPECEENGSYKAKQCKKNGVDCQCWRTDGTPINDFSPNLKACSCIRSKDNANRPHLIGNYKPQCEADGTYSLTQCWGSVGGCWCVDAEGRKLPNKHFPVDC* | |
| Hconcolorous\|014939 | **MLRLILLCILVATVYS**LSCPCWYEEDTTKYCPPTPTNCPIGLTLGPCGCCLECYKDKGEVCGGSWQMLGKCGEGLRCEKGFDDLGSDYYYANHKEGVCQPIEPIDLL* | |
| Hconcolorous\|017887 | MSFVIYRFPKLYSYTGVKMRIYVDFSWFQKATHQDMGKKLCLVVFVLLGIFTSAMALTCLACGSYECPPLPLCPAGIVKDVCDCCAVCAKNENENCGGMWEEYGKCGEGLKCVTTGATTPHPFMSDWPIPGICQKE* | |
| Hconcolorous\|017888 | MSFVIYRFPKLYSYTELKMRIYVDFSWFQKATHQDMGKKLCLVVFVLLGIFTSAMALTCLACGSYECPPLPLCPAGIVKDVCDCCAVCAKNENENCGGMWEEYGKCGEGLKCVTTGATTPHPFMSDWPIPGICQKE* | |
| Hconcolorous\|022553 | **MWFRFIVLFLFVAGVYS**LSCPCQTNRLLCKPAPTNCKLGLTKDACGCCDVCYKIEGEECGGPWKTSGNCGKGLKCVIPENLPKHIQQQATGICKVE* | |
| Hconcolorous\|022554 | **MWFRFIVLFLFVAGVYS**LSCPCQRDLSLCEPAPTDCKLGLTKDACGCCDVCYKIEGEECGGPWNTSGNCGKGLKCVIPGNLSKYIELQATGICKVE* | |
| Hconcolorous\|028918 | **MGVKFYFIAFVLNVVFLNTMA**LKCKECGTYECRPPPNNCPVGTVTDVCNCCLVCGKAENEICGGEWNLLGKCGEGLKCVEKPSSNNQFIAPKAGICKKK* | |
| Hconcolorous\|034053 | **MRLYLFAFAVLIASCHC**APRNRCTQECGPVPNNCRAGVTKDYDGCCAVCAKSEGEECGGMWNAYGVCGIDLICNTNGNLISDYDLPIGICISARRIVNRNILKRMLRGLH* | |

**Table S3.** List of the 24-scorpion species used in the phylogenomic analyses.

|  | **Species** | **SRA Accession** | **Reference** |
| --- | --- | --- | --- |
|  |  |  |  |
| **BOTHRIUROIDEA** |  |  |  |
| Bothriuridae | *Bothriurus coriaceaus* | SRR6467511 | [1] |
|  | *Centromachetes* sp. | SRR6467879 | [1] |
|  | *Cercophonius queenslandae* | SRR6467879 | [1] |
|  | *Cercophonius squama* | SRR6470146 | [1] |
|  | *Cercophonius sulcatus* | SRR6470446 | [1] |
| **BUTHOIDEA** |  |  |  |
| Buthidae | *Androctonus australis* | SRR1724216 | [2] |
|  |  |  |  |
| **CHACTOIDEA** |  |  |  |
| Chactidae | *Anuroctonus phaiodactylus* | SRR1721879 | [2] |
| Euscorpiidae | *Megacormus gertschi* | SRR3657526 | [3] |
| **HADRUROIDEA** |  |  |  |
| Hadruridae | *Hadrurus arizonensis* | SRR1721733 | [2] |
|  | *Hadrurus spadix* | SRR4069278 | [4] |
|  | *Hadrurus concolorous* |  | This study |
|  | *Hoffmannihadrurus gertschi* | - | [5] |
|  | *Hoffmannihadrurus aztecus* |  | This study |
| **IUROIDEA** |  |  |  |
| Iuridae | *Iurus dekanum* | SRR1721734 | [2] |
| ***Incertae Sedis*** |  |  |  |
| Troglotayosicidae | *Belisarius xambeui* | SRR1721953 | [2] |
|  | *Uroctonus mordax* | SRR7415024 | [6] |
| **PSEUDOCHACTOIDEA** |  |  |  |
| Pseudochactidae | *Vietbocap lao* | SRR1721740 | [2] |
| **SCORPIONOIDEA** |  |  |  |
| Scorpionidae | *Pandinus imperator* | SRR1721600 | [2] |
| Urodacidae | *Urodacus elongatus* | SRR7885472 | [7] |
|  | *Urodacus manicatus* | SRR870663 | [8] |
|  | *Urodacus planimanus* | - | [2] |
|  | *Urodacus yaschenkoi* | SRR1557168 | [9] |
| **VAEJOVOIDEA** |  |  |  |
| Vaejovidae | *Paravaejovis schwenkmeyeri* | PRJEB25890 | [10] |
| Vaejovidae | *Serradigitus gertschi* | PRJEB27910 | [11] |

**Table S4.** 84 sequences of Scorpine-like Peptides (SLP), plus 23 potassium channel toxins (KTx) isolated from the venom, or deduced from cDNA or transcriptome analyses of 44 scorpion species.

| **Code** | **Toxin** | **Species** | **Family** | **Source** |
| --- | --- | --- | --- | --- |
| A0A059UI30 | SLP Buthida | *Mesobuthus gibbosus* | Buthidae | UniProt |
| A0A088D9S1 | SLP Buthida | *Mesobuthus eupeus* | Buthidae | UniProt |
| A0A088DB53 | SLP Buthida | *Mesobuthus eupeus* | Buthidae | UniProt |
| A0A0C9RFQ9 | SLP Buthida | *Tityus bahiensis* | Buthidae | UniProt |
| A0A0C9S3A8 | Beta KTx | *Tityus bahiensis* | Buthidae | UniProt |
| A0A0K0LBZ4 | SLP Buthida | *Androctonus bicolor* | Buthidae | UniProt |
| A0A0K0LC02 | SLP Buthida | *Androctonus bicolor* | Buthidae | UniProt |
| A0A0K0LC06 | SLP Buthida | *Androctonus bicolor* | Buthidae | UniProt |
| A0A0K0LC11 | Beta KTx | *Androctonus bicolor* | Buthidae | UniProt |
| A0A0K0LC14 | SLP2 | *Androctonus bicolor* | Buthidae | UniProt |
| A0A0K0LCI9 | SLP Buthida | *Androctonus bicolor* | Buthidae | UniProt |
| A0A0K0LCJ0 | Beta KTx | *Androctonus bicolor* | Buthidae | UniProt |
| A9XE59 | SLP Buthida | *Mesobuthus eupeus* | Buthidae | UniProt |
| A9XE60 | SLP Buthida | *Mesobuthus eupeus* | Buthidae | UniProt |
| alpha-KTx 12.5 mgc25969 | Alpha KTx | *Megacormus gertschi* | Euscorpiidae | From transcriptome |
| alpha-KTx 23.1 mgc29973 | Alpha KTx | *Megacormus gertschi* | Euscorpiidae | From transcriptome |
| alpha-KTx 6.6 mgc21622 | Alpha KTx | *Megacormus gertschi* | Euscorpiidae | From transcriptome |
| API81325 | SLP1 | *Hemiscorpius lepturus* | Hemiscorpiidae | UniProt |
| B.coriaceus m.20282 | SLP1 | *Bothriurus coriaceus* | Bothriuridae | From transcriptome |
| B8XH36 | Beta KTx | *Buthus occitanus* | Buthidae | UniProt |
| B8XH40 | SLP Buthida | *Buthus occitanus* | Buthidae | UniProt |
| Be.xambeui m4045 | SLP2 | *Belisarius xambeui* | Troglotayosicidae | From transcriptome |
| C.queenslandae m.1064 | SLP Bothriuridae | *Cercophonius queenslandae* | Bothriuridae | From transcriptome |
| C.queenslandae m.7768 | SLP1 | *Cercophonius queenslandae* | Bothriuridae | From transcriptome |
| C.squama m4800 | SLP Bothriuridae | *Cercophonius squama* | Bothriuridae | From transcriptome |
| C.squama m5048 | SLP1 | *Cercophonius squama* | Bothriuridae | From transcriptome |
| C5J891 | SLP2 | *Opisthacanthus cayaporum* | Hormuridae | UniProt |
| Ctri27 | SLP Buthida | *Chaerilus tricostatus* | Chaerilidae | [12] |
| Ctri9164 | SLP Buthida | *Chaerilus tricostatus* | Chaerilidae | [12] |
| Ctry22350 | SLP Buthida | *Chaerilus tryznai* | Chaerilidae | [12] |
| Ctry44 | SLP Buthida | *Chaerilus tryznai* | Chaerilidae | [12] |
| Ctry51 | Beta KTx | *Chaerilus tryznai* | Chaerilidae | [12] |
| D9U2A7 | SLP Buthida | *Lychas mucronatus* | Buthidae | UniProt |
| E4VP14 | SLP Buthida | *Mesobuthus eupeus* | Buthidae | UniProt |
| E4VP56 | SLP Buthida | *Mesobuthus eupeus* | Buthidae | UniProt |
| E4VP57 | Beta KTx | *Mesobuthus eupeus* | Buthidae | UniProt |
| H.concolorous\|008061 | SLP2 | *Hadrurus concolorous* | Hadruridae | From transcriptome |
| H.concolorous\|029426 | SLP2 | *Hadrurus concolorous* | Hadruridae | From transcriptome |
| H.concolorous\|033550 | SLP1 | *Hadrurus concolorous* | Hadruridae | From transcriptome |
| H.spadix DN50359 | SLP2 | *Hadrurus spadix* | Hadruridae | From transcriptome |
| H2CYP8 | SLP1 | *Pandinoides cavimanus* | Scorpionidae | UniProt |
| H2CYQ1 | Beta KTx | *Pandinoides cavimanus* | Scorpionidae | UniProt |
| Hg scorpine like 2 mgc19218 | SLP2 | *Megacormus gertschi* | Euscorpiidae | From transcriptome |
| Hge-beta-KTx mgc25471 | Beta KTx | *Megacormus gertschi* | Euscorpiidae | From transcriptome |
| Ho.aztecus\|062933 | SLP2 | *Hoffmannihadrurus aztecus* | Hadruridae | From transcriptome |
| Ho.aztecus\|083866 | SLP1 | *Hoffmannihadrurus aztecus* | Hadruridae | From transcriptome |
| Ho.aztecus\|106327 | SLP2 | *Hoffmannihadrurus aztecus* | Hadruridae | From transcriptome |
| Ho.gertschi EL698900.1.p1 | SLP2 | *Hoffmannihadrurus gertschi* | Hadruridae | From transcriptome |
| Ho.gertschi EL698908.1.p1 | SLP1 | *Hoffmannihadrurus gertschi* | Hadruridae | From transcriptome |
| K9LZ65 | Beta KTx | *Tityus stigmurus* | Buthidae | UniProt |
| L0G8Z0 | SLP1 | *Urodacus yaschenkoi* | Urodacidae | UniProt |
| L0GCW2 | SLP2 | *Urodacus yaschenkoi* | Urodacidae | UniProt |
| P.schwenkmeyeri c22486 | SLP2 | *Paravaejovis schwenkmeyeri* | Vaejovidae | From transcriptome |
| P.schwenkmeyeri c22791 | SLP1 | *Paravaejovis schwenkmeyeri* | Vaejovidae | From transcriptome |
| P.schwenkmeyeri c22885 | SLP2 | *Paravaejovis schwenkmeyeri* | Vaejovidae | From transcriptome |
| P0C2F3 | SLP Buthida | *Tityus stigmurus* | Buthidae | UniProt |
| P0C2F4 | SLP1 | *Heterometrus laoticus* | Scorpionidae | UniProt |
| P0C8W4 | Beta KTx | *Tityus stigmurus* | Buthidae | UniProt |
| P0C8W5 | SLP2 | *Hoffmannihadrurus gertschi* | Hadruridae | UniProt |
| P0CI49 | SLP Buthida | *Lychas mucronatus* | Buthidae | UniProt |
| P0DL47 | SLP1 | *Euscorpiops validus* | Scorpiopidae | UniProt |
| P56972 | SLP1 | *Pandinus imperator* | Scorpionidae | UniProt |
| P69939 | SLP Buthida | *Androctonus australis* | Buthidae | UniProt |
| P69940 | SLP Buthida | *Tityus serrulatus* | Buthidae | UniProt |
| P86822 | Beta KTx | *Tityus serrulatus* | Buthidae | UniProt |
| Q0GY40 | SLP1 | *Hoffmannihadrurus gertschi* | Hadruridae | UniProt |
| Q0GY41 | Beta KTx | *Hoffmannihadrurus gertschi* | Hadruridae | UniProt |
| Q0GY42 | Beta KTx | *Tityus costatus* | Buthidae | UniProt |
| Q0GY43 | Beta KTx | *Tityus discrepans* | Buthidae | UniProt |
| Q0GY44 | SLP Buthida | *Tityus discrepans* | Buthidae | UniProt |
| Q0GY45 | Beta KTx | *Tityus trivittatus* | Buthidae | UniProt |
| Q0GY46 | SLP Buthida | *Tityus trivittatus* | Buthidae | UniProt |
| Q5G8A6 | SLP Buthida | *Tityus costatus* | Buthidae | UniProt |
| Q5WQZ7 | SLP1 | *Opistophthalmus carinatus* | Scorpionidae | UniProt |
| Q5WQZ9 | SLP1 | *Opistophthalmus carinatus* | Scorpionidae | UniProt |
| Q5WR01 | SLP1 | *Opistophthalmus carinatus* | Scorpionidae | UniProt |
| Q5WR03 | SLP1 | *Opistophthalmus carinatus* | Scorpionidae | UniProt |
| Q6XLL8 | Alpha KTx | *Opisthacanthus cayaporum* | Hormuridae | UniProt |
| Q9N661 | SLP Buthida | *Mesobuthus martensii* | Buthidae | UniProt |
| Q9NJC6 | Beta KTx | *Mesobuthus martensii* | Buthidae | UniProt |
| S.gertschi c25700 | SLP2 | *Serradigitus gertschi* | Vaejovidae | From transcriptome |
| S.gertschi c26014 | SLP1 | *Serradigitus gertschi* | Vaejovidae | From transcriptome |
| S.gertschi c26395 | SLP2 | *Serradigitus gertschi* | Vaejovidae | From transcriptome |
| Scorpine-like peptide Ev37 mgc22639 | SLP1 | *Megacormus gertschi* | Euscorpiidae | From transcriptome |
| Su.donensis sdc1422 | SLP2 | *Superstitionia donensis* | Superstitioniidae | From transcriptome |
| Su.donensis sdc34997 | SLP1 | *Superstitionia donensis* | Superstitioniidae | From transcriptome |
| T1DEJ8 | SLP2 | *Urodacus manicatus* | Urodacidae | UniProt |
| T1DEK6 | SLP Buthida | *Isometroides vescus* | Buthidae | UniProt |
| T1DMR0 | SLP1 | *Cercophonius squama* | Botrhiuridae | UniProt |
| T1DMR6 | Beta KTx | *Urodacus manicatus* | Urodacidae | UniProt |
| T1DP99 | SLP Bothriuridae | *Cercophonius squama* | Botrhiuridae | UniProt |
| T1E6W5 | SLP Buthida | *Australobuthus xerolimniorum* | Buthidae | UniProt |
| T1E6W8 | SLP Bothriuridae | *Cercophonius squama* | Botrhiuridae | UniProt |
| T1E6X2 | SLP1 | *Urodacus manicatus* | Urodacidae | UniProt |
| T1E6Y1 | Beta KTx | *Isometroides vescus* | Buthidae | UniProt |
| Toxin KTx8 mgc26539 | Alpha KTx | *Megacormus gertschi* | Euscorpiidae | From transcriptome |
| Ur.mordax DN49907 | SLP2 | *Uroctonus mordax* | Hadruridae | From transcriptome |
| V.lao m10170 | SLP2 | *Vietbocap lao* | Pseudochactidae | From transcriptome |
| ViScp1p1 | SLP1 | *Thorellius intrepidus* | Vaejovidae | [13] |
| ViScp1p2 | SLP1 | *Thorellius intrepidus* | Vaejovidae | [13] |
| ViScp1p3 | SLP2 | *Thorellius intrepidus* | Vaejovidae | [13] |
| VmScp1p1 | SLP1 | *Vaejovis mexicanus* | Vaejovidae | [13] |
| VmScp1p2 | SLP2 | *Vaejovis mexicanus* | Vaejovidae | [13] |
| VmScp1p3 | SLP2 | *Vaejovis mexicanus* | Vaejovidae | [13] |
| VpScp1p1 | SLP1 | *Mesomexovis punctatus* | Vaejovidae | [13] |
| VsScp1p1 | SLP1 | *Mesomexovis subcristatus* | Vaejovidae | [13] |
| VsScp1p2 | SLP2 | *Mesomexovis subcristatus* | Vaejovidae | [13] |


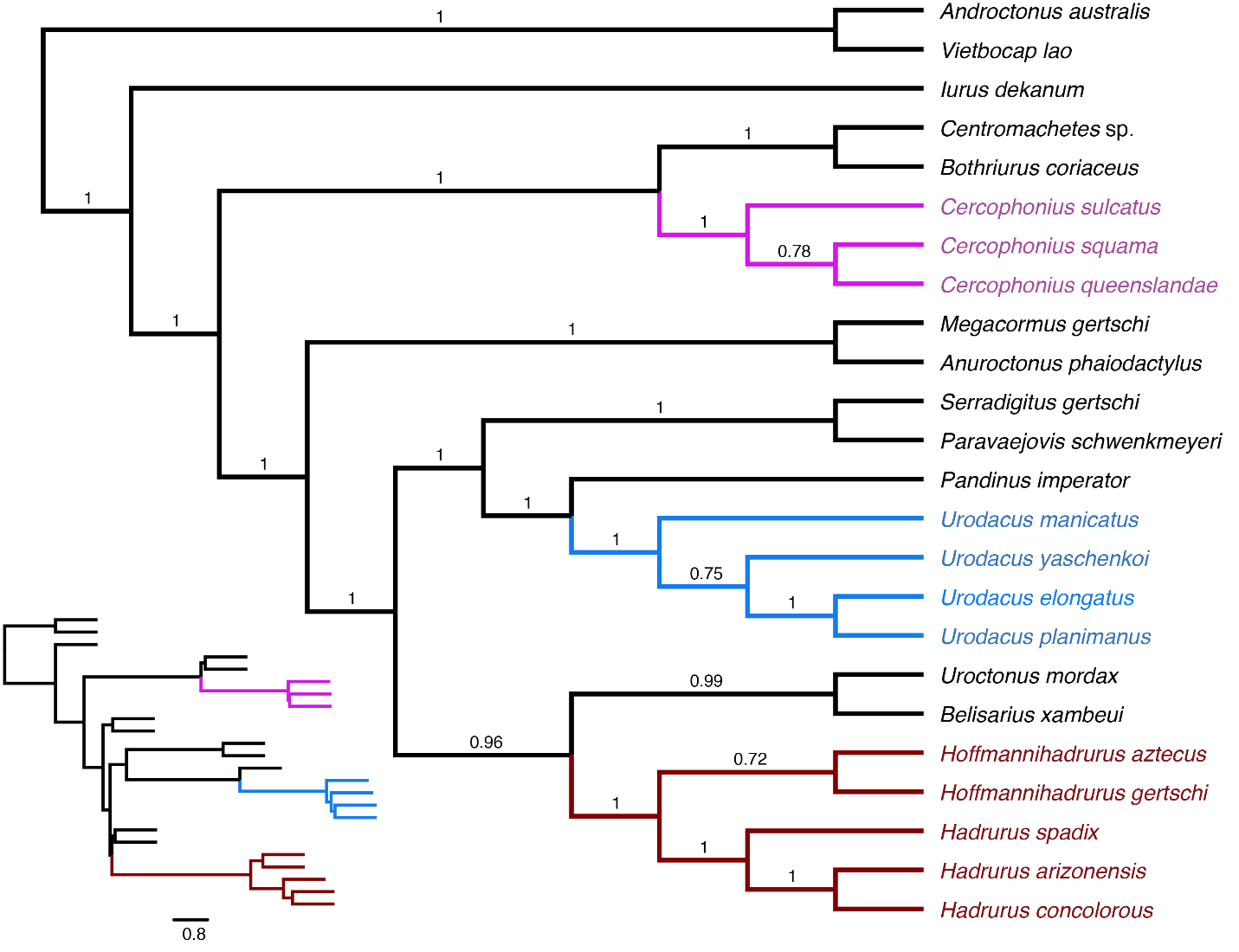


**Figure S1.** ASTRAL-II tree recovered from the 1,982 orthobranches with at least 12 species per orthogroup. Number above branches indicate branch support from local posterior probabilities.


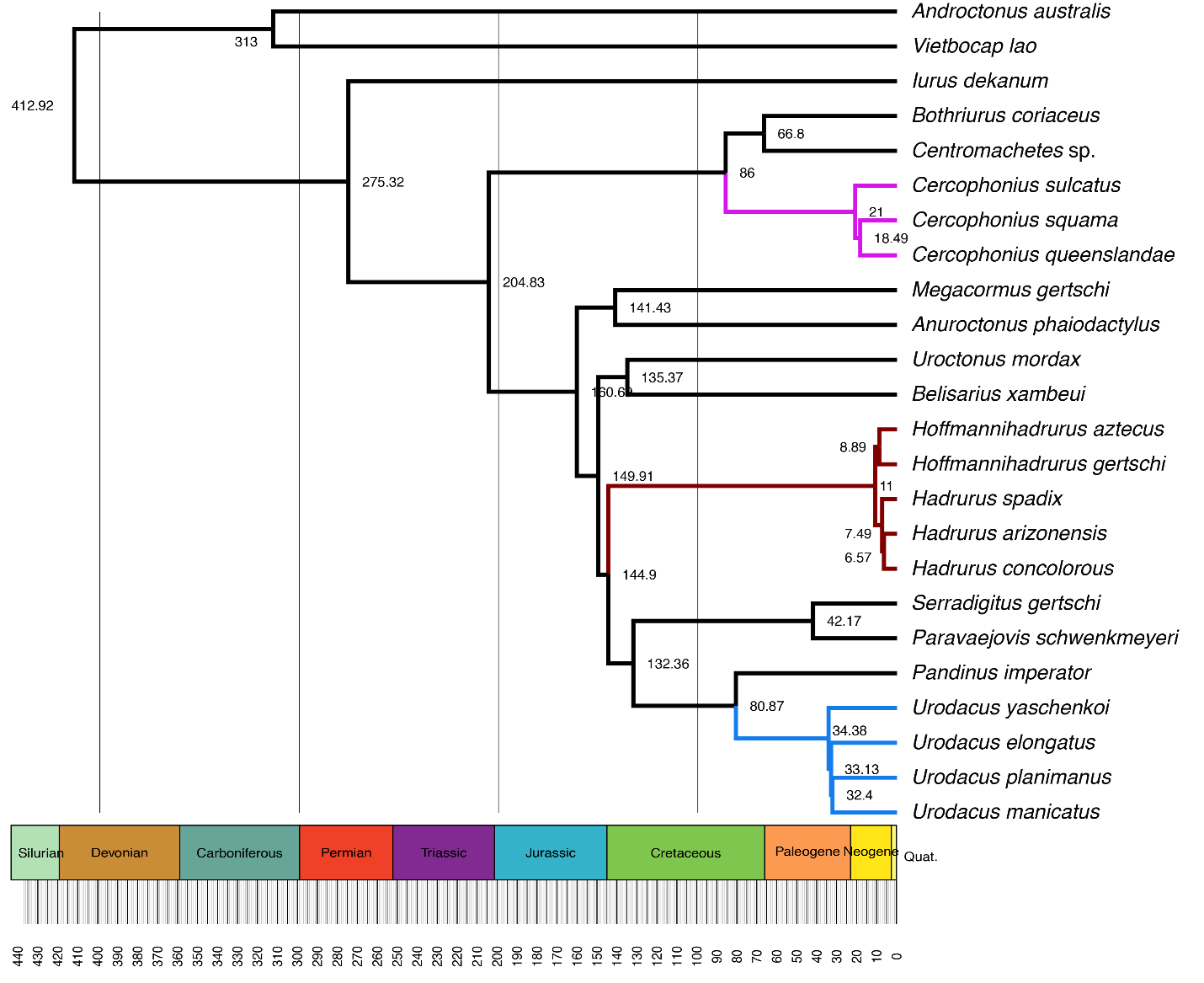


**Figure S2.** Chronogram of the divergence times in our ML tree topology calibrated using the penalized likelihood, as implemented in the *chronos* function under the correlated rates clock model and lambda = 1.


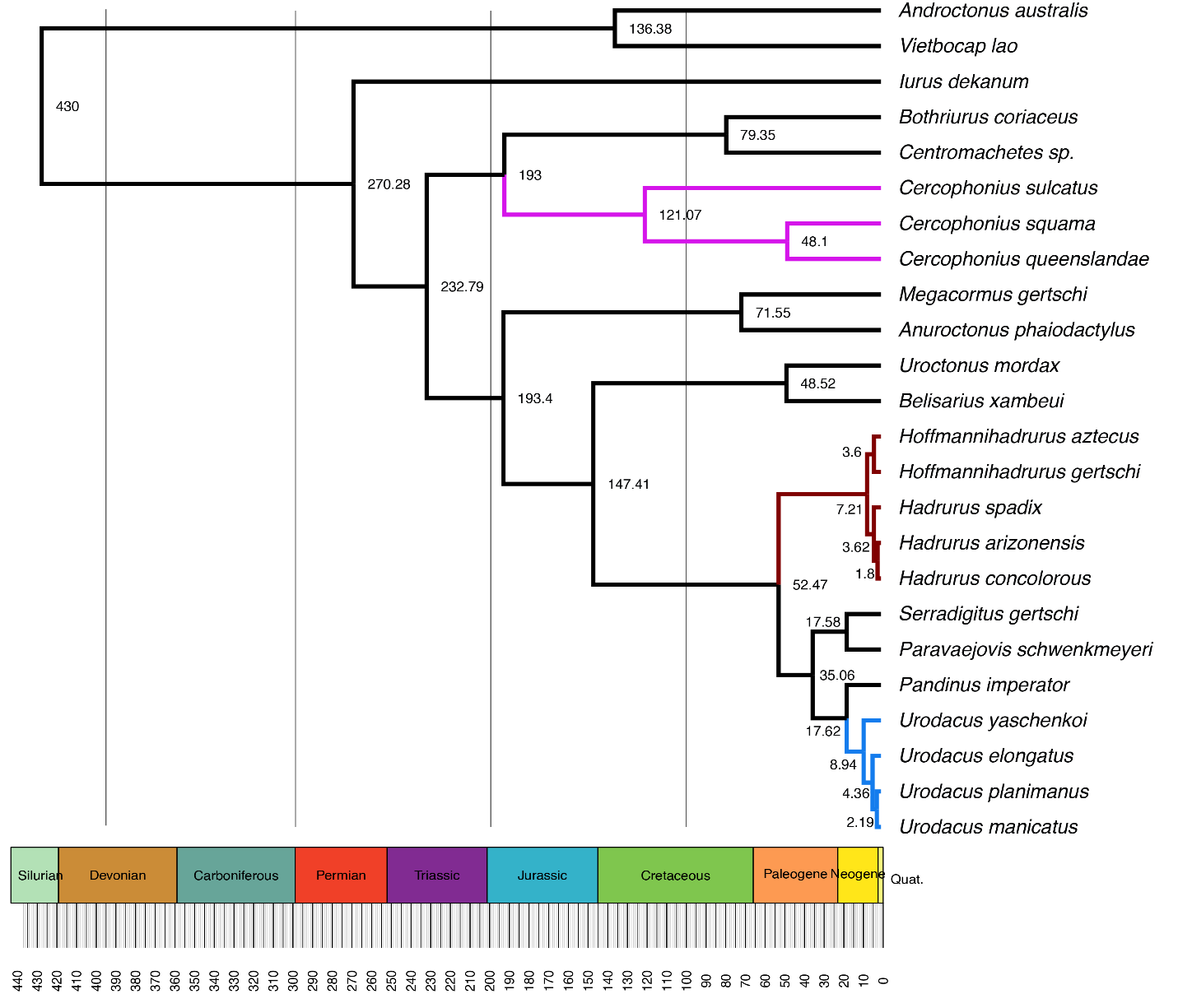


**Figure S3.** Chronogram of the divergence times in the ML tree topology calibrated using the penalized likelihood, as implemented in the *chronos* function under the relaxed clock model and lambda = 1.


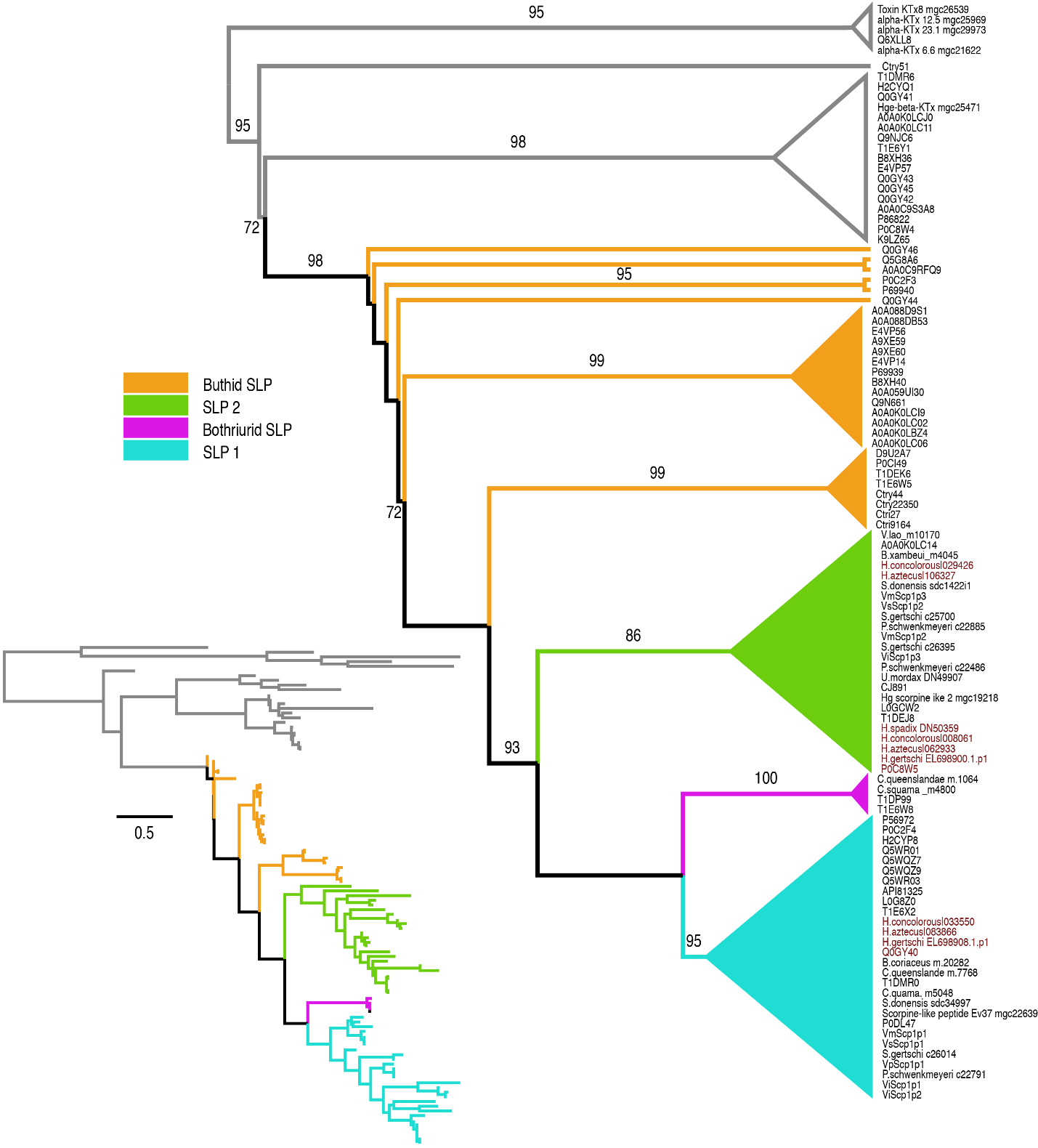


**Figure S4.** Maximum Likelihood gene tree obtained from the analysis of 107 sequences of scorpion toxins (SLP and allies). Number above branches indicate ultrafast bootstrap support values.


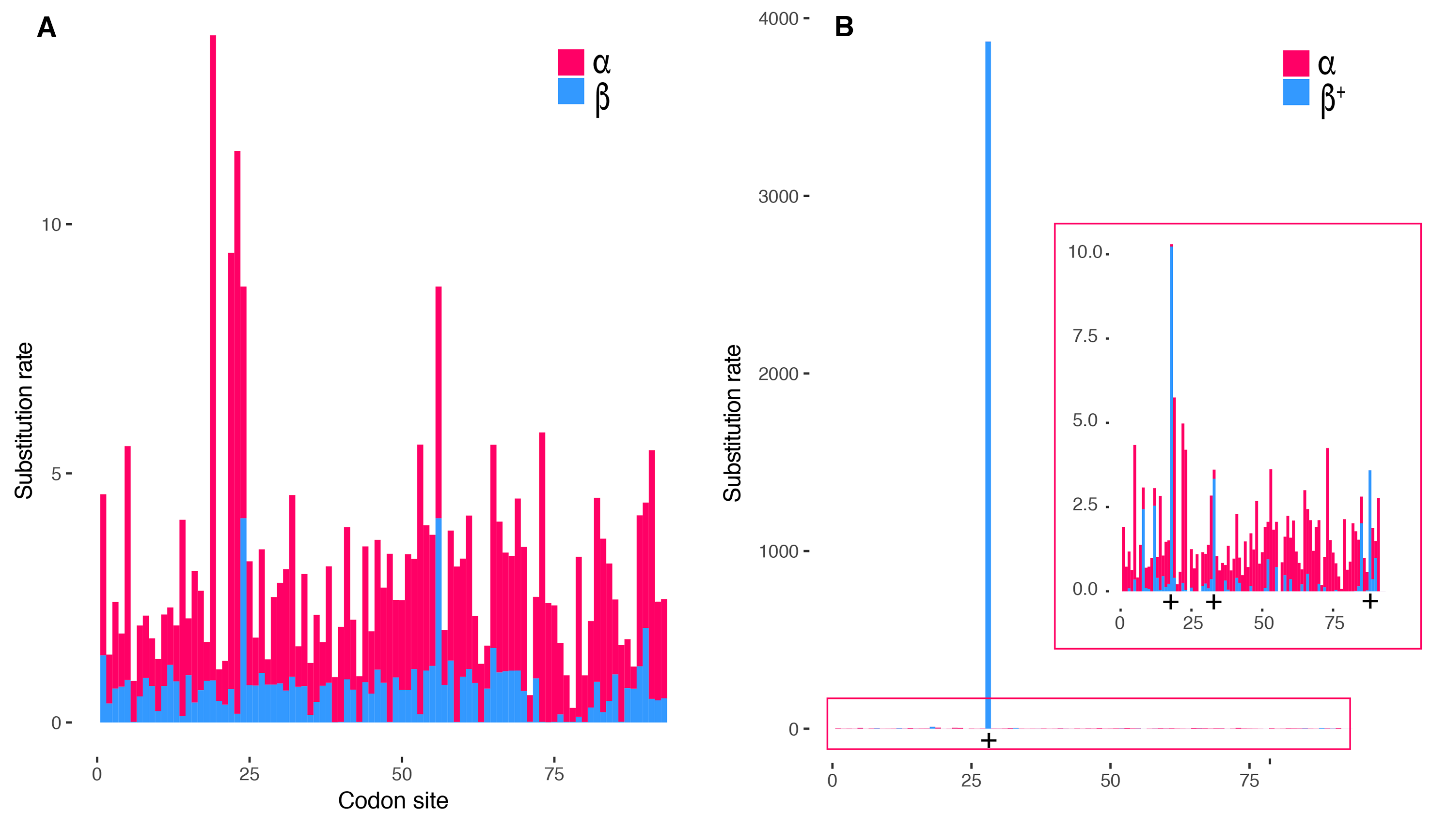


**Figure S5.** Site selection analyses of both SLP1-2 combined with FUBAR (**A**) and MEME (**B**). Visualization of the difference between the values of α in red and β (FUBAR; β^+^ in MEME) in blue. Inset: enlarged area of the visualization of the MEME analysis. + signs indicate β^+^ values were greater than α with a *p* > 0.95.


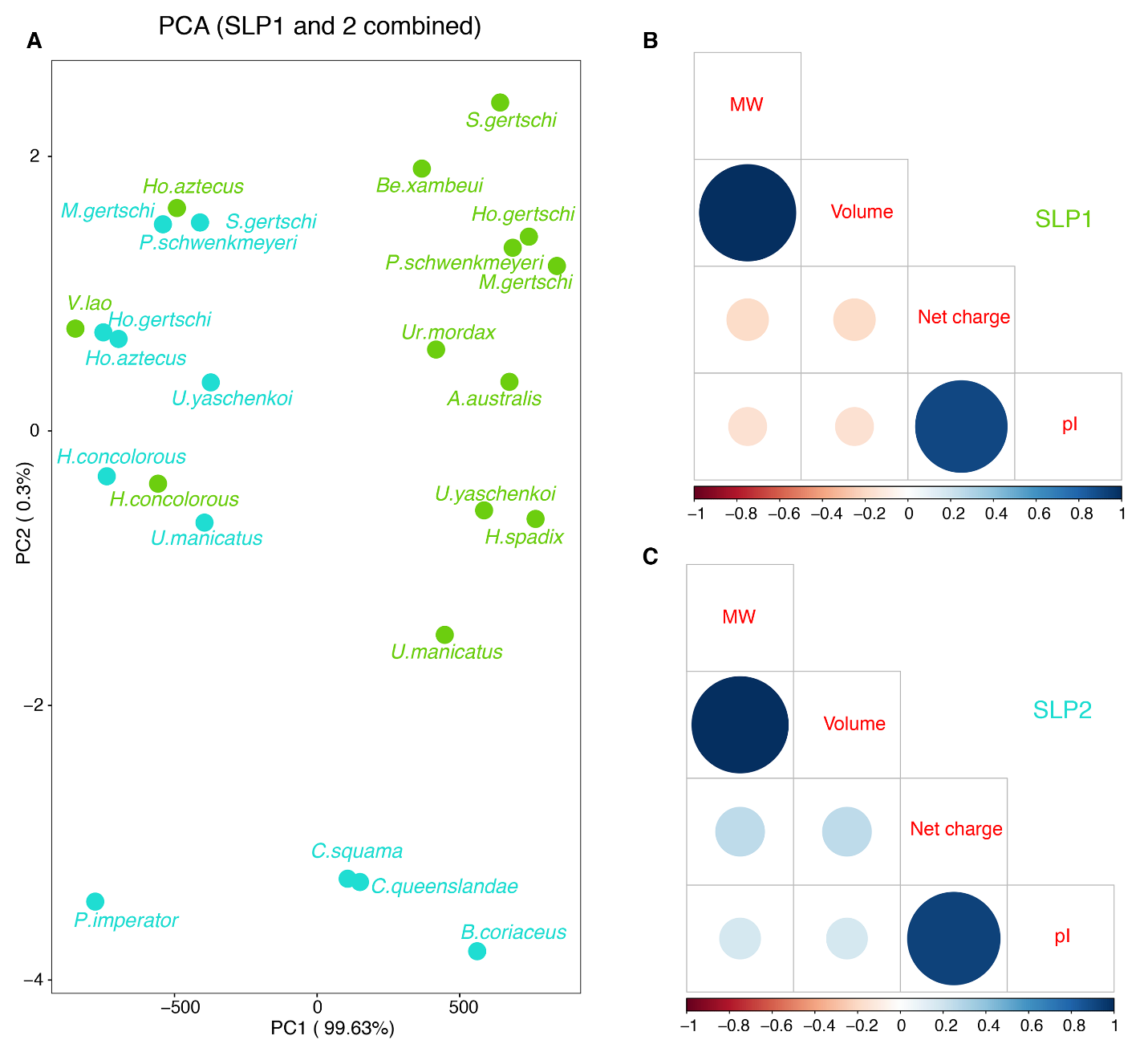


**Figure S6.** (**A**) Visualization of the variation of the four biochemical properties of SLP1-2 in the first two principal components. (**B**-**C**). Kendall rank correlation test between the four biochemical properties from SLP1 (B) and SLP2 (**C**). MW = Molecular weight, Volume = Molecular volume, Net charge and pl = Isoelectric point. Color of the circle indicates positive or negative correlation coefficient, increasing size of the circle indicates smaller *p*-value.


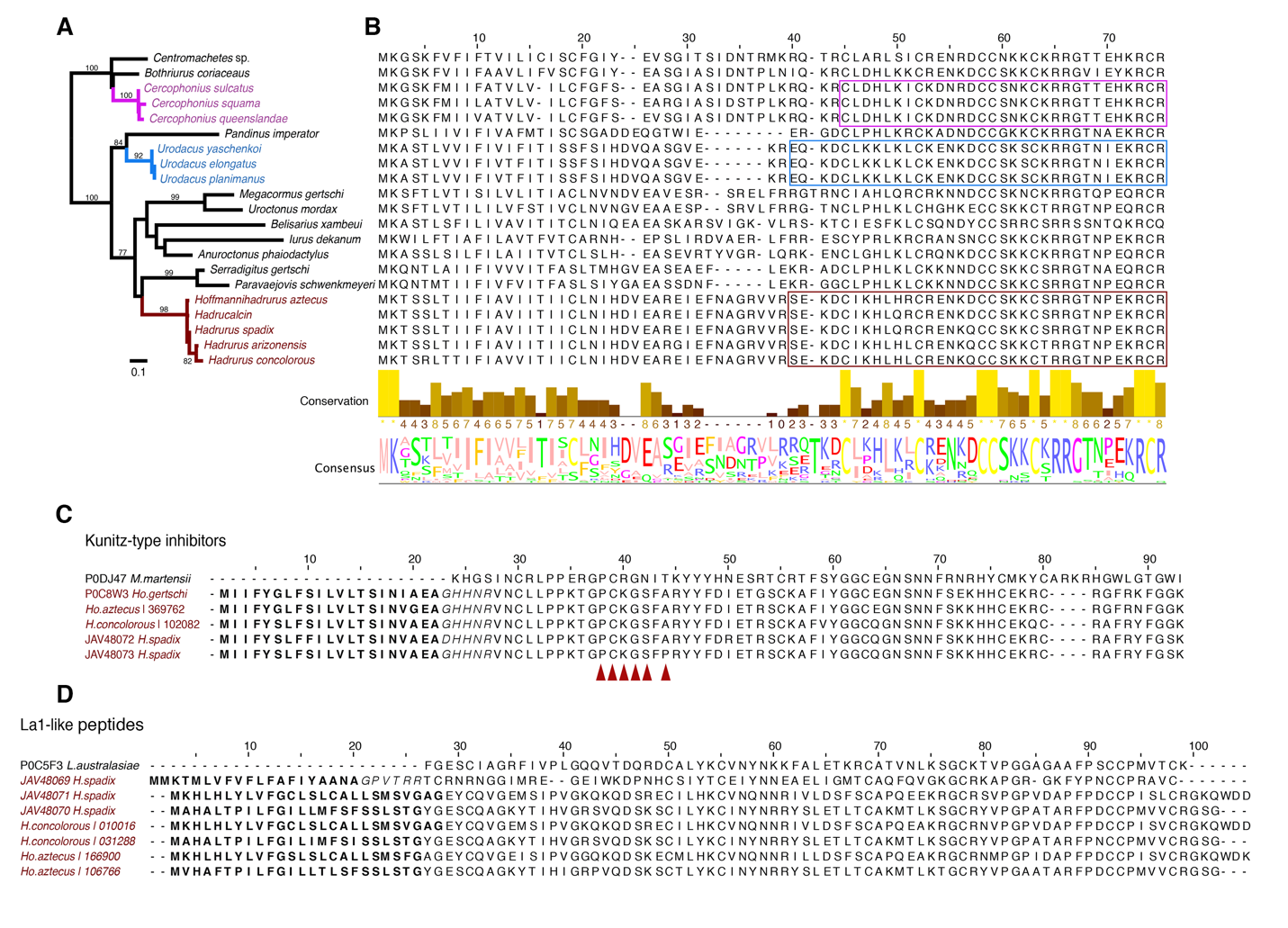


**Figure S7. (A)** Evolutionary tree of calcins (ICK peptides) from ML analysis of 21 sequences reported from iurid scorpions. Ultrafast boostrap values reported above node (>75%). (**B**) Multiple sequence alignment of the calcin full precursor used in the phylogenetic analysis. The mature peptide of hadrurids, urodacids and cercophonids are highlighted in squares colored by genera. (**C**) Multiple sequence alignment of the Kunitz-type inhibitors deduced from the cDNA of hadrurid species and one buthid. Signal peptide is highlighted in bold, propeptide in light italics. Trypsin interaction sites indicate by red triangles. (**D**) Multiple sequence alignment of the La1-like peptides. Signal peptide is highlighted in bold, propeptide in light italics.

References

1. Sharma, P.P.; Baker, C.M.; Cosgrove, J.G.; Johnson, J.E.; Oberski, J.T.; Raven, R.J.; Harvey, M.S.; Boyer, S.L.; Giribet, G. A revised dated phylogeny of scorpions—Phylogenomic support for ancient divergence of the temperate Gondwanan family Bothriuridae. *Mol.* *Phylogen.* *Evol.* **2018**, *122*, 37–45.
2. Sharma, P.P.; Fernández, R.; Esposito, L.A.; González-Santillán, E.; Monod, L. Phylogenomic resolution of scorpions reveals multilevel discordance with morphological phylogenetic signal. *Proc.* *Biol.* *Sci.* **2015**, *282*, 20142953.
3. Santibáñez-López, C.E.; Cid-Uribe, J.I.; Zamudio, F.Z.; Batista, C.V.F.; Ortiz, E.; Possani, L.D. Venom gland transcriptomic and venom proteomic analyses of the scorpion *Megacormus* *gertschi* Díaz-Najera, 1966 (Scorpiones: Euscorpiidae: Megacorminae). *Toxicon* **2017**, *133*, 95–109.
4. Rokyta, D.R.; Ward, M.J. Venom-gland transcriptomics and venom proteomics of the black- back scorpion (*Hadrurus* *spadix*) reveal detectability challenges and an unexplored realm of animal toxin diversity. *Toxicon* **2017**, *128*, 23–37.
5. Schwartz, E.F.; Diego-Garcia, E.; Rodríguez de la Vega, R.C.; Possani, L.D. Transcriptome analysis of the venom gland of the Mexican scorpion *Hadrurus* *gertschi* (Arachnida: Scorpiones). *BMC* *Genom.* **2007**, *8*, 119.
6. Santibáñez-López, C.E.; González-Santillán, E.; Monod, L.; Sharma, P.P. Phylogenomics facilitates stable scorpion systematics_ Reassessing the relationships of Vaejovidae and a new higher-level classification of Scorpiones (Arachnida). *Mol.* *Phylogen.* *Evol.* **2019**, *135*, 22–30.
7. Santibáñez-López, C.E.; Kriebel, R.; Ballesteros, J.A.; Rush, N.; Witter, Z.; Williams, J.; Janies, D.A.; Sharma, P.P. Integration of phylogenomics and molecular modeling reveals lineage-specific diversification of toxins in scorpions. *PeerJ* **2018**, *6*, e5902.
8. Sunagar, K.; Undheim, E.; Chan, A.; Koludarov, I.; Muñoz-Gómez, S.; Antunes, A.; Fry, B. Evolution Stings: The Origin and Diversification of Scorpion Toxin Peptide Scaffolds. *Toxins* **2013**, *5*, 2456–2487.
9. Luna-Ramírez, K.; Quintero-Hernández, V.; Juárez-González, V.R.; Possani, L.D. Whole Transcriptome of the Venom Gland from *Urodacus* *yaschenkoi* Scorpion. *PLoS ONE* **2015**, *10*, e0127883.
10. Cid-Uribe JI, Santibáñez-López CE, Meneses EP, Batista CVF, Jiménez-Vargas JM, Ortiz, E., Possani LD. The diversity of venom components of the scorpion species *Paravaejovis* *schwenkmeyeri* (Scorpiones: Vaejovidae) revealed by transcriptome and proteome analyses. *Toxicon* **2018**, *151*, 47–62.
11. Romero-Gutiérrez, M.; Santibáñez-López, C.; Jiménez-Vargas, J.; Batista, C.; Ortiz, E.; Possani, L. Transcriptomic and Proteomic Analyses Reveal the Diversity of Venom Components from the Vaejovid Scorpion *Serradigitus* *gertschi*. *Toxins* **2018**, *10*, 359.
12. He, Y.; Zhao, R.; Di, Z.; Li, Z.; Xu, X.; Hong, W.; Wu, Y.; Zhao, H.; Li, W.; Cao, Z. Molecular diversity of Chaerilidae venom peptides reveals the dynamic evolution of scorpion venom components from Buthidae to non-Buthidae. *J.* *Proteom*. **2013**, *89*, 1–14.
13. Quintero-Hernández, V.; Ramírez-Carreto, S.; Romero-Gutiérrez, M.T.; Valdez-Velázquez, L.L.; Becerril, B.; Possani, L.D.; Ortiz, E. Transcriptome analysis of scorpion species belonging to the *Vaejovis* genus. *PLoS ONE* **2015**, *10*, e0117188.
