# Supplementary material for: Hadrurid Scorpion Toxins: Evolutionary Conservation and Selective Pressures
Source: Toxins (Basel). 2019 Nov 1;11(11):637. doi: 10.3390/toxins11110637 (PMC6891616; doi:10.3390/toxins11110637)
Supplement: Supplementary file 1 [file toxins-11-00637-s001.zip › toxins-610798-supplementary conversion/toxins-610798-supplementary conversion.pdf]

Supplementary Materials: Evolutionary conservation and selective pressures in hadrurid scorpion toxins

Carlos E. Santibáñez-López, Matthew R. Graham, Prashant P. Sharma, Ernesto Ortiz and Lourival D. Possani

Table S1. Putative venom sequences encoded by 96 transcripts of the *Hoffmannihadrurus aztecus* transcriptome. In bold: Signal peptide. In italics: propeptide cut. Asterisks indicate stop codons.

	**Ascaris-type inhibitors**
Haztecus|153797	**MKFNTVVCTFTILVFCSVLENTLG***QRDRR*
Haztecus|251331	FFFFFSFTVLRMKFNTAFSTFAMFVFCSVLVNSAAQQESRCRRPGEEFTMCGTACPLTCDNYQRPPEACILLCVAGCF<br>CKNGLVRDTQRDGRCVRQSECGK*
Haztecus|375070	**MKWNISLAALVFLLCCLSAKC***MIMRR*
	**Calcins**
Haztecus|000605	**MKTSSLTIIFIAVIITIICLNIHDVEA***REIEFNAGRVVR*SEKDCIKHLHRCRENKDCCSKKCSRRGTNPEKRCR
	**CAP Superfamily**
Haztecus|024941	VGCGVSVYYDNMDNMNKVLYTCNYGPAGNMRGDAVYNVGAPCSQCPKNTQCSNEYK
Haztecus|122761	**MTSMIIPILALWIIITGTSA**YYDCDEKYTNITLDHTMCKIANESCRFLRKGKTFAAQLLRTHNNIRNSIHRFVGKEYPL<br>ATNMELMNWDEELYKIARIHSIQCIEEPDCNLCHQIGDFPVEQNFAVKKFKKSEVENNGPVKRFQTVIKEWAAELRL<br>YDPSVVSNLTITDGLPVNWINILRATTLFVGCASMNFYSDEPGIFKEVYICNYGPAKLTEGEEIYKTGDMSCSNCKDD<br>GMCDTEFKRLCVPNDFEINIRTTFSQNSTRYPWYYRNESYYKETTTNEENSSWEEITETLMETSIVEENATFTVTEIVPS<br>EETSMEEVTEFLSLEETSMEEVTEFLS
Haztecus|157683	*MAIFGTKVLIKR*QLLNSAVVQICLILLVSAYPRLQRPKLYGNAIPLRDLDTRHFKTRRKIVLLHNSYRARVDPPASNML<br>SMAWNDEAAKDAQRWAESCRFLIHDSPTGRWVRNYGSCGQNIFVSNMKVSWSFAIKAWNIERYDFVYGSHKNVP<br>SVVGHYTQMVWYKTHKVGCGFHYCGPNVVKKPFYNYVCNYCPIGNDPVTFNEPYGIGKPCSKCPGKCKYKKLCTN<br>SCKYGDSWSNCAELNATWNNWLCGDPKQQRYQNCLGTCRCSSNYIR*
Haztecus|168586	KYLLRNRFSNNIHVYETALACQLSFNVAFNITFCYSVTMNLFLAILCLQLSSLPYVPCEVCSRSSYGKYDISLSHENTRD<br>EKIFLKELKMSSWDEESSEEQITEMKECPELYERYSTEHTYCKVSTCDIITEGINEDDKELILKIHNELRNKLANGSEERY<br>RPLPSAANMMELEWDDELSRVAQAHASLCVFRHEKNAQRAVENFSVGQNLILSPRAQKDWNVVEKLYKEEVCFFF<br>PEYIKPFHYEGNFGHFSQLTWATTWKIGCGFAAFRENGKTKSLYTCNYGPMGNMLSGTHYIVGEPCSKCPKNTERSE<br>QYPALCKSKTEDGPQMERPSTEDYILYCDFSDEDPQECNDVKITGSRNLTTRHIYSGNYKTVVLERSEFVSVDFGTAQ<br>DEKGICAFLYIRFGPNNANDSIASVLEISYSSDVILPGPPTKIHPLSSSFFTAAVSVNYGGPLKVTAKMRAEENAAPQYF<br>DIKFWGIRKGSCKLFRIIQ*

Haztecus|168601	**MNLFLAILCLQLSSLPYVPC**EVCSRSSYGKYDISLSHENTRDEKIFLKELKMSSWDEESSEEQITEMKECPELYERYSTEHTYCKVSTCDIITEGINEDDKELILKIHNELRNKLANGSEERYRPLPSAANMMELEWDDELSRVAQAHASLCVFRHEKNAQRAVENFSVGQNLILSPRAQKDWNVVEKLYKEEVCFFFPEYIKPFHYEGNFGHFSQLTWATTWKIGCGFAAFRENGKTKSLYTCNYGPMGNMLSGTHYIVGEPCSKCPKNTERSEQYPALCKSKTEDGPQMERPSTEDYILYCDFSDEDPQECNDVKITGSRNLTTRHIYSGNYKTVVLERSEFVSVDFGTAQDEKGICAFLYIRFGPNNANDSIASVLEISYSSDVILPGPPTKIHPLSSSFFTAAVSVNYGGPLKVTAKMRAEENAAPQYFDIKFWGIRKGSCKLFRIIQ*
Haztecus|232457	CLLENSDKKCSMAQKFIIIVLLIVSTGMISNAAMERPMEIMVKETAIKLSNEQKLHIVYLHNVYRRNATPSAANMVYVQWDEALAITAQAWANLCVMKHGIPASPKYDKGGYGQNLYFGPSNSTYRAIWLWYNEFHNFHFRSVNCTGIECGHYIQMMWWETKFVGCALSQCGKSEFFFVCHYYPQFVNNTTPYLVGEPCSLCDLGYGGLCYDNLCVTKPACEKYGLDCKCKLKCHNCGRVNTSTCSCKCVDGWDSLDCSSQCRDQDEKCGKKDGFSSILSCSMHKGKVKKKLCRKMCQACGSVNTTNLSRTCCDGIICSPGYVIDLDNRPCSCRPLCPGPECYSSVGKIWKDESFSLAFGILALLWKTWKV*
Haztecus|237566	RGVTEEDKKIILDIHNKFRNKIALGQETSPRQQPPAANMIQMEWDNELAKIAQAHSDQCVFEHDNAPQRQVENFSV
Haztecus|293319	**MIVQLLLSFALFSWDVASS**QTCPEIYLRFSKDHTYCLLSNCHVTKRGVTEEDKKIILDIHNKFRNKIALGQETSPRQQPPAANMIQMEWDNELAKIAQAHSDQCVFEHDNAPQRQVENFSVGQNLLITMLSKTINWRKISMWYTSEINYFYPQYRQPFTFATAYGHFSQMVWAETWKVGCGVSVYYDNMDNMNKVLYTCNYGPAGNMRGDAVYNVGAPCSQCPKNTQCSNEYKGLCKSLTPDGPQKEISISSRDFLLYCNFSVNDSPGCRNVQISGSKPFQTKKLYSGEYKTAILNGGESITIKLGKAQDNRGICPFVYGSFGPNRDGDAKRSAVSIGFSAPRIMFGDPVKIEYGSSEFWTIGMLMRFSGEMESTIKLQAYPGASPQYFNVKSFGIGRGKCPKF*
Haztecus|304512	GQNVAHHWSYNYKDPVQKDKADWVFSIGHWSSEYEKFRFNPAHISPFKFNNKVGHFTQMI
Haztecus|375728	*SVNRMESYLRSFVTLVVLSTQSKFHFATEVVSFRENSTYSWIHQATDRHSLKPSRWARQAITLSESERKKAVHLHNLYRSMVIPSAANMEYMEWDHR*LAALAQRWAENCQWKHGNPPHKFPNGVGQNLYKGPSAYISTAMGLWFDEYKDYNYFNIKCKPYKKCGHYTQIVWATTRYVGCAVVSNCWPDYKYYTVCNYWPPGNYQNTKPYIEGKPCTRCNFTGNGLCSKNSCVSRDQCKRH
Haztecus|387397	*FTQMIWGETR*MIGCGYVFYVHPKQGYTKSYICNYAPGGNVIRGIMYKISPEGAKCSFPVLHPSKEYQGLCENGR*
**KTx**	
Haztecus|368213	**MAKSFFAAFLIIMLISSLVDG**KSTVGQKLKKKLNQAVDKVKEVINKSEYMCPVVSSFCKQHCARLGKSGQCDLLECICS*
Haztecus|092578	**MRLAIILLLMTILVLTVG**APPSGTKCSSSNQCTRPCRYGGGTHGKCMNGRCRCYG*
Haztecus|315193	**MKFILILFLLSIYMSTCFPSIEG**IKCYDSGWCREPCRHKGATYGKCINNSCMCYR*
Haztecus|139672	**MNTKLVLMMLMITAVILVFEAETVTG**TGTSCINPKQCTEPCRAKGCKHGKCMNRKCHCMLCNR*
Haztecus|224197	**MNTKSICFVFLVTLMLAFDRIEVEG**VKKCSVASTCVKYCWKYEKCSRGKCINKECKCYNCRG*
Haztecus|364908	VKKCSVASTCVKYCWKYEKCSRGKCINKECKCYNCR
**Scorpines**	

Haztecus|062933	**MEIKLTILILLVITSFCSC**GILREKYAHKAIDVLTPMIGVPVVSKIVNNAAKQLVHKIAKNQQLCMFNKDVAGWCEK SCQQSAHQKGYCHGTKCKC
Haztecus|083866	**MNTKLTVLCFLGIVTIVSC**GWMSEKKVQGILDKKFPEGIIRNAAKAIVHKMAKNQFGCFANVDVKGDCKRHCKA EDKEGICHGTKCKCGVPISYL*
Haztecus|106327	**MQLKFLVLLLFGMTTFCTC**GILKEKHFHKAVDYLVPAVVPDVVKNLAKQAIHKIAKSESFCMFGQDVRKLCNKSC METLNMKGNCHGTKCKCEP*
	**Kunitz-type inhibitors**
Haztecus|369762	**MIIFYGLFSILVLTSINVGEA***GHHNR*VNCLLPPKTGPCKGSFARYYFDIETRSCKAFIYGGCEGNSNNFSKKHHCEKR CRGFRNFGGK
Haztecus|016332|	MSNIKPWMPNMAVINVIYWPLFSDACNQPPNPGLCFARFERYYYDPSTNTCKLFVYGGCKG
	**La1-like peptides**
Haztecus|090690	VQRNIFPIIPETCMTDGITIPLNEEKQDQTKCVLFRCEKNAGRIVLNTITCAPQEPKRGCQNIISPVNLPFPDCCPLVVC KHPIFYNGYK*
Haztecus|090697	NDFRNMTKSTAATFVILFASTYFVSCLAYGETCMTDGITIPLNEEKQDQTKCVLFRCEKNAGRIVLNTITCAPQEPKR GCQNIISPVNLPFPDCCPLVVCKHPIFYNGYK*
Haztecus|106766	**MVHAFTPILFGILLTLSFSSLSTG**YGESCQAGKYTIHIGRPVQDSKSCTLYKCINYNRRYSLETLTCAKMTLKTGCRYV PGAATARFPDCCPMVVCRGSG*
Haztecus|166900	**MKHLHLYLVFGSLSLCALLSMSFG**AGEYCQVGEISIPVGGQKQDSKECMLHKCVNQNNRILLDSFSCAPQEAKRG CRNMPGPIDAPFPDCCPISVCRGKQWDK*
Haztecus|294468	YTTLFRSGEYCQVGEISIPVGGQKQDSKECMLHKCVNQNNRILLDSFSCAPQEAKRGCRNMPSPNGA
Haztecus|132887	**MMKTTLVLVFLFAFTYTANA***GLVTRR*
Haztecus|118284	*FLFAFTYTANAGLVTRR*TCRNRNGGIMQEGEIWKDPNHCSVYRCVIYDNEAELIGMTCAEFQVQNDCKKVPGR
	**Phospholipases**
Haztecus|009989	*SRIRR*SIMQLAGMLRCTTGCDPLAYKSYGCYCGYLGAGRPVDDIDSCCREHDRCYSNTICPQLFLYFFRYPWRCTSLGI PQCGRMPGVTIAQQCARQLCLCDREFVRCIGRFPCPRQSAICRTNPLGVLQSLIVGKR*
Haztecus|027670	MLKKTELTKRKWNGWIKPSCIPITIIFILIVLVVMLPLLDQKHEDKVHLLTNPSNCTEPCWATLVESIPENMTYNISLN HPSTFEGWLNLIQLAEHTIDIAAFYWTLRGRDVISDPSDWQGEKIFDDLLAAGTERKIKISIVQNLPSHIFPDNDTKELE EKGAAAVRNMNFEKLLGKGILHTKMLIVDDKHFYVGSANMDWRALTQIKELGIIVYNCSCLAKDVKKIFDAYWLLS ETEHIPLPWPSYFDTTFNKTHPAVLTINNTSTYAYSSSSPPQFCAKGRTSDINSILSVIHKAKKFIHVAVMDYFPALIYTK HLKYWPVIDNALREAAITRRIKVKVLASYWKHTRPVMQAFLRSLQSLNSTDISIETKLFEVPVYSPSQEKIPYARVNHN KYMVTDNSAYVGTSNWSGDYFISTGGVGFILENTDNSTNSSIQIQLQDIFERDWSSEYAHPITEIDIR*
Haztecus|077087	**MRNFEERKYGRCIMLLLKLTVFILLFVQPAPS***TDLPLPLPHEKKLTAFYQEKQNPYMLIIGR*

Haztecus|077100	*MRNFEERKYGRKEADRVLAALHLDDVEKIEHRR*MESLTKFCAKEKHKFIPKTYTKKVFIYPGTKWCGMGDNAANEDELGKEKEADSCCRDHDHCKDSIPAFSIKHNLTNYSPFTKSHCDCDKQFHTCLAMARTKTATIISNLYFNLLNMECFEHKTCSSNETCITWQWKLAKSDGSPIQ*
Haztecus|134320	*MTASPQPQIKTVHQLQNMLALFWKSVNSNYNKMPSSYVTGLFKTKQWKNAVKNWKISEFAKIVGIMQFHWKFPILLQSKINKNTENLPILKSDTQRAPVTKKTEPTQILELDKALTDWINDDKKPDNNLSFQDNRNSLDIIKSKPTSSVLGNLEKVLSSWFQGDALTFGFQTSKNWMMAQKDESSLTTTYKVTKINVESR*SRFLVKSLSSASSETSILYRLQEMGKHLLRYPDQKDIMVKENAIPVTLRIRESFSNTIQAKAQEILSLLGYSDPPGSAGVRILSIDGGGIRGMIAIEILRYLENVSGKPVYQLFDYICGVSTGAILSMLVGGLHLPLDECERLYVKMSSELFKRSAFLGTSGLIWSQSYYDTSMWVNMLRKTYGEIMLSETCNKKGNPKISCVSAVMNQPILEAYAFRNYSLPYRKQSLYPGSSNHKLWQAIRASAAAPGYFEQYTLAGIVHQDGGLIMN
Haztecus|139911	**MAFLCLTTLLILISLRNA***RIIQRELYLNLEPVSNQKDLLPVARAAVVNFDEGFETGR*
Haztecus|162449	**MNILFIAVFLNIITNFDVKS**QSILRGVQKALDSVSNFIDDISFGIKQVRDGLSTVDDIVNYAQGKPCQYICPPGLMLKKNYYYKPIPQGCGAYGIQVVLSLPLLKDTEKCCDKHDICYSTCMTNKTDCDAEFGTCLYKKCEKQAKKLGEDVRKCTGVSKLFHLGVQSLGCNAFKAAQAEACRCASKDEL*
Haztecus|183750	**MGLIMVLVIGVLSADAVLS***MDNELYLNLEPSQRSSWPVARAVRMQFSKRSEGGRESRR*
Haztecus|305099	**MTFWCTLILLTLLTVCCRINECRS***LHRKPRSLPQLARMVEATTGR*
Haztecus|311460	DDYDDLGVYEETDKCCRTHDHCNDSIVGFETKYDLKNKDFYTKSSCNCDLRFHSCLYKKEAIHSDAVGHLFFNILQTQCFKDEYPIVKCLKKWGIPLIRETCQKYKLDCNGTKKYQFFDAKMYKGKNESPFLKKLLSH*
Haztecus|330410	**DTPCSNEEAIMKTSLVFVLATLSLG**ECVIFDSVSDVLPLTTTFYREKDGHRMVETIEVNPYLSEKKTIDCYMYGNNYIIDRMLELVPESVTKESDKTDISKLVNQCSDLLLNELNNGIFHSVKSPFDSVRNAFKSLLIFPGTKWCGAGNVADNYEDLGRAEQTDKCCRTHDHCNDTIPGYETKYGLKNENFYTKSTCPCDLTFHSCLYEGNNLPSDLVGKTFFNILLMQCFKKEYPQIGCLEKTGIILRSCQEYKFDYNGTKKYQFFDAKTYEPKENASFLSRLLSG*
Haztecus|355671	VNYTRYNLLEEKCRDFHKLIQASLGRRRIRRELFLYPGTNWCGFGSSVKKFGELGYNTHADRCCRDHDLCPFTISAFSKRYDLFNYRFHTISHCECDERFRACLKLANSPTANIVGRLFFNIVQIKCFSFRVENVCSKWSWWGRCLLNENKTTASLKG
Haztecus|384314	EDKCCREHDLCDDIIQAGGRKDDLVNDSKYPTLLCSCDDRFWHCLRDINSLTSNAIG
Haztecus|402536	IEDPVSYISFYGLRNYSMLNDKLVTELVYVHSKLMIVDDRIVIIGSANINDRSMIGK
	**Hyaluronidases**
Haztecus|032949	*WMLKTLELTKKMRPKGR*WCFYHLPDCYNYAGKDKPEQFLCSSLVRKHNDRLIWLWNATTALCPSIYFDERQTKY
Haztecus|032956	*KYNESQQIWFIYGR*LSEVLRVSQPRTPIYPYINYRIHSSLEEVPKERFWLMLGHLASLGLDGAVIWGSSNYVKTEEECRNLELYVKEVIGPASSTISSNVNRCSEVICNGEGLCTWPHQSYTSWKYLTNQNASAFKREDITCR
Haztecus|047421	LGEKLVIFYENKLGKYPYIDPKYGDVNGGLPQLGNLEKHLQAAEKDIQTIISNPNFDGLGIIDWEKWRPIWDFNWGKMRIYKTRTMELMQKKHPSWPWKLIENASRNQWEETAKQW
Haztecus|047425	*ILFDVPELANLSEHLEIARR*DIEKTIPDPNFDGLGIIDWEKWRPIWDFNWGKMRIYKTRTMELMQKKHPSWPWKLIENASRNQWEETAKQW

Haztecus|085528	LFPDCYNYEGKDQPSQFFCSKNVQTYNDRLSWLWEASTSLCPSIYFYNSQMKYSDHQRLWFLYGRLAEAVRVSSPST<br>LIYPYVNYKSPDILSDVPEEHFWRMLSLSASMGLDGVVIWGSSNYVQKKEDCKALASYVKKVIGLSSLTVSKNFNFCS<br>KTTCRGQGRCVWPEEPYTSWRYMCNRDLSDFQPGEIICRCQTKKGRYCNLSNVKLN*
Haztecus|136074	MLWLVRTIAVFSIFIFGGKANFNVYWNVPSAPCSKKYGINVTDDLMKHRILVNNGEQFIGNKIVIFYENKFGKYPYID<br>TEKGIDVNGGMPQLANLTEHLKTAEKDIDNMIPDPDFDGIGIIDWESWLPIYDYNWGKRSIYRTRSIKLIKKMAPPLH<br>AEIIKRIAGKQWERIAKQWMLETLELAKKKRPKARWCYYLFPDCYNYKDKQKQSEFCCRESVRANNDRLSWLWEA<br>STALCPSIYFYNKHMNYTYHQRLWYVHGRLSEAVRVSSGKALIYPFVNYLCRDKDNNILFDVPEKHFWQMVSFTAS<br>MGLDGVVIWGSSNYVKRKKDCEALASSVKKVIGPSSLTVSRNFKRCSVIICSGVGRCFWPEKQFVSLTYLSNKDLPQT<br>FFKREETACRCKKNLGRYCNVSNLDPHHLNEDCPEVH*
Haztecus|136097	MLWLVRTIAVFSIFIFGGKANFNVYWNVPSAPCSKKYGINVTDDLMKHRILVNNGEQFIGNKIVIFYENKFGKYPYID<br>TEKGIDVNGGMPQLANLTEHLKTAEKDIDNMIPDPDFDGIGIIDWESWLPIYDYNWGKRSIYRTRSIKLIKKMAPPLH<br>AEIIKRIAGKQWERIAKQWMLETLELAKKKRPKARWCYYLFPDCYNYKDKQKQSEFCCRESVRANNDRNISGKWFL<br>SRHPWV*
Haztecus|136098	*MVSFTASMGLDGVVIWGSSNYVKR*KKDCEALASSVKKVIGPSSLTVSRNFKRCSVIICSGVGRCFWPEKQFVSLTYLSN<br>KDLPQTFFKREETACRCKKNLGRYCNVSNLDPHHLNEDCPEVH*
Haztecus|178545	**MLFFVCIVSIFNNIEA**SFDVYWNVPSHLCSIKYDVNMTETLSKYNILVNDGESFTGDKITLIYENGIGKYPHIDPNKGD<br>VNGGLPRLDKLKEHLNLAEKDIEKIIPNPDFSGLGVIDWEAWRPIWEYHWGGLSIYKSRTIDLVKKDHPTESDQFIETT<br>AKNLWENTAKQWMLKTLELAKKLRPKGQWCYYLFPDCYNYFGKDHPSEYFCSTMIQNNNDRLSWLWDASTAFCP<br>SIYFIENQMKYNESQRTWFLYGKLAETARVARPSTKIYPYINYMVHVSQIPVPRDHFWKMLALIASMGFDGAVIWGS<br>SSYLGSEKSCNDLESYIENVIGPAVTTISSNVDRCAQMICNGRGRCTWPNEPFISWQYLTDTNGPNFDSQKITCRCQS<br>HSGRYCS*
Haztecus|317745	FDGLGIIDWESWRPIYNYNWGTMTIYKTRTVELVRKENPSLKVDLIKSIAEKQWEEIAKQWMLQTL
	**Metalloproteases**
Haztecus|025479	LLHGVHKPVKLCASANGKFDVGRAKSMARMFGQQRALILVFFATIGLSGQQDDNDAHDGRQCDCMEYWQCIGA<br>GGKPYSYCVYTNKVCCFVDRNAKSVGILPRRSKTASCGQKGIDNGREGFSEPGEWAWHAAILESPRDLYVCGATLV<br>DEYWVMTAAHCVDDFSNVLKLKIRLGEYDVTRSSEPLRHEEFEVSRVVLHPGFDNSTLLHDIALLRLSTPAKRRRHIN<br>TVCMPETGMTDNQLFGSKCFVTGWGKRNEKSNHSVILKEVNVPLWKNSECEIALRRQFGPHYELPSSVICAGATGR<br>DACDGDGGGPLVCEKNKNWYQIGIVSFGIGCGRPKTPGVYTRVHSYRQWIHDVILHS*
Haztecus|027032	**TGFQVHKMAVRSMCLFTTWSLLLLFFGSKQS***VSQDRR*
Haztecus|030723	QFQCGAVIITNRWLLSAGHCFVHTRDSYWVARLGLLRRGSDLPTPYEEVRRITHIEVNPQYINKNFINDIAVLKLETA<br>VPFSNYIRPICLPEPEDDVTKWNGKKCSVVGWGKLGEQGDRFPDTLQEVQLPVISTEECRKRTLFLPIYQITDNMFCA<br>GYERGGRDACLGDSGGPMMCQKENGRWVLIGIISNGDGCARAARP
Haztecus|103421	**MRWYQVIVVISTLFVQHVVWT***ESRAVSGEDPNEMEVEIVYPRVYHMSRKKRDVGSSDETR*
Haztecus|103449	**MRWYQVIVVISTLFVQHVVWT***ESRAVSGEDPNEMEVEIVYPRVYHMSRKKRDVGSSDETR*

	**HDPs**
Haztecus|049505	**MNAKIMLVVFMITMFVTDQVEG**GFGSWVKKLWKSKLGKAARKAAGNFIAKKLENAAAPAEGGSKRFDEFMNSL YY*
Haztecus|049972	**MNAKAFLAIFMIALLVTDQAEA**GWWNALKSIGKKLLKSKLAKDITKMAKQRAKEYVVKKLNSPPEEEVAAIDAL MNSLDY*
Haztecus|270640	IKGPVKGNGHCLSATFVDKKMNAKVFLVVFMIALFVTEKAEAGILDTIKSIASKVWNSKTVQDLKRKGINWVANKL GVSPQAAASMTLDEIMDALEDY*
Haztecus|314185	**NPQPSVHKKMNVKVFLLVFMIALFVTEQAEA***GIWESAKSIGMQVWKSKPVEELKR*
	**Other inhibitors**
Haztecus|048272	VIEVNEEGSEAAGASAILIVPYSSAFSSIKSFYANHPFIFFIRDDRTGIVLFYGR
Haztecus|133931	**MLSQINSKMKLLIISCIIVVVYA**REMSECELHREKELEKKPLARLIPECEENGDYKGLQCESGTRYCQCWDKNGTSIT ALSMKLKACECQREKKTAEDKNLIGKFIPSCEVDGTYSKKQCVASTGMCWCVDETGNKISEPTRDEIHC*
Haztecus|133935	**MKLLIISCIIVVVYA**REMSECELHREKELEKKPLARLIPECEENGDYKGLQCESGTRYCQCWDKNGTSITALSMKLKA CECQREKKTAEDKNLIGKFIPSCEVDGTYSKKQCVASTGMCWCVDETGNKISEPTRDEIHC*
Haztecus|176774	**MKAALIYVVIFTFVVATFA**KKTTECQESREKALKSKACITAIVPVCDENGDYAALQCQEGSKFCSCWRKDGTPIIQPS EKIKACECHRQKDEKSSKGLLGTFIPQCAEDGKFQKIQCWGSTGYCWCADPKTGRNTSVSIRGRPNC*
Haztecus|176784	MKLTKMIREKTMKAALIYVVIFTFVVATFAKKTTECQESREKALKSKACITAIVPVCDENGDYAALQCQEGSKFCSC WRKDGTPIIQPSEKIKACECHRQKDEKSSKGLLGTFIPQCAEDGMFQKIQCWSSTGYCWCADPKTGRNTTDSVRGRP NC*
Haztecus|176796	MKLTKMIREKTMKAALIYVVIFTFVVATFAKKTTECQESREKALKSKACITAIVPVCDENGDYAALQCQEGSKFCSC WRKDGTPIIQPSEKIKACECHRQKDEKSSKGLLGTFIPQCAEDGKFQKIQCWGSTGYCWCADPKTGRNTSVSIRGRP NC*
Haztecus|190709	LHSIISQTSGQVIYKYDTKTGYPLSNFCINFAVSSQTITVLLSRSFFRLTMKILLALAVLTVTVSCQPQFGGTGCKPYEEY REASCEVTCDKVLSVTCLTAEKRPGCYCKIGTIRDEDGQCISTEVCSKRVCTKKNQRLDMSGCFTVCTGLGTSYFGCP FVQQPGCNCERGFAVAGGGIRGDCIPVSSCRDPNLDN*
Haztecus|349912	MKSAFSDEADFSGISEKYELQISAVIHKAVVEVNEKGSEVAAGTGIVIKPCSARLPSRHYFRDGNRAGMILFLGRVND L*
Haztecus|365265	*WEETVFFSPASLFIGLAMLYRGAR*SKTSAEMSHALSYDVAGLEATNLHRRIR
Haztecus|377457	MKPNLETLGMRSTFRNDANFKGISTKANLKVSKVIHKVIVEVNEAGTEAAGATALTVVPYMLQETWYFNVDRPFLF YIRDDVNGVILFAGRINQL*
Haztecus|382099	**YSCLSSRSDKIKMKLFILVCFMLVTLSLA**EQTPCQAKREKILNQNLDVEVIPECDENGNYKAKQCKKNGVDCQCW RTDGTPINDFSPNLKACSCVRSRDDANRPHLIGNYKPQCEADGTYSLTQCWGSVGGCWCVDAEGRKLTDKHFP
	**Other venom components**

Haztecus|018156	**MRLYLFVLAVLIASCHC**APRNRCTQECGPVPSNCRAGVTKDYDGCCAICAKAEGEECGGMWNAYGVCGIDLICDTNGNLISNYDLPIGICTSARRIASRNILKRTLRGLH*
Haztecus|031281	**MGFKFCFTAFILTAIFVNVMT**LRCRVCGTYECLPPPTNCPVGTVTDVCNCCLVCGKAENEICGGDWDLRGKCGNGLRCVKTGKTGVCKKE*
Haztecus|068800	VSDSIRVFSVFSGVITDEGLICSCNDVLCQETGKCALGEVKGVCECCNECARVRDEPCGGIYNYAGICGTGLKCEPNDFKQLPGICIPEK*
Haztecus|068805	**ELLTILAYKLSLFFVDLTSQIKDLKMLYFLTFLTISIA**LCRSGVITDEGLICSCNDVLCQETGKCALGEVKGVCECCNECARVRDEPCGGIYNYAGICGTGLKCEPNDFKQLPGICIPEK*
Haztecus|075169	**MFRTTLLSLLVASAYS**LICEPCIFEDCNDVPTCPLGVTKDVCKCCDECFKMEGEICGGPYNVSGICGEGLRCNKGVELYPFHFRNAFGICTLKT*
Haztecus|076767	**MLRLILLCILVASVYS**LSCPCWYDEDKTKYCPPPPTNCPIGLTLGPCGCCLECYKDKGEVCGGSWQILGKCGGGLKCEKGFNDLGSDYYYANHKEGVCQPIEPIDPLIE*
Haztecus|078916	**SCKQDRSKMWFRFIVLFLFVAGVYS**LSCPCHTNRLLCKPAPTNCKLGLTKDACGCCDVCYKIEGEECGGPWKTSGNCGKGLKCVVPENLPEHVKWQAIGICKVE*
Haztecus|110763	**MFRLILLCMLVASVYTL**SCPCLHELDRTKNCPPPPANCPLGLTTDACGCCPVCYKDKGEACGGPWKIIGKCGKGLTCVKETSVSKPKGYYIDQIAGVCKPIAIN*
Haztecus|110769	**MFRLILLFISVASVYSL**SCPCWREPDKTKYCPPPPTNCPLGLTTGPCGCCLQCYKDKGEACGGPWKIIGKCGKGLRCVKETNVGKPKRYYIDQSEGICQPIDTY*
Haztecus|133931	**MLSQINSKMKLLIISCIIVVVYA**REMSECELHREKELEKKPLARLIPECEENGDYKGLQCESGTRYCQCWDKNGTSITALSMKLKACECQREKKTAEDKNLIGKFIPSCEVDGTYSKKQCVASTGMCWCVDETGNKISEPTRDEIHC*
Haztecus|133935	**MKLLIISCIIVVVYA**REMSECELHREKELEKKPLARLIPECEENGDYKGLQCESGTRYCQCWDKNGTSITALSMKLKACECQREKKTAEDKNLIGKFIPSCEVDGTYSKKQCVASTGMCWCVDETGNKISEPTRDEIHC*
Haztecus|159042	**MRIKFCLITFVFLGIFSSATS**LTCLICGTFECSPPPNNCPAGLVKDACGCCLVCAKAENESCGGLSNIFGKCGHGLKCVFEGNPVITSGICKKSG*
Haztecus|176774	**MKAALIYVVIFTFVVATFA**KKTTECQESREKALKSKACITAIVPVCDENGDYAALQCQEGSKFCSCWRKDGTPIIQPSEKIKACECHRQKDEKSSKGLLGTFIPQCAEDGKFQKIQCWGSTGYCWCADPKTGRNTSVSIRGRPNC*
Haztecus|176784	**MKLTKMIREKTMKAALIYVVIFTFVVATFA**KKTTECQESREKALKSKACITAIVPVCDENGDYAALQCQEGSKFCSCWRKDGTPIIQPSEKIKACECHRQKDEKSSKGLLGTFIPQCAEDGMFQKIQCWSSTGYCWCADPKTGRNTTDSVRGRPNC*
Haztecus|176796	**MKLTKMIREKTMKAALIYVVIFTFVVATFA**KKTTECQESREKALKSKACITAIVPVCDENGDYAALQCQEGSKFCSCWRKDGTPIIQPSEKIKACECHRQKDEKSSKGLLGTFIPQCAEDGKFQKIQCWGSTGYCWCADPKTGRNTSVSIRGRPNC*
Haztecus|300057	**MGVKFYFIAFILNVVFLNTMA**LKCKECGTYECRPPPNNCPVGTVTDVCNCCLICGKAENEICGGEWNLLGKCGEGLKCVKKPGSNNQLIPPKAGICKKIDW*

Haztecus|339074	**LHCITPHCIIFIDINLTNVQHSPASLFIDSQTSTVFPKLYSYTEKATHQDMGRKLCLVVFVLLGIFTSTMA**FTCLACGSYECPPLPLCTAGVVKDVCDCCVECAKNENENCGGMWEQYGKCGKGLKCVSTGTTTPDPFMWDWPLPGICQKE*
Haztecus|352810	**MEIKFCFIGCVFFGIFINALA**LTCRDCNTFTCPPPPDNCPAGLVNSMCRCCLVCGKAEDEICGGDWNLFGTCGNGLTCVMTRFNNNSPRP
Haztecus|382099	**YSCLSSRSDKIKMKLFILVCFMLVTLSLA**EQTPCQAKREKILNQNLDVEVIPECDENGNYKAKQCKKNGVDCQCWRTDGTPINDFSPNLKACSCVRSRDDANRPHLIGNYKPQCEADGTYSLTQCWGSVGGCWCVDAEGRKLTDKHFP

Table S2. Putative venom sequences encoded by 74 transcripts of the *Hadrurus concolorus* transcriptome. In bold: Signal peptide. In italics: propeptide cut. Asterisk indicate stop condon.

	**Ascaris-type inhibitors**
Hconcolorous|008487	**CGDNALEMKLRWVILAALLIMLTEE**IKTPSDCNPETEEWQECGTACPLTCKNYNNRPKICTRQCIMECFCKDGLIRGYNNDCVIPENCEMTQ*
Hconcolorous|012894	FKVRTILKMKFNTVVSTFAILVFCSVLENTLGQRDRRCGLPTEEFLTCGTACPLTCDNYQRPPRDCILPCVRGCFCRRGLVRDTRRGGRCVRPSECRR*
Hconcolorous|028862	*VAKQGKTSHPNFIKSR*SQKHKSFQSFTILGMNFNTAFSTFAMFVFCSVLMNSAAQQDTTVAYLSEDAAKSSKLPESRCRRLGEEFTMCGTACPLTCDNYQRPPEACILLCVAGCFCKNGLVRDTQRDGRCIRPSECGK*
	**Calcins**
Hconcolorous|c28713	**MKTSRLTTIFIAVVITIICLNIHDVEA***REIEFNAGRVVR*SEKDCIKHLHLCRENKQCCSKKCTRRGTNPEKRCR
	**CAP Superfamily**
Hconcolorous|001850	**PRLFQMEKHLSIIFLLQFFCYFSVG**CKYEKLGIIHTMCVYSSGACSNGRLLHSGAISAEDKSIILMMHNRIRSYLATGNISGLPPAADMLVVEWDNELAAIAQRWADQCTNGHDELRNTEQYYVGQNVAHYWSYNYKDPVLKDKADWVFSIEHWSSEYEKFRFNPAHISPFKFNNKVGHFTQMIWGETRMIGCGYVFYVHPKQGYTKSYICNYAPGGNVIRGIMYKISPEGAKCSFPVLHPSREYQGLCEHGR*
Hconcolorous|002358	ANSDKKCSMAQKCVIIVLLIVSTGMISNAVMERPMEIMVKETAIKLSNEQKLHIVHLHNVYRRNATPSAANMVYVQWDEVLARTAQAWANLCVMKHGIPASPKYDKGGYGQNLYFGPSNSTYRAIWLWYNEFHNFHFRSVNCTGIECGHYIQMMWWETKFVGCALSQCGKSEFFFVCHYYPQFVNNTTPYLVGEPCSLCDLGYGGLCYDNLCVTKPACEKYGLDCKCKLKCHNCGRVNTSTCSCECVDGWDSLDCSSQCRDQDKQCGKKGGFSSILNCPMHKGTVKKKLCRKMCQACGTVNITNLSRTCCDGTICSPGYVIDLDNRPCSCKPLCPGPECYSSVGKIWKDESFSFAIGIFALLWKTWKV*
Hconcolorous|002447	**MTSMILPILALWIIIAGTSA**YYDCDEKYTNITLDHTMCKIANESCRFLRKGKTFAAQLLRMHNNIRNSIHRFVGKEYPLATNMELMNWDEELYKIARIHSLQCVEEPDCNLCHQIGDFPVEQNFAVKKFKKSEVDNNGPVKRFQTVIKEWAAELQLYDPSVVSNLTITDGLPVNWINILRATTLFVGCASMNFYSDESGIFKEVYICNYGPAKLTEGEEIYKTGDVSCSNCKDDGMCDTEFKRLCVPADFEINIRTTSSQNSTRYPWYYRNESYYVGNATNEENSSWEEVTETLMETSIVEENATFVVTEIVPSEETSMEEVIELLPSDETSMEEDIEFLPSTAPSKENTYLIKRKIRPLVSKMLKYSAKLPKGMLQKQFVTDMKRVIRHLI*
Hconcolorous|014412	**MNLVLAILCLQLSSLPYILC**EECSRTSYGKYDIALSHENSRDEKIFLTELKMSSGDEESPEEEITEMKECPELYQRYSTEHTYCKVSTCNIITEGVNEDDKELILKIHNELRNKLANGSEERYRQLPSAANMMELEWDDELARVAQAHASLCVFKHDKSAQRQVENFSVGQNLILSSGAQKD

	WNAAQNLYKEEVCFFFPEYIKPFHFEDNFGHFSQVTWANTWKIGCGFAAFRENGKTKSIYTCNYGPSGNVIGQTHYIVGEPCSKCPKNTECSEQYPALCKSKTEDGPQVERPSTEDYILFCDFSDDDPQECNDVKITGSRNLTTRHIYSGNYKTVVLERGESVSVDFGTAQNENGICPFLHIRFGPNNANDSTGSVLEISYSSNVIVPMPPTIIHPHGSSFFTAAVLINYGGPLKLSVKMRAEENAAPQYFDVKFWGIRKGSCKLFV*
Hconcolorous|014413	**VFGAIQNFTPVTMNLVLAILCLQLSSLPYILC**EECSRTSYGKYDIALSHENSRDEKIFLTELKMSSGDEESPEEEITEMKECPELYQRYSTEHTYCKVSTCNIITEGVNEDDKELILKIHNELRNKLANGSEERYRQLPSAANMMELEWDDELARVAQAHASLCVFKHDKSAQRQVENFSVGQNLILSSGAQKDWNAAQNLYKEEVCFFFPEYIKPFHFEDNFGHFSQVTWANTWKIGCGFAAFRENGKTKSIYTCNYGPSGNVIGQTHYIVGEPCSKCPKNTECSEQYPALCKSKTEDGPQVERPSTEDYILFCDFSDDDPQECNDVKITGSRNLTTRHIYSGNYKTVVLERGESVSVDFGTAQNENGICPFLHIRFGPNNANDSIGSVLEISYSSNVIVPMPPTIIHPHGSSFFTAAVLINYGGPLKLSVKMRAEENAAPQYFDVKFWGIRKGSCKLFV*
Hconcolorous|023377	*MESYLRSFVTLVVVSTQSQFHIATEIVSFRENSTYSWIHQATNRHFSKPSRWARQAVMMSESERQKVVHLHNLYRSMVIPSAANMEYMEWDHR*LAALAERWGQNCDWKHGNPPHKFPQGVGQNLYKGPSAYISTAMGLWFDEYKDYNYFNLKCKPKKMCGHYTQIVWATTRYVGCGIVNNCWPDYKYYIVCNYWPPGNYQNTKPYIEGKPCTRCNFTGNGLCSKNSCVNRDQCKRHKLDCACDLKCYNCGEFDKENCNCKCKDGWKSHDCTEPCVDSRRCEKHECWRYKLSRTNPCESTCGICKGVNQSNLRNTCCDGVLCPYGQVYHSGDRPCVCRILCPGPKCGAFLHGPYFVLLVVMILFSGYYNKL*
Hconcolorous|032202	**MIVQLLLSFALFSWDVVSS**QTCPEIYLRFSKDHTYCLRSNCHVIKRGVTEEDKKIILDIHNEFRNKIALGQETSPRQQPPAANMIQMEWDNELAKIAQAHSDQCIFEHDNAPQRQVENFPVGQNLLITMLSKTINWRKIRMWYTSEINYFYPQYRQPFTFATAYGHFSQMVWAKTWKVGCGVSVYYDNMDNMDKVLYTCNYGPAGNMRGDAIYSVGAPCSQCPKNTQCSNEYKGLCKSLTPDGPQKEISISSRDFLLYCNFSVNDSPGCRNVQISGSKPFQTKKLYSGEYKTAILNGGESITIKLGKAQDNRGICPFVYGSFGPNRDGDAKRSAVSIGFSAPRIMFGDPVKIEYGSSEFWTIGILMRFSGEMESTIKLQAYPGASPQYFNVKSFGIGRGKCPKF*
	**KTx**
Hconcolorous|028795	**MVKNFFAAFLIIMLISSLVDG**KSTVGQKLKKKLNQMFVKVKEVVNKSEYMCPLVSSYCKQHCARLGKSGECDLLECICS*
Hconcolorous|024751	**MNTKLVLMMLMITAVILVFEAETVIG**TGTPCKNPKQCAGPCQAKGCKHGKCMNGKCHCMLCKRS*
Hconcolorous|024749	**MNTKLVLMMLMITAVILLFEAETVSG**TGTPCRTPKHCAEPCKAKGCKHGKCMNGKCHCMLCKRS*
	**Scorpines**
Hconcolorous|008061	**MEIKLTILILLVITSFCSC**GILREKYAHKAIDVLTPMIGVPVVSGIVNNAAKQLVHKIAKNQQLCMFNKDVAGWCEKSCQESVHQKGYCHGTKCKC
Hconcolorous|029426	**MQLKFIIFFLLGMATFCTC**GILKEKHFHKAVDYLVPAVVPDVVKNLAKQAIHKIAKSESFCMFGKDMRKLCNQSCMETLNMKGNCHGTKCKCEP*
Hconcolorous|033550	**MNTKLTILCFLGIITIVSC**GWINEKKVQEALDKKLSDGVMKSMAKAIVHKVAKNQFGCFANVDVKGDCKRHCKSEDKEGICHGTKCKCGVPISYL*
	**Kunitz-type inhibitors**
Hconcolorous|102082	**MIIFYSLFSILVLTSINVAEA***GHHNR*VNCLLPPKTGPCKGSFARYYFDIETRSCKAFVYGGCQGNSNNFSKKHHCEKQCRAFRYFGGK
	**La1-like peptides**
Hconcolorous|010016	**MKHLHLYLVFGCLSLCALLSMSVGAG**EYCQVGEMSIPVGKQKQDSRECILHKCVNQNNRIVLDSFSCAPQEAKRGCRNVPGPVDAPFPDCCPISVCRGKQWDD*
Hconcolorous|031288	**MAHALTPILFGILIMFSISSLSTG**YGESCQAGKYTIHVGRSVQDSKSCILYKCINYNRRYSLETLTCAKMTLKSGCRYVPGPATARFPNCCPMVVCRGSG*
	**NaTx**

Hconcolorous∣028874	**MMERRFVFILFLAAFIYEIRNVEG**KDGYPLTVKGLKYSCVAGMVIGDNRFCESICYDEGSTYGYCYGFGCYCEGMRDDVKVWGE
	**Phospholipases**
Hconcolorous∣000043	**MWTTTAILFLSAFLVAES**GIFDIVDKVLPITTTFYREKNGHRMVEIIEVNTYIGGKKLVDCYLYGHLYIIDKMMELVPSDIVKYVNKKEMSKLVNTCSDLHVKNLREEVFNIIKTPFDFARKLFKSLLIFPGTKWCGAGDVADDYDDLGIYEKTDKCCRTHDHCNDSIVGFETKYDLKNKDFYTKSSCSCDLRFHSCLYKKEAIHSDAVGHLFFNILQTQCFKDEYPIVKCLKKWGIPLIRETCQKYKLDYNGTKKYQFFDAKMYKGKNESPFLKKLLSH*
Hconcolorous∣003898	PAGKTKYGLKNEGTYTMMNCKCEEAFDKCLSDIPGYFTQKAVSMVRYTYFELYGN
Hconcolorous∣004391	**MAFLCLTSLLILISLRNA***RIIQRELYVNFEPVSNQKDSWPVARAAIVNFDEGFETGR*EFSECRMLNSIQEIARETVNFPQHTIKRVSKEEMDVLERTCSRPLETERFFIYKGTKWCGPGNIAENEFDLGILQADKCCYAHDHCDSIAAGETKYGLVNNGYYTLLNCDCEESFDRCLKATADKVEGSEKEDTLKIRHIYFNTIKSKCYRLYCRNRRSGTDNTCINKTALWKESYHEF*
Hconcolorous∣005385	**MVKKKCLVSLVICFALTTPPSSS***KDGETLIVNQLSIPQDTHSPTTNSRNKHSEGHKGAKKGVDLASNRPSRIRR*SIMQLAGMLRCTTGCDPLAYKSYGCYCGYLGAGRPVDDIDSCCREHDRCYSNTICPQLFLYFFRYPWRCTSLGIPQCGRMPGITIAQQCARQLCLCDREFVRCIGRFPCPRQSAICRTNPLGVLQSLIVGKR*
Hconcolorous∣007421	MKRQKSALFEMMNLSLSGCGFLCIYHVGVASCFREYAPHVLVDKIAGASGGSLAACALICSVPLGETTSDVLKIALQARSRTLGPLHPGFDLNKILHEGLVRMLPEDAHLRCNGRLHISVTRVKDFKNVLLSEFNSRDDLIQALLCSCFIPFYSGIVPPKFCGVAYVDGGFSDNLPVLDDHTITVSPFAGESDICPQDTSFNILQFSMSNTSISISAGNLYRFVSTLFPPHPEVLSQMCQQGFDDALKFLQRNNIISCTRCLAVQSSFGIAESGITQQTDTKEIDHPDDDCIDCRYRRQMALLDSLPEAVVKAIEDCCDQMNKGVINWLFRHNPVKILPFFTLPYVLPIDITIVIFAKIWETLPYVQREMKSSLSEFLTFIRNLITSFDKKSQYSAKFSCQLAITEFDYANKEKKSTVAPVIKVLQSSPDGESTAAKRQLKKRMSYAGCANISRQLPMRRKSMVETSSPERVIKNMKVDFTVDLSETNMVSENKKKKVIDAFQSLKENDDTNVFDIANKVLELEKDYIEYIEPQKSDFVEALEVTNTNEAVMAYFYKEGKKVKVTEIFNISEEDSSIAMTDDEKELNTNLQWDSDWDLVSSSLPDYVPTIEDEQDLFPLEEYTGPTTSAFGTVDAHGLDTSGNEQRRSRKKSVISKLPFTCMEK*
Hconcolorous∣007422	MKRQKSALFEMMNLSLSGCGFLCIYHVGVASCFREYAPHVLVDKIAGASGGSLAACALICSVPLGETTSDVLKIALQARSRTLGPLHPGFDLNKILHEGLVRMLPEDAHLRCNGRLHISVTRVKDFKNVLLSEFNSRDDLIQALLCSCFIPFYSGIVPPKFCGVAYVDGGFSDNLPVLDDHTITVSPFAGESDICPQDTSFNILQFSMSNTSISISAGNLYRFVSTLFPPHPEVLSQMCQQGFDDALKFLQRNNIISCTRCLAVQSSFGIAESGITQQTDTKEIDHPDDDCIDCRYRRQMALLDSLPEAVVKAIEDCCDQMNKGVINWLFRHNPVKILPFFTLPYVLPIDITIVIFAKIWETLPYVQREMKSSLSEFLTFIRNLITSFDKKSQYSAKFSCQLAITEFDYANKEKKSTVAPVIKVLQSSPDGESTAAKRQLKKRMSYAGCANISRQLPMRRKSMVETSSPERVIKNMKVDFTVDLSETNMVSF*
Hconcolorous∣009441	**MLLLKLTVFLLLFVQPAPS***TDLPLPLPHEKKLTAFYQEEQNPYMLIIGR*TGKVIHCHQYEDKKEADRVLAALHLDDVEKIEHRRMESLTKFCAKEKHKFIPKTYTKKVFIYPGTKWCGMGDNAANEDELGKEKEADSCCRDHDHCKDSIPAFSIKHNLTNYSPFTKSHCDCDKQFHTCLAKARTKTATIISNLYFNLLNMECFEHKTCSSNETCITWQWKLAKSDGFAIQ*
Hconcolorous∣010247	**MNCKLLMLTMLAALSGCHQ***TNDFVSWTVLEDNEKEMR*VRIGHIVALQTSVDWETGLNSTHNGLVLRQVTDGKHLIQLIYDSSWTLLDCEYLKHPEYVKKFLNKFVRDFECVRILNSGQRSPDCNNSTYRLLKDGVPPDLRDLLNYRSLRSDCRQLHIAIRKEVKRRRQIDYDRYKRDLFLFPGTNWCGFGNSAKKFNELGYNAATDRCCRDHDLCPYTVESFSHNYGFFNYRFHTISHCDCDERFRACLKLANTAISNLVGKLYFNIVQTKCFVFKTEEVCLKRSWWGKCMKSKRQKNAYPRDCLSY*
Hconcolorous∣014187	**MSSIILLVGLLSLVNLALS***TENELYLNFEPLTSQRDGWPVARAVRVQFSNRFEEGRESRR*MEGCQILESLNDIAREALHTPRHAMKRISKEEMEFFEGRCLSVGESERTIWGTKWCGAGNEAANYSDLGLFDNVDRCCREHDHCDNIPAGKTKYGLKNEGVFTMMNCKCEEKFGKCLDDIDGIISSKPVSAVKYVYFELYGNGCYNVKCENGRSTSGECANGVAEYTGENGVVAKFINLFGD*

Hconcolorous|019239	**MNFWCTLVLVTLLTVCCRINECRS***LHRKPRSLPQLARMVEATTGR*KATDFVPYGNWCGIGGSGKVVDPVDKCCQTHDNCYTRASENGICKAIVSLYIGKYTWKFEKGEIICSTPEDSNECDAASCICDKEVAMCLAKNIGSYQKDHRFVRSLFKS*
Hconcolorous|021750	MTASPQPQIKTVHQLQNMLALFWKSVNSNYNKMPSSYVTGLFKTKQWKNAVKNCKISEFAKIVGIMQFHWKFPILLQSNINKNTENLPILKSDTQHAPVTKKTEPTQILELDKALTDWINDDKKPDNNLSFQDNRNSLDIAKSKPTSSVLGNLEKVLSSWFQGDALTFGFQTSKNWMMAQKDESSLTTTYKVTKINVESRSRFLVKSLSSASSETSILYRLQEMGKHLLRYPDQKDIMVKENAIPVTLRIRESFSNTIQAKAQEILSLLGYSDPPGSAGVRILSIDGGGIRGMIAIEILRYLENVSGKPVYQLFDYICGVSTGAILSMLVGGLHLPLDECERLYVKMSSELFKRSAFLGTSGLIWSQSYYDTSMWVNMLRKTYGEIMLSETCNKKGNPKISCVSAVMNQPILEAYAFRNYSLPYRKQSLYPGSSNHKLWQAIRASAAAPGYFEQYTLAGIVHQDGGLIMNNPTPLAVHEAKLLWPKENIQCVLSVGNGRFIPSPEKMAASAGLTTVMMKVIDSATDTESFHAVLQDHLPSNVYFRINPYMTERVSLDEIRTEKLNLLKQDAKKYISWNEDKIQQAVQALTKPRSHLQKFGDWIKLWSLKHQKKSL*
Hconcolorous|023760	*MNFLEIGPLSFINDLGEKGREGLVLKR*SGGHFTKPVCLKLRRAVTDCCGFWRKRWLVSKDNFVAYIRPKDGIIKSVLLMDSAFTVDCGLTATGVHHGLLISNMCRQLLVKCWTRRKAREWMQHMTETANTLARDYTQQNRHGSFAPVRDSVEARWFIDGGTYFEAVADALEKAKVEIFIADWWLSPEIYLKRPVIQGELWRLDRVLKRKAEEGVKIFVLLYKEVELALGINSYYSKRQLAQLHPNIKVLRHPDHVTGGVLLWAHHEKIVVVDQIYAFLGGIDLCYGRWDDYLHRLTDLGGIFKTSQNTKTSEYQLLPRRCCSTSDLSEVSHLENCQKIIIQAKQMPKIEIHKHHSAASLPASHDTGIAISDDSQQPETNVESRHVPSTDLLYTIEDERIISKKDKDTSDGIMPSRPRFTSKLRTQRVMQAVTRFQALRHKLQHKDYSQDSLKMGALSDNLGLPTTELRRTASEIALHQMGLEGSCKLWFGKDYSNFIMKDFVHLHQPYQDLVDRTTTPRMPWHDIGILVQGLAAKDVARHFIQRWNFTKMEKAKSYGSYPWLIPKSTGSFTHISPLPKSSTGVIFCTNCQILRSVSTWSSGIRNTEKSIHAAYIDAIKNAKHFIYIENQFFITQAAGNKDVFNEIGEALYQRIIQAYKNNEMFRVYVVMPLLPAFEGEIGTNTGTAIQAITHWNYASICRGGSSLLHRLAQEIEDPVSYISFYGLRNYSMLNDK
Hconcolorous|023761	*MSASGGGFAVTHPSEADSDYEDLRPPDSEDDEIDDGAHKDGQPYIPYSKIHETPFGFEDLRQQLLVPNYPINLRIVDAQRLFGTSVMNPNLYIIKLQHGNFEWTIKKRYKHFQRLHQQLLLFRASLSFPLPGRRYRERRKSYRSKDKKSLPQFPRRPEPLIQQEHLPHRAKQLEEYLRKLLRIPLYRSHYETMNFLEIGPLSFINDLGEKGREGLVLKR*SGGHFTKPVCLKLRRAVTDCCGFWRKRWLVSKDNFVAYIRPKDGIIKSVLLMDSAFTVDCGLTATGVHHGLLISNMCRQLLVKCWTRRKAREWMQHMTETANTLARDYTQQNRHGSFAPVRDSVEARWFIDGGTYFEAVADALEKAKVEIFIADWWLSPEIYLKRPVIQGELWRLDRVLKRKAEEGVKIFVLLYKEVELALGINSYYSKRQLAQLHPNIKVLRHPDHVTGGVLLWAHHEKIVVVDQIYAFLGGIDLCYGRWDDYLHRLTDLGGIFKTSQNTKTSEYQLLPRRCCSTSDLSEVSHLENCQKIIIQAKQMPKIEIHKHHSAASLPASHDTGIAISDDSQQPETNVESRHVPSTDLLYTIEDERIISKKDKDTSDGIMPSRPRFTSKLRTQRVMQAVTRFQALRHKLQHKDYSQDSLKMGALSDNLGLPTTELRRTASEIALHQMGLEGSCKLWFGKDYSNFIMKDFVHLHQPYQDLVDRTTTPRMPWHDIGILVQGLAAKDVARHFIQRWNFTKMEKAKSYGSYPWLIPKSTGSFTHISPLPKSSTGVIFCTNCQILRSVSTWSSGIRNTEKSIHAAYIDAIKNAKHFIYIENQFFITQAAGNKDVFNEIGEALYQRIIQAYKNNEMFRVYVVMPLLPAFEGEIGTNTGTAIQAITHWNYASICRGGSSLLHRLAQEIEDPVSYISFYGLRNYSMLNDK
Hconcolorous|023762	*MLQVTELVYVHSKLMIVDDRIVIIGSANINDRSMIGKRDSEIAAVVEDVDFEKSLMNEIPYDAGAFAGSLRR*SLFREHLGLMSKDHESCDIRDPISEHFFKEVWMKTAETNTAIYEKVFRCIPTDNVHTFSKLRQYLSQSVMAKTEPEIASKFLQQVKGYLVLLPLNFLCSENLTPAAGTKEALMPVSLWT*
Hconcolorous|023763	*PLSDKASLASDLVDRTTTPRMPWHDIGILVQGLAAKDVA*HFIQRWNFTKMEKAKSYGSYPWLIPKSTGSFTHISPLPKSSTGVIFCTNCQILRSVSTWSSGIRNTEKSIHAAYIDAIKNAKHFIYIENQFFITQAAGNKDVFNEIGEALYQRIIQAYKNNEMFRVYVVMPLLPAFEGEIGTNTGTAIQAITHWNYASICRGGSSLLHRLAQEIEDPVSYISFYGLRNYSMLNDKLVTELVYVHSKLMIVDDRIVIIGSANINDRSMIGKRDSEIAAVVEDVDFEKSLMNEIPYDAGAFAGSLRRSLFREHLGLMSKDHESCDIRDPISEHFFKEVWMKTAETNTAIYEKVFRCIPTDNVHTFSKLRQYLSQSVMAKTEPEIASKFLQQVKGYLVLLPLNFLCSENLTPAAGTKEALMPVSLWT*

Hconcolorous|026891	MLKKTELTKRKWNGWIKPSCIPITIIFILIVLVVMLPLLDQKHDDKVHLLTEPSNCTEPCWATLVESIPENMTYNISLNHPSTFEGWLNLIQLAEHTIDIAAFYWTLRGGDVISDPSDWQGEKIFDDLLAAGTERKIKISIVQNLASHRFPNNDTKELEEKGAAAVRNINFKKLVGKGILHTKMLIVDDKHFYVGSANMDWRALTQVKELGIIVYNCSCLAKDVKKIFDAYWLLSETEHIPLPWPPYFDTTFNKTHPAVLTINNTSTYAYSSSSPPQFCAKGRTSDINSILSVIHKAKEFIHVAVMDYFPALIYTKHLKYWPVIDNALREAAITRRIKVKVLASYWKNTRPVMQPFLRSLQSLNSTDISIETKLFVVPIYSPSQAKIPYARVNHNKYMVTDNSAYIGTSNWSGDYFISTGGVGFILENTDNSTNSSIQIQLQDIFERDWFSEYAYPITEIDIR*
Hconcolorous|027415	**MNILFIAVFLNIITNFDVKS**QSILSGVQKALDSVSNFIDDISFGIKQVRDGLSTVDDIVNYAQGKPCEYKCPPGLKLKKNYYYKPIPQGCGAYGIQVVLSLPLLKDTEKCCDKHDICYSTCMTNKTHCDAEFGTCLYKKCEKQAKKLGEDVRKCTGVSKLFHLGVQSLGCNAFKAAQAEACRCASKDEL*
Hconcolorous|032907	*DIISKAKR*YIHISVMDYMPTTCHNQNLRYWSILDGALRQAAIDRRVSIKILVSDWNETPPSMFYFLKSLLALNSTLIHIDVKLFIMP
Hconcolorous|036712	NERGIRVRIVRNHPFTSSPRPYGDEFLDSENVQTQNINLRKLQGAGLVPGTQIWIVDNTHLYIGSSALDWMMVNQMKDIGIAMYNC
Hconcolorous|036713	AMYNCSCVAEDLQKIFQLYWMLTGTDSSIPKDWPITLETTINKEVPLNMEINDTYTQLYISNSPEILCPEGRSSNIDSILDIISKAKQIGR
	**Hyaluronidases**
Hconcolorous|007008	MLWLVRTIAVIIFIFGGKANFNVYWNVPSALCSKKYGINVTDDLMKHRILVNNGEQFIGDKIVIFYENKLGKYPYIDTKKGIYVNGGIPQLANLTEHLKAAEKDIDNMIPDPNFDGIGIIDWESWSPIYDYNWNKKNIYRTRSIELVKKITPPFPAETIKRIAKKQWEEIAKQWMLQTLELAKKKRPKARWCYYLFPDCYNYKEKQTQSEFACRESVRTNNDRLSWLWEASTALCPSIYFYNKHVNYSYHQRLWYLHGRLSEAVRVSSGKALIYPFLNYLCKDKDKNILFDVPEKQFWQMVSFTASMSLDGVVIWGSSSYVKRKENCEALASSVKKVIGPSSLTVSSNFNHCSVTICSGAGRCFWPDKQFISLTYMSKDPPRLQPEEIFCRCKKNLGRYCNVSNLDPHHFIEDCSAEVDQQ*
Hconcolorous|007009	*MLMEEYRRQWMLQTLELAKKKRPKAR*WCYYLFPDCYNYKEKQTQSEFACRESVRTNNDRLSWLWEASTALCPSIYFYNKHVNYSYHQRLWYLHGRLSEAVRVSSGKALIYPFLNYLCKDKDKNILFDVPEKQFWQMVSFTASMSLDGVVIWGSSSYVKRKENCEALASSVKKVIGPSSLTVSSNFNHCSVTICSGAGRCFWPDKQFISLTYMSKDPPRLQPEEIFCRCKKNLGRYCNVSNLDPHHFIEDCSAEVDQQ*
Hconcolorous|021774	**MLFFVCIVSIFNNIEA**SFDVFWNVPSSLCSIKYDVNMTETLLKYNILVNDGESFTGDKITLIYENGIGKYPHIDPNKGDINGGLPRLDKLKEHLNLAEKDIQKIIPNPAFTGLGIIDWEAWRPIWEYHWGGLSIYQKRTIDLVKKDHPTESDQFIQTTAKNLWENTAKQWMLKTLELAKKLRPQGQWCYYLFPDCYNYFGKDQPSEYFCSAMIQNNNDRLSWLWDASTALCPSIYFIENQMKYNESQRTWFLYGKLAEAARVARPSTKIYPYINYMVHVSQIPVPRDHFWKMLSLIASMGFDGAVIWGSSSYLGSKKSCDDLEAYIENVIGPAVTTISSNVNRCAQEICNGRGRCTWPNEPFISWQYLIDTNGPNFDSQKITCKCQSHSGRYCN*
Hconcolorous|030771	**MNSYILIGIILNFIAHVIEA**SFKVYWNVPSSLCSVKFGINVTETLINHDVLVNYDEHFLGEKLVIFYENKLGKYPYIDPKYGDVNGGLPQLGNLEKHLEAAEKDIQTIISNPNFDGLGIIDWEKWRPIWDFNWGKMRIYKTRTMELMQKKHPSWPWKLIENASRNQWEETAKQWMLKTLELTKKIRPEGRWCFYHLPDCYNYAGKDKPEQFLCSSLVRKHNDRLIWLWNATTALCPSIYFDERQTKYNESQQIWFLYGRLSEVLRVSQPRTPIYPYINYRIHSSLEEVPKERFWLMLGHLASLGLDGAVIWGSSNYVKTEEECRNLELYVKEVIGPASSTISSNVNRCSEVICNGEGMCTWPHQSYTSWKYLTNQNASAFKREDITCRCQMYKGRYCDLYHSNSTDLF*
Hconcolorous|033175	IPDPNFDGLGVIDWESWRPIYNYNWGTMTIYKTRTVELVRKENPSLKVDLIQSIAEKQWEEIA
Hconcolorous|033907	GGNCKEHFWRMLSLSASMGLDGIVIWGSSNYVRKKEDCEALASYVKKVIGPSSLTVSTNFNFCSKTICRGQGRCVWPDEPYTSWRYMCNRDLSDFQPGEIICRCKKKERQIL*
	**Metalloproteases**
Hconcolorous|004160	**MAVRSMCPFTTLSLLLLSFGSKQSVS***QDRR*PNTGLVFPEANTQYCRTRDGSAGSCVQISECKHDIDYQRGILPELCYWDNSRPIVCCLRNDRTETPVTVPDRDRVTSVTGCGKRTIPKDASRSPQIAGGRISLPSAWPWMISIHRSNFGIESFLCGGTMVSVRYILTAAHCFGRNGNDRRKIPTSRFVIRVGSNINEEGVAHRIKNIIVHEDYKVGQHYNDLAVIEVTELIKLSPMVQPICLPSSEMQGRQLVGRDVTVIGWGDQSFGGIRDRK

	LREVNISVIDRQQCDESYSALSSLAIPRGITSQFLCAGDVKGGKDACQADSGGPLMMHSSDWTIVGIVSFGYGCAQKGYPGVYTQVVSYL QWIKDNTRM*
Hconcolorous|006453	IISLRKLPRSSESSTCQSHICKKTGEKLKSWINSNIDPCEDFYNHCCGGWLKENPLPNDTEIYSVFNKLESEISEYIKQLLEESNYKQSPRFINQ TLMFYKACLHKENIEAGKAKSLLSFLEELGGWPLLTNDWKGEDYDWVEVISKLVWKTASGYLIRFIISPDVKNTSNTIIQLDRPSLMMNA DELLNPNGTSD
Hconcolorous|012106	*IVKFITAR*PMGDKQEGSNKANPGVVGIAYVGGVCDRDYKCGVSEDNGLDFDGSSTYAHEVAHLVGCPHDGDPPVSYLPGSPGAKSCSW DLGYIMSYVRKNEKQFAFSSCSEECVSHLATLSGSECLFRQDVTNSLNNQENRLPGDFFSDTIGTTDMKSIYEGKCKIKRKDFTLYKIPNNS CDYSCKTPTVNNRFYYWIFQCSDGDICDTNKVCING
Hconcolorous|018739	*MGNISSSQRLALNVERTSSKR*LYFPIWPGGRIPYEIDQQSVCPIPNIRDAMREIEFYTKCIQFVPKRNEVDYLYITTSKNQISSKGCSSQYVGRL GGKQTLILNLECCDRLIYLHELCHAIGLHHEHQRWIRDHYITIDFCNIIPGHHHNFDKLPPDDLGDFDFDSIMMYEPYAFSLDECKPVIKPL DQKMRIREMREKTSLSRGDICKIRRLYK*
Hconcolorous|018740	*MGNISSSQRLALNVERTSSKR*LYFPIWPGGRIPYEIDQQSVCPIPNIRDAMREIEFYTKCIQFVPKRNEVDYLYITTSKNQISKGCSSQYVGRLG GKQTLILNLECCDRLIYLHELCHAIGLHHEHQRWIRDHYITIDFCNIIPGHHHNFDKLPPDDLGDFDFDSIMMYEPYAFSLDECKPVIKPLD QKMRIREMREKTSLSRGDICKIRRLYK*
Hconcolorous|019720	**MLWYRCFLFLLAHFNCTVWA***LHKLRDAEAQEAIPILTDSRGQPLPIER*FDLYSKLFFKFVAFNFTFFIELYPANEFISPTFHVNNIETVGNST NSRMQQHKSFKNCYFRGRIVDEVNSLVSVSLCQGMLGFFRTSRGDYMIEPSIGRLSSVHFIKSVPVPNGNKTTQICEARDSFHDTMLNRA MHRHPPGRRRKRSVSVERNLEVMVVADSKMARYHKSNLRHYILTLMSTVALIYKDPSIGNDINIVVVKLVVMDESEDIDIIFPSASKTLRN FCRWQQQYNDYDDSSPHHHDTAVLLTREDLCRLAKTCDTLGLAQSGMVCNHKSSCAIVEDNGLSAAFTIAHELGHVLSIPHDDDHKC NRFQGDGQRLHVMARMLDYNSHPWSWSPCSRHYITAFLDSGKGHCLLDKPITNEIQSPEHETHQPGQLYDMDHQCELVFGKGSKICPY MPVCKRLWCTMEDLTQGGCRTQHMPWADGTLCGREKWCQHGECIRMRAATQYPVDGQWGKWQSFGQCSRSCGGGVKRSERYCDN PSPAGGGKYCVGKRVRYKSCSSQECPNGIQDFREEQCSAFNGNSDFLDSRTNVKWTPQYDGIHMDERCKLYCRAVGTSAYFPLKDKVID GTPCGVDTFDMCVNGKCLPAGCDHRLDSKKKLDICGICGGDNSTCKIVAGHFNKVEYGYNHVVLIPAGASNLDIRQFGYQNSNDDNYL ALKDSSGRYLLNGDFMVSMFPKTIQYGGAMLEYSGSDKIVERINTTKQLKKDLIIMVLTVGKLRSPDVRFQYTVSLENAQFYEWDIEKKWS TCSHACRGEQYRDPICVDIYSRRVVNDAHCYSNQKPPRLAQVCNLQCSLSWQILKKSDCSSNCGKGTRHRIFKCVQQSGEDSQLLEDSFC EHLESKPQEIEECEGPCSNIQWEFGEWSECSQTCGEGIQTRSAICIDFQNEKQNDALCDASAKITTKSCNLSICPEWEVGDWTQCSVTCGIG ERHRPYWCRYADQVVSVTFCNRETVPIHKEKCNMGRCLQWNYGNWGPCSKSCGQGYSKRVVHCQLTNGTVVDDDMCDVDTKPTSA KECNLPSCPTTATPSSTFYTTDTTDSNFLPPIPLALVQDDMPKSATEKTVHTSTTDMPETAKWKSGSWSECSVTCGRGTRKRHLVCHDQV KHQIVSDSECDILLQPPILEVCEVQDCGQWEIGDWSQCPVTCGEGLSIRTVVCIKLNGEKGENHECDQSTRPSTERLCSMKPCEILPIGVPE MPIISHNSVEGIFYWRTGHWGECSKTCGEGKKRRQVACYDEAGHISSQCSSHQKPDEISTCNREPCPDWVVSDWSVCSHTCGGGIQIRSV HCQSSLRTFADEYCDSSMKPFTRQECNSQQCTINLNQYRWEKEEWSQCNVSCGTGFMHRHVYCIDSQHFVVSDKRCTTKRPRVRRKCK GTRCHYVWKTEDWSTCSEVCGKGTMKRKISCHQLNLYGWMDPTPIPQPLGNREHWCDVNNRPSDTKSCNLGKCEKNASWKVGPWN RCSHDCGKGKQRRRVQCFNLQGRKISFRKCERDLRPRRKRNCFIRSCAPISCSELYHRNRIRENGEYKLQVRGRPVQIYCAGMNTTSHRH YISLVSGEGDNYSEIYDKRLVKPETCPHGGARQENCNNECVDAQEGAGLTTFFRIGINLTSLQVLTHDFAFSQTHHGQQVPYGESGDCYS KKNCPQGRFSINLMGTGFVVSQKTTWVKQGNEPDAKVQRLKHGQVVQGKCGGYCGKCGPDSNVGLLLDIAPP*
Hconcolorous|023528	**MRWYQVIVVISTLFVQHVVWT***ESRTVSGEDPNEMEGEIVYPRVYHMSRKKRDVGSSDETR*LIVIKADNTTFYLELKPNDNLVIDEIDYND TSDPCIFQGKILSHPGGMAAISTCEGGGNMNGLLITPEESLLLQPLSKFKPHHVLHPIEKDIVAHVLFKAEDGVEEFCGTDHLGNNTYHLV PEEENEIPKENSRVKRSEKAVYTIETAVFVDKYLYNRYSEHSPRRTSQYAREMVFTIMNQVQLIYKYKSMKTNINIVIVKLEILPNSFQESVQS SEGDIDVYLDEWCTWQGKRTDRVWDHAVLLTGLDLFKMQNGSKNKKVLGLAWVNGMCRPKYSCTLNEGMNFEAAFVIAHEMGHCL

	AMLHDGTGNTCDDNTYIMSPKTGPGKTHWSSCSNKYLADFIKSGYAPCLEDGGKPVSSDLDLQNPPSLPGERISVNEQCKLALGPEYKVYEKSSSPYNDICRELWCVKGLWATSAHPALEGSKCGRNKKCLEGQCQGRTMKQTNQARYSSNMIQTATLFQQMLDKMRMLSREFLRFLHLNYT*
Hconcolorous|023662	**MIAKCLILFHLMDQLVC***LSLSELTFDKKQGDYVNGLKHYEVIVPQKVNENGLLIDNDLTHYYNFRSKRSTRISPLHYRLITTEHDMFLSLHPNHAAVSPGYVVER*YLGIDNGTIRLTSKELKNKHCYYRGDVRNLSGSSVALSTCNGLMGVIRVKGEDYFIEPVKGHPLNGTSRHLHLLYKRSAVSNGPYEYLPKEGKCGNKDDIGQAITKRAQWEKKRESGRRKKRKKRSVSLERNVETLVVADRKMVEYYLDDDIETYILTVMNVVSSLYHDASIGNAVNIIVVRLILLENEENKKEDPEISHDADNTLKSFCKWQRYINYKDETHPNHHDVAILLTRYNICTKINEPCSTLGLAEVAGMCQPHRSCNVNEDTGLGLAYTIAHEMGHNFGMSHDGPHNGCQALLGERQHVMSPHLNSDASPFIWSNCSRLEITKFLDRDWGSCLDDEPSDLNFNIPELPPGTMYNADHQCRLQYGPDAEFCEGIEDICQTLWCRQDNRCVTRLEPAADGTLCDRNMWCYMGKCVPVGERPTSINGEWGPWSSWTECSRSCDAGVMHSERHCNNPMPANGGRFCIGERKRYRICKSEPCLDDALSFRSVQCSKFDSIPYKEELHTWLPISTPLTPCQLHCKPKGKFFSVMLSDTVEDGTPCNPGTNDMCINGKCRKVACDWIIDSTAQEDRCGICHGDGTLCKTIRGKFLQRRGIGYIEIVKVPKGARNIRVEELGDATNYIAIQDQHGEFQLNGQWFIQWSGEYIAAGTIFYYHREGEKEELHAPGPTKEVVRILLLFQTENPGLTYDYTIPNKNVTRKPEFHWEYTDWSVCSSTCNGGIQVSRAKCFEKEAGLVENSYCNESIKPPDRTQICNRHECPARWWSGPWQHCSVSCGDNGIRRRTVICIRSLGPEEQVALPDEYCDMNIRPPPQQSCHHTHPCLMDARWETGNWTDNCDEDPCSYQTRHVYCNIPNGHCHEKDKPVSIRQCNNITCGVWTVGNWSECSQSCGEGHQQRDITCVGGSACHRATQPPHTQICNPTRCSSLSDFQISDNTIEETHKDSDIKVSIHKHRHHHGDNKNKNSKEISQRSHDVAKDIVAYPIYEVPDNKYKDFDIDVIHPIKKEPIIVAQHNNHHTELEFRKYEWKVGMWSECSAPCGGGIIKREVLCFDTITGHMVVTDLCDPFQMPNNEDSCNMDLCAEWIHSEWNECSSSCGQGWQHREVYCPRKYKCNYHSKPIESRPCSVKPCEQWIAGPWSQCSVTCGEGYQTRHVKCVNLKTQALVMDCSNEDRPKHYQVCRNEECREEKSEFNQCYDKLEVSTCRSLPHMCNTWYFKAKCCQTCNRLEISRRRIRKSKDET*
Hconcolorous|029707	MVMVWNEGIIRYTFDDKLSDSIQEMVTFAMRKITNCTNCIKFFYRKHARDYLFITEGERCSSHVGRLGGKQELYLDTNHIALSVILHELCHAIGLYHEHQRSIRDDYIKVYYENIKEEHRMHFIRMSREDDIEGFGFDFYSIMLYGNH
	**HDPs**
Hconcolorous|004367	**MNAKILLVVFMITMFVTEQVEG***KFSFSWIKR*IWKSKIGKAARKAAGNYVAKKLENAAAPAEGGPKRFDEFMDSLYY*
Hconcolorous|021934	**MNAKVFLAIFMIALLVTDQAEA**GWWKALKSIGKKVWKSKLAKDIKNMAKQRAKEYIVKKLNPPPEEEVAAIDALMNSLDY*
Hconcolorous|027436	*LTPFLFR*SIVNLQPSVHKKMNAKVFLVVFMIALFVTEKAEAGILDTIKSIASKVWNSKTVQDLKRKGVNWIANKLGVSPQVAASMTLDEIMDAFENY*
	**Other inhibitors**
Hconcolorous|002969	**MKTALIYIVILTFVVATFA**QKKTECQESREKALKSNARIKAIVPVCDENGDYAALQCHEGSKFCSCWRKDGTPITQPSGKIKACECHRQKDEKSSKGLLGAFIPQCAEDGKFQKIQCWSSTGYCWCADPDTGRNTTASARGTLNC*
Hconcolorous|009579	**MKILLGLFVLTVTVSC**QPQSGGIGCKPYEEYREAWCEVTCDNVRRVTCLTAEKRPGCYCKIGTIRDEDGQCISTEACSKRVCTKKNQRLDMSGCFTVCTGLGTSYFGCPFVQQPSCNCKRGFAVARGIRGDCIPVSSCRKPNWGN*
Hconcolorous|010515	**MKLFILVCFMLVLVTLSLA**EQTPCQEKREKILSQNLDVEVIPECEENGSYKAKQCKKNGVDCQCWRTDGTPINDFSPNLKACSCIRSKDNANRPHLIGNYKPQCEADGTYSLTQCWGSVGGCWCVDAEGRKLPNKHFPVDC*
Hconcolorous|010516	**MISKCLRSDKIKMKLFILVCFMLVLVTLSLA**EQTPCQEKREKILSQNLDVEVIPECEENGSYKAKQCKKNGVDCQCWRTDGTPINDFSPNLKACSCIRSKDNANRPHLIGNYKPQCEADGTYSLTQCWGSVGGCWCVDAEGRKLPNKHFPVDC*
	**Other venom components**
Hconcolorous|002969	**MKTALIYIVILTFVVATFA**QKKTECQESREKALKSNARIKAIVPVCDENGDYAALQCHEGSKFCSCWRKDGTPITQPSGKIKACECHRQKDEKSSKGLLGAFIPQCAEDGKFQKIQCWSSTGYCWCADPDTGRNTTASARGTLNC*

Hconcolorous|002972	**MKTALIYIVILTFVVATFA**QKKTECQESREKALKSNARIKAIVPVCDENGDYAALQCHEGSKFCSCWRKDGTPITQPSGKIKACECHRQKDEKSSKGLLGAFIPQCAEDGKFQKIQCWSSTGYCWCADPDTGRNTTASARGTLNC*
Hconcolorous|009196	**MFRLILFFISVASVYS**LSCPCWREPDKTKYCPPPPTNCPLGLTTGPCGCCLQCYKDNGEACGGPWRIIGKCGKGLRCVKETNVGEPKRYYINQMEGVCKPIDTY*
Hconcolorous|010515	**MKLFILVCFMLVLVTLSLA**EQTPCQEKREKILSQNLDVEVIPECEENGSYKAKQCKKNGVDCQCWRTDGTPINDFSPNLKACSCIRSKDNANRPHLIGNYKPQCEADGTYSLTQCWGSVGGCWCVDAEGRKLPNKHFPVDC*
Hconcolorous|010516	**MISKCLRSDKIKMKLFILVCFMLVLVTLSLA**EQTPCQEKREKILSQNLDVEVIPECEENGSYKAKQCKKNGVDCQCWRTDGTPINDFSPNLKACSCIRSKDNANRPHLIGNYKPQCEADGTYSLTQCWGSVGGCWCVDAEGRKLPNKHFPVDC*
Hconcolorous|014939	**MLRLILLCILVATVYS**LSCPCWYEEDTTKYCPPTPTNCPIGLTLGPCGCCLECYKDKGEVCGGSWQMLGKCGEGLRCEKGFDDLGSDYYYANHKEGVCQPIEPIDLL*
Hconcolorous|017887	MSFVIYRFPKLYSYTGVKMRIYVDFSWFQKATHQDMGKKLCLVVFVLLGIFTSAMALTCLACGSYECPPLPLCPAGIVKDVCDCCAVCAKNENENCGGMWEEYGKCGEGLKCVTTGATTPHPFMSDWPIPGICQKE*
Hconcolorous|017888	MSFVIYRFPKLYSYTELKMRIYVDFSWFQKATHQDMGKKLCLVVFVLLGIFTSAMALTCLACGSYECPPLPLCPAGIVKDVCDCCAVCAKNENENCGGMWEEYGKCGEGLKCVTTGATTPHPFMSDWPIPGICQKE*
Hconcolorous|022553	**MWFRFIVLFLFVAGVYS**LSCPCQTNRLLCKPAPTNCKLGLTKDACGCCDVCYKIEGEECGGPWKTSGNCGKGLKCVIPENLPKHIQQQATGICKVE*
Hconcolorous|022554	**MWFRFIVLFLFVAGVYS**LSCPCQRDLSLCEPAPTDCKLGLTKDACGCCDVCYKIEGEECGGPWNTSGNCGKGLKCVIPGNLSKYIELQATGICKVE*
Hconcolorous|028918	**MGVKFYFIAFVLNVVFLNTMA**LKCKECGTYECRPPPNNCPVGTVTDVCNCCLVCGKAENEICGGEWNLLGKCGEGLKCVEKPSSNNQFIAPKAGICKKK*
Hconcolorous|034053	**MRLYLFAFAVLIASCHC**APRNRCTQECGPVPNNCRAGVTKDYDGCCAVCAKSEGEECGGMWNAYGVCGIDLICNTNGNLISDYDLPIGICISARRIVNRNILKRMLRGLH*

Table S3. List of the 24-scorpion species used in the phylogenomic analyses.

	Species	SRA Accession	Reference
**BOTHRIUROIDEA**			
Bothriuridae	*Bothriurus coriaceaus*	SRR6467511	[1]
	Centromachetes sp.	SRR6467879	[1]
	Cercophonius queenslandae	SRR6467879	[1]
	Cercophonius squama	SRR6470146	[1]
	Cercophonius sulcatus	SRR6470446	[1]
**BUTHOIDEA**			
Buthidae	*Androctonus australis*	SRR1724216	[2]
**CHACTOIDEA**			
Chactidae	*Anuroctonus phaiodactylus*	SRR1721879	[2]
Euscorpiidae	*Megacormus gertschi*	SRR3657526	[3]
**HADRUROIDEA**			
Hadruridae	*Hadrurus arizonensis*	SRR1721733	[2]
	Hadrurus spadix	SRR4069278	[4]
	Hadrurus concolorous		This study
	Hoffmannihadrurus gertschi	-	[5]
	Hoffmannihadrurus aztecus		This study
**IUROIDEA**			
Iuridae	*Iurus dekanum*	SRR1721734	[2]
Incertae Sedis			
Troglotayosicidae	*Belisarius xambeui*	SRR1721953	[2]
	Uroctonus mordax	SRR7415024	[6]
**PSEUDOCHACTOIDEA**			
Pseudochactidae	*Vietbocap lao*	SRR1721740	[2]
**SCORPIONOIDEA**			
Scorpionidae	*Pandinus imperator*	SRR1721600	[2]
Urodacidae	*Urodacus elongatus*	SRR7885472	[7]
	Urodacus manicatus	SRR870663	[8]
	Urodacus planimanus	-	[2]
	Urodacus yaschenkoi	SRR1557168	[9]
**VAEJOVOIDEA**			
Vaejovidae	*Paravaejovis schwenkmeyeri*	PRJEB25890	[10]
Vaejovidae	*Serradigitus gertschi*	PRJEB27910	[11]

Table S4. 84 sequences of Scorpine-like Peptides (SLP), plus 23 potassium channel toxins (KTx) isolated from the venom, or deduced from cDNA or transcriptome analyses of 44 scorpion species.

Code	Toxin	Species	Family	Source
A0A059UI30	SLP Buthida	*Mesobuthus gibbosus*	Buthidae	UniProt
A0A088D9S1	SLP Buthida	*Mesobuthus eupeus*	Buthidae	UniProt
A0A088DB53	SLP Buthida	*Mesobuthus eupeus*	Buthidae	UniProt
A0A0C9RFQ9	SLP Buthida	*Tityus bahiensis*	Buthidae	UniProt
A0A0C9S3A8	Beta KTx	*Tityus bahiensis*	Buthidae	UniProt
A0A0K0LBZ4	SLP Buthida	*Androctonus bicolor*	Buthidae	UniProt
A0A0K0LC02	SLP Buthida	*Androctonus bicolor*	Buthidae	UniProt
A0A0K0LC06	SLP Buthida	*Androctonus bicolor*	Buthidae	UniProt
A0A0K0LC11	Beta KTx	*Androctonus bicolor*	Buthidae	UniProt
A0A0K0LC14	SLP2	*Androctonus bicolor*	Buthidae	UniProt
A0A0K0LCI9	SLP Buthida	*Androctonus bicolor*	Buthidae	UniProt
A0A0K0LCJ0	Beta KTx	*Androctonus bicolor*	Buthidae	UniProt
A9XE59	SLP Buthida	*Mesobuthus eupeus*	Buthidae	UniProt
A9XE60	SLP Buthida	*Mesobuthus eupeus*	Buthidae	UniProt
alpha-KTx 12.5 mgc25969	Alpha KTx	*Megacormus gertschi*	Euscorpiidae	From transcriptome
alpha-KTx 23.1 mgc29973	Alpha KTx	*Megacormus gertschi*	Euscorpiidae	From transcriptome
alpha-KTx 6.6 mgc21622	Alpha KTx	*Megacormus gertschi*	Euscorpiidae	From transcriptome
API81325	SLP1	*Hemiscorpius lepturus*	Hemiscorpiidae	UniProt
B.coriaceus m.20282	SLP1	*Bothriurus coriaceus*	Bothriuridae	From transcriptome
B8XH36	Beta KTx	*Buthus occitanus*	Buthidae	UniProt
B8XH40	SLP Buthida	*Buthus occitanus*	Buthidae	UniProt
Be.xambeui m4045	SLP2	*Belisarius xambeui*	Troglotayosicidae	From transcriptome
C.queenslandae m.1064	SLP Bothriuridae	*Cercophonius queenslandae*	Bothriuridae	From transcriptome
C.queenslandae m.7768	SLP1	*Cercophonius queenslandae*	Bothriuridae	From transcriptome
C.squama m4800	SLP Bothriuridae	*Cercophonius squama*	Bothriuridae	From transcriptome
C.squama m5048	SLP1	*Cercophonius squama*	Bothriuridae	From transcriptome
C5J891	SLP2	*Opisthacanthus cayaporum*	Hormuridae	UniProt
Ctri27	SLP Buthida	*Chaerilus tricostatus*	Chaerilidae	[12]
Ctri9164	SLP Buthida	*Chaerilus tricostatus*	Chaerilidae	[12]
Ctry22350	SLP Buthida	*Chaerilus tryznai*	Chaerilidae	[12]
Ctry44	SLP Buthida	*Chaerilus tryznai*	Chaerilidae	[12]
Ctry51	Beta KTx	*Chaerilus tryznai*	Chaerilidae	[12]
D9U2A7	SLP Buthida	*Lychas mucronatus*	Buthidae	UniProt
E4VP14	SLP Buthida	*Mesobuthus eupeus*	Buthidae	UniProt

E4VP56	SLP Buthida	*Mesobuthus eupeus*	Buthidae	UniProt
E4VP57	Beta KTx	*Mesobuthus eupeus*	Buthidae	UniProt
H.concolorous|008061	SLP2	*Hadrurus concolorous*	Hadruridae	From transcriptome
H.concolorous|029426	SLP2	*Hadrurus concolorous*	Hadruridae	From transcriptome
H.concolorous|033550	SLP1	*Hadrurus concolorous*	Hadruridae	From transcriptome
H.spadix DN50359	SLP2	*Hadrurus spadix*	Hadruridae	From transcriptome
H2CYP8	SLP1	*Pandinoides cavimanus*	Scorpionidae	UniProt
H2CYQ1	Beta KTx	*Pandinoides cavimanus*	Scorpionidae	UniProt
Hg scorpine like 2 mgc19218	SLP2	*Megacormus gertschi*	Euscorpiidae	From transcriptome
Hge-beta-KTx mgc25471	Beta KTx	*Megacormus gertschi*	Euscorpiidae	From transcriptome
Ho.aztecus|062933	SLP2	*Hoffmannihadrurus aztecus*	Hadruridae	From transcriptome
Ho.aztecus|083866	SLP1	*Hoffmannihadrurus aztecus*	Hadruridae	From transcriptome
Ho.aztecus|106327	SLP2	*Hoffmannihadrurus aztecus*	Hadruridae	From transcriptome
Ho.gertschi EL698900.1.p1	SLP2	*Hoffmannihadrurus gertschi*	Hadruridae	From transcriptome
Ho.gertschi EL698908.1.p1	SLP1	*Hoffmannihadrurus gertschi*	Hadruridae	From transcriptome
K9LZ65	Beta KTx	*Tityus stigmurus*	Buthidae	UniProt
L0G8Z0	SLP1	*Urodacus yaschenkoi*	Urodacidae	UniProt
L0GCW2	SLP2	*Urodacus yaschenkoi*	Urodacidae	UniProt
P.schwenkmeyeri c22486	SLP2	*Paravaejovis schwenkmeyeri*	Vaejovidae	From transcriptome
P.schwenkmeyeri c22791	SLP1	*Paravaejovis schwenkmeyeri*	Vaejovidae	From transcriptome
P.schwenkmeyeri c22885	SLP2	*Paravaejovis schwenkmeyeri*	Vaejovidae	From transcriptome
P0C2F3	SLP Buthida	*Tityus stigmurus*	Buthidae	UniProt
P0C2F4	SLP1	*Heterometrus laoticus*	Scorpionidae	UniProt
P0C8W4	Beta KTx	*Tityus stigmurus*	Buthidae	UniProt
P0C8W5	SLP2	*Hoffmannihadrurus gertschi*	Hadruridae	UniProt
P0CI49	SLP Buthida	*Lychas mucronatus*	Buthidae	UniProt
P0DL47	SLP1	*Euscorpiops validus*	Scorpiopidae	UniProt
P56972	SLP1	*Pandinus imperator*	Scorpionidae	UniProt
P69939	SLP Buthida	*Androctonus australis*	Buthidae	UniProt
P69940	SLP Buthida	*Tityus serrulatus*	Buthidae	UniProt
P86822	Beta KTx	*Tityus serrulatus*	Buthidae	UniProt
Q0GY40	SLP1	*Hoffmannihadrurus gertschi*	Hadruridae	UniProt
Q0GY41	Beta KTx	*Hoffmannihadrurus gertschi*	Hadruridae	UniProt
Q0GY42	Beta KTx	*Tityus costatus*	Buthidae	UniProt
Q0GY43	Beta KTx	*Tityus discrepans*	Buthidae	UniProt
Q0GY44	SLP Buthida	*Tityus discrepans*	Buthidae	UniProt
Q0GY45	Beta KTx	*Tityus trivittatus*	Buthidae	UniProt
Q0GY46	SLP Buthida	*Tityus trivittatus*	Buthidae	UniProt

Q5G8A6	SLP Buthida	*Tityus costatus*	Buthidae	UniProt
Q5WQZ7	SLP1	*Opistophthalmus carinatus*	Scorpionidae	UniProt
Q5WQZ9	SLP1	*Opistophthalmus carinatus*	Scorpionidae	UniProt
Q5WR01	SLP1	*Opistophthalmus carinatus*	Scorpionidae	UniProt
Q5WR03	SLP1	*Opistophthalmus carinatus*	Scorpionidae	UniProt
Q6XLL8	Alpha KTx	*Opisthacanthus cayaporum*	Hormuridae	UniProt
Q9N661	SLP Buthida	*Mesobuthus martensii*	Buthidae	UniProt
Q9NJC6	Beta KTx	*Mesobuthus martensii*	Buthidae	UniProt
S.gertschi c25700	SLP2	*Serradigitus gertschi*	Vaejovidae	From transcriptome
S.gertschi c26014	SLP1	*Serradigitus gertschi*	Vaejovidae	From transcriptome
S.gertschi c26395	SLP2	*Serradigitus gertschi*	Vaejovidae	From transcriptome
Scorpine-like peptide Ev37 mgc22639	SLP1	*Megacormus gertschi*	Euscorpiidae	From transcriptome
Su.donensis sdc1422	SLP2	*Superstitionia donensis*	Superstitioniidae	From transcriptome
Su.donensis sdc34997	SLP1	*Superstitionia donensis*	Superstitioniidae	From transcriptome
T1DEJ8	SLP2	*Urodacus manicatus*	Urodacidae	UniProt
T1DEK6	SLP Buthida	*Isometroides vescus*	Buthidae	UniProt
T1DMR0	SLP1	*Cercophonius squama*	Botrhiuridae	UniProt
T1DMR6	Beta KTx	*Urodacus manicatus*	Urodacidae	UniProt
T1DP99	SLP Bothriuridae	*Cercophonius squama*	Botrhiuridae	UniProt
T1E6W5	SLP Buthida	*Australobuthus xerolimniorum*	Buthidae	UniProt
T1E6W8	SLP Bothriuridae	*Cercophonius squama*	Botrhiuridae	UniProt
T1E6X2	SLP1	*Urodacus manicatus*	Urodacidae	UniProt
T1E6Y1	Beta KTx	*Isometroides vescus*	Buthidae	UniProt
Toxin KTx8 mgc26539	Alpha KTx	*Megacormus gertschi*	Euscorpiidae	From transcriptome
Ur.mordax DN49907	SLP2	*Uroctonus mordax*	Hadruridae	From transcriptome
V.lao m10170	SLP2	*Vietbocap lao*	Pseudochactidae	From transcriptome
ViScp1p1	SLP1	*Thorellius intrepidus*	Vaejovidae	[13]
ViScp1p2	SLP1	*Thorellius intrepidus*	Vaejovidae	[13]
ViScp1p3	SLP2	*Thorellius intrepidus*	Vaejovidae	[13]
VmScp1p1	SLP1	*Vaejovis mexicanus*	Vaejovidae	[13]
VmScp1p2	SLP2	*Vaejovis mexicanus*	Vaejovidae	[13]
VmScp1p3	SLP2	*Vaejovis mexicanus*	Vaejovidae	[13]
VpScp1p1	SLP1	*Mesomexovis punctatus*	Vaejovidae	[13]
VsScp1p1	SLP1	*Mesomexovis subcristatus*	Vaejovidae	[13]
VsScp1p2	SLP2	*Mesomexovis subcristatus*	Vaejovidae	[13]

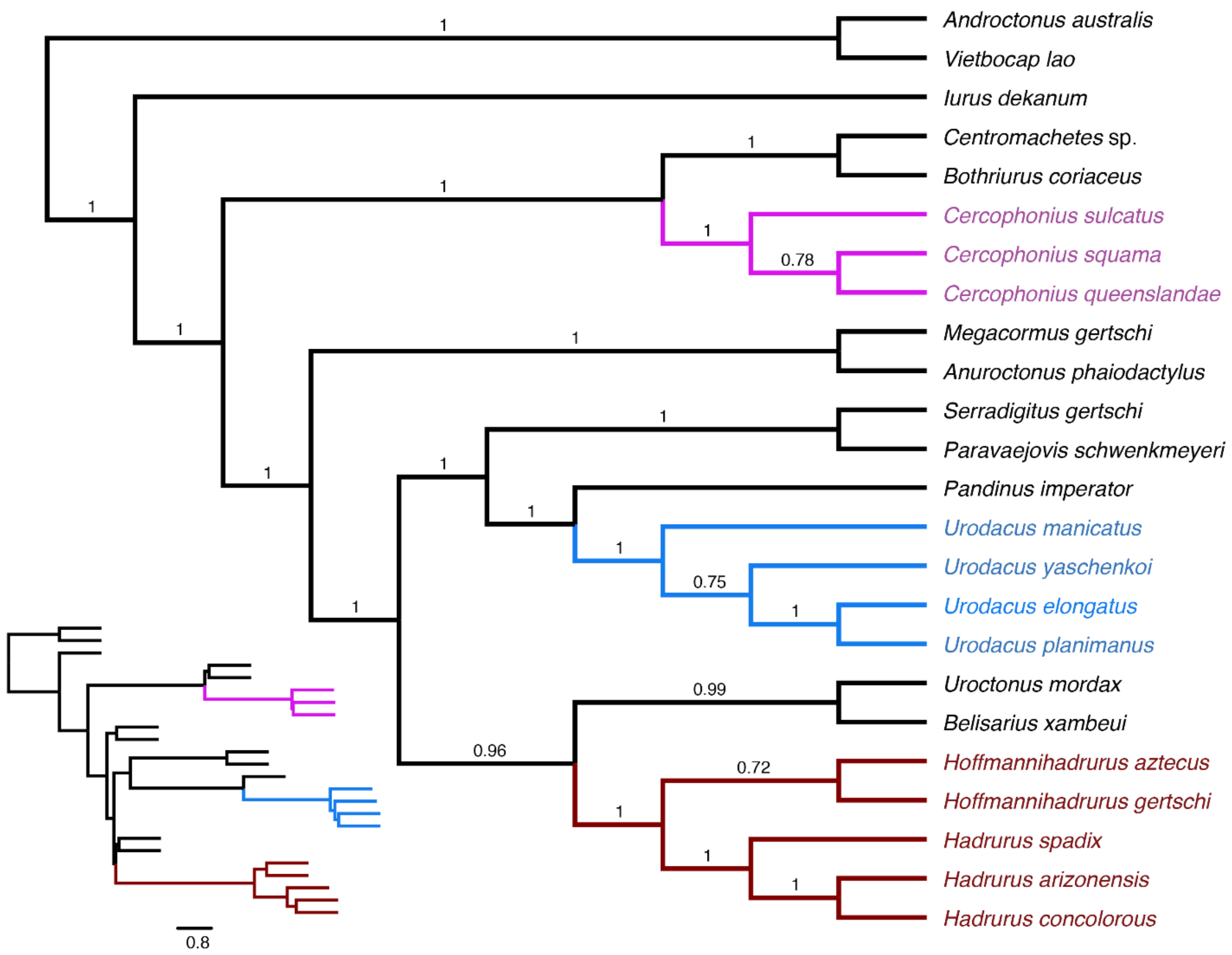

Figure S1. ASTRAL-II tree recovered from the 1,982 orthobranches with at least 12 species per orthogroup. Number above branches indicate branch support from local posterior probabilities.

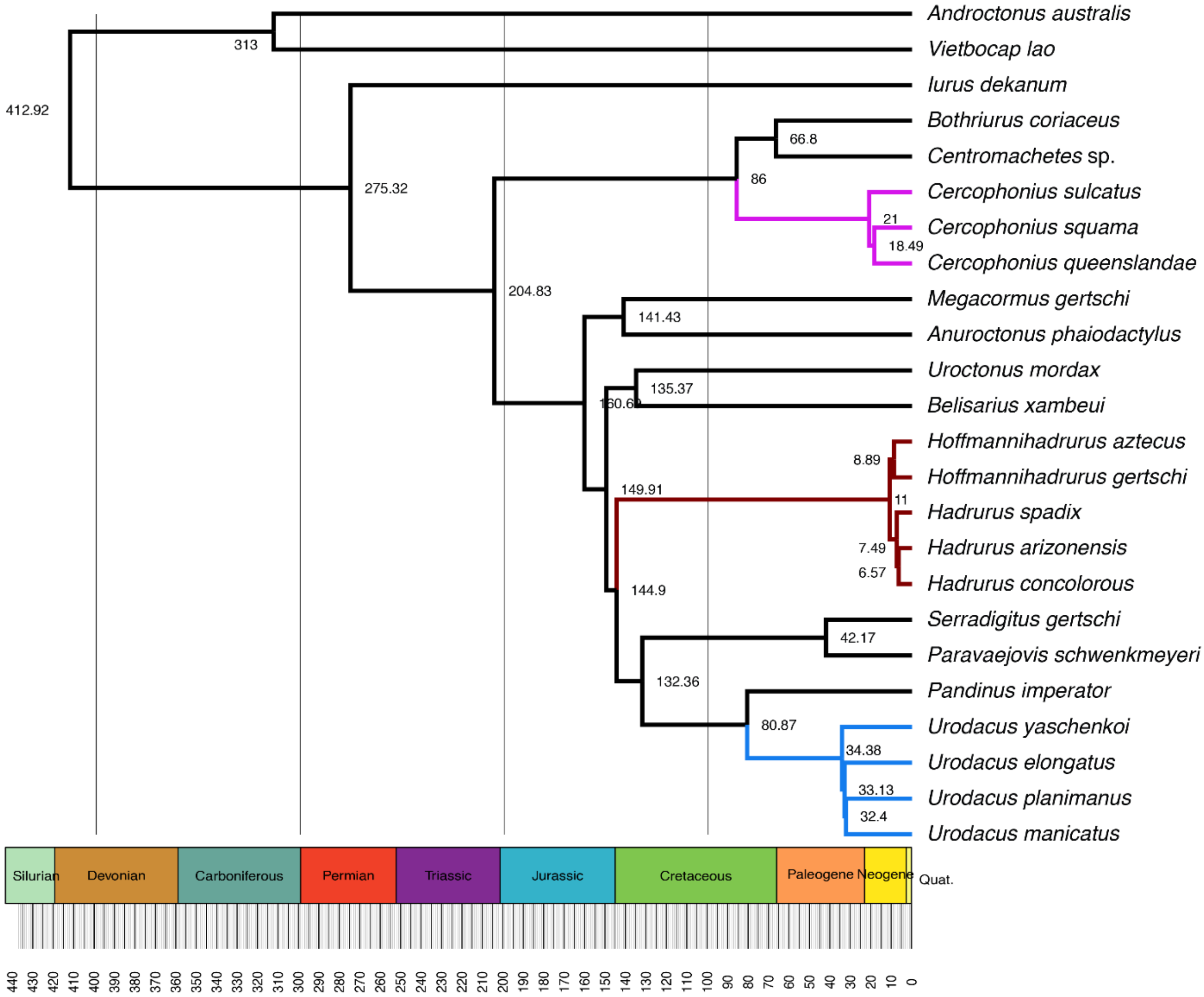

Figure S2. Chronogram of the divergence times in our ML tree topology calibrated using the penalized likelihood, as implemented in the *chronos* function under the correlated rates clock model and lambda = 1.

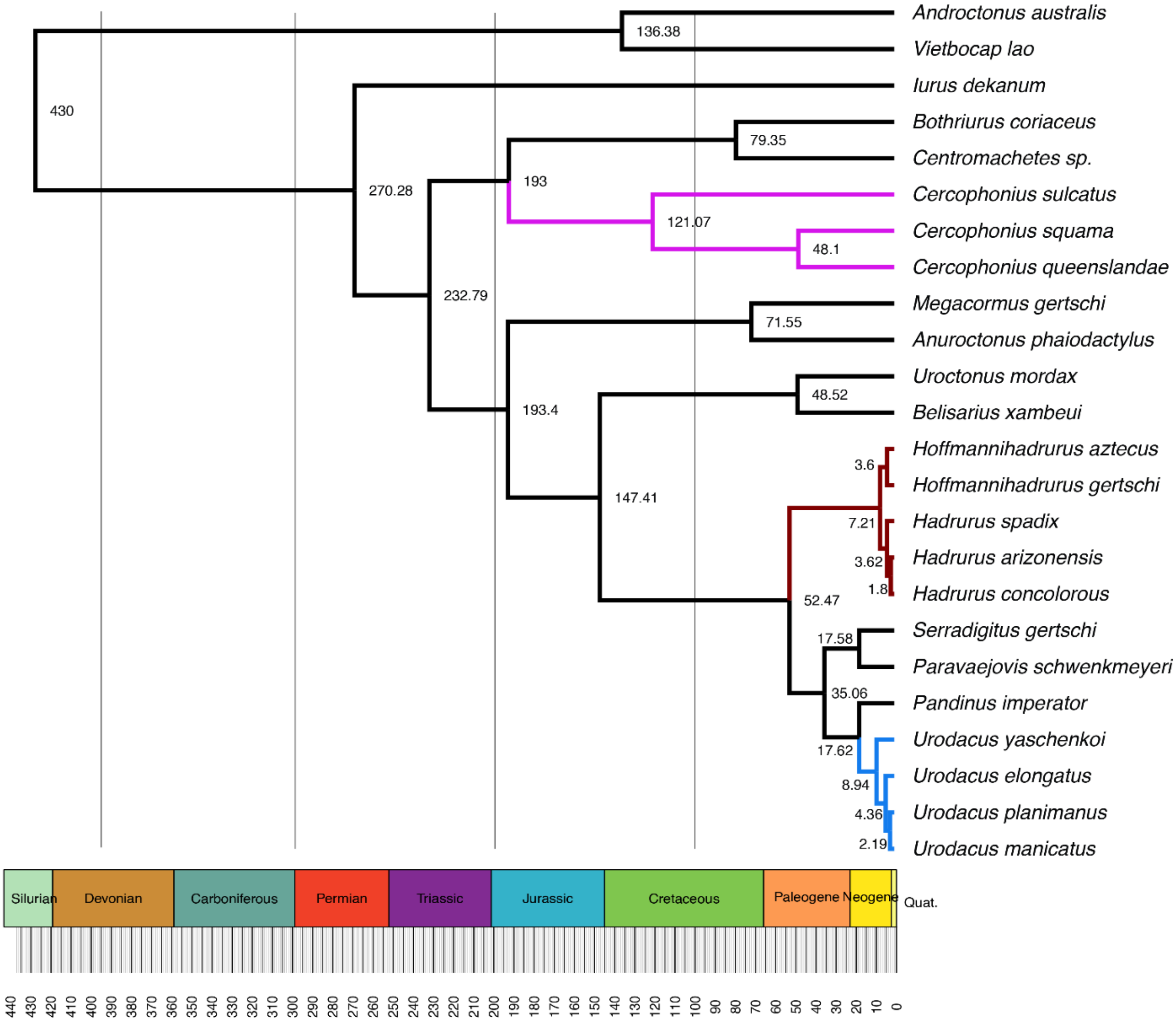

Figure S3. Chronogram of the divergence times in the ML tree topology calibrated using the penalized likelihood, as implemented in the *chronos* function under the relaxed clock model and lambda = 1.

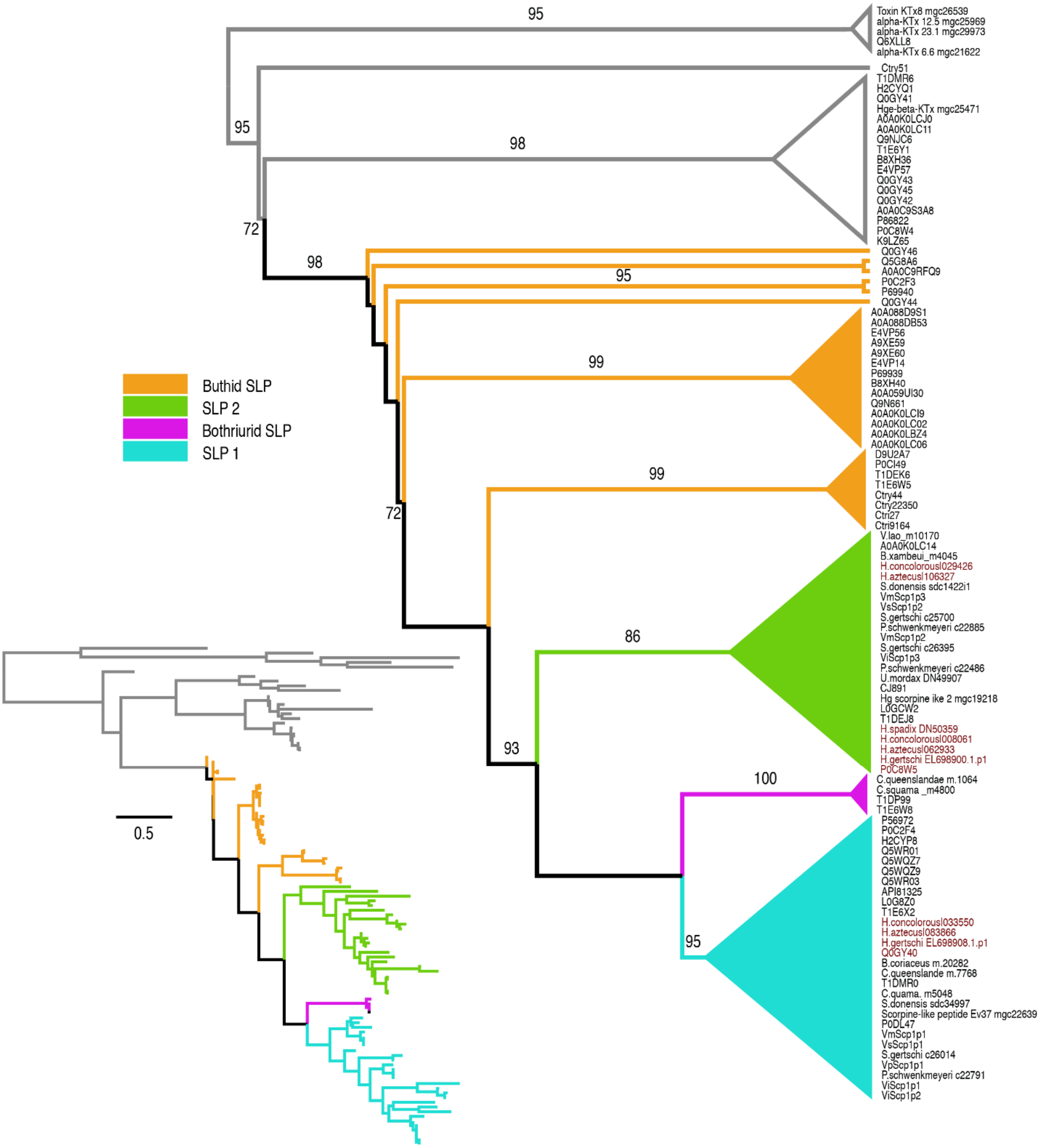

Figure S4. Maximum Likelihood gene tree obtained from the analysis of 107 sequences of scorpion toxins (SLP and allies). Number above branches indicate ultrafast bootstrap support values.

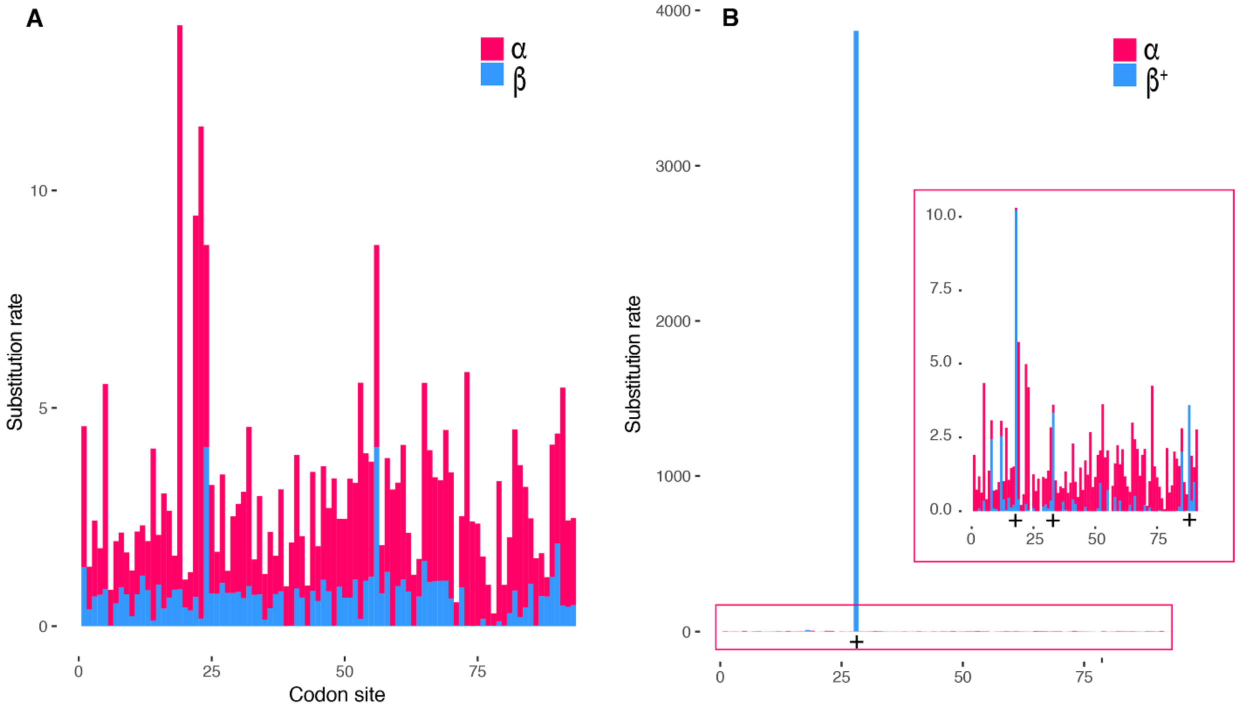

Figure S5. Site selection analyses of both SLP1-2 combined with FUBAR (**A**) and MEME (**B**). Visualization of the difference between the values of α in red and β (FUBAR; β+ in MEME) in blue. Inset: enlarged area of the visualization of the MEME analysis. + signs indicate β+ values were greater than α with a $p > 0.95$.

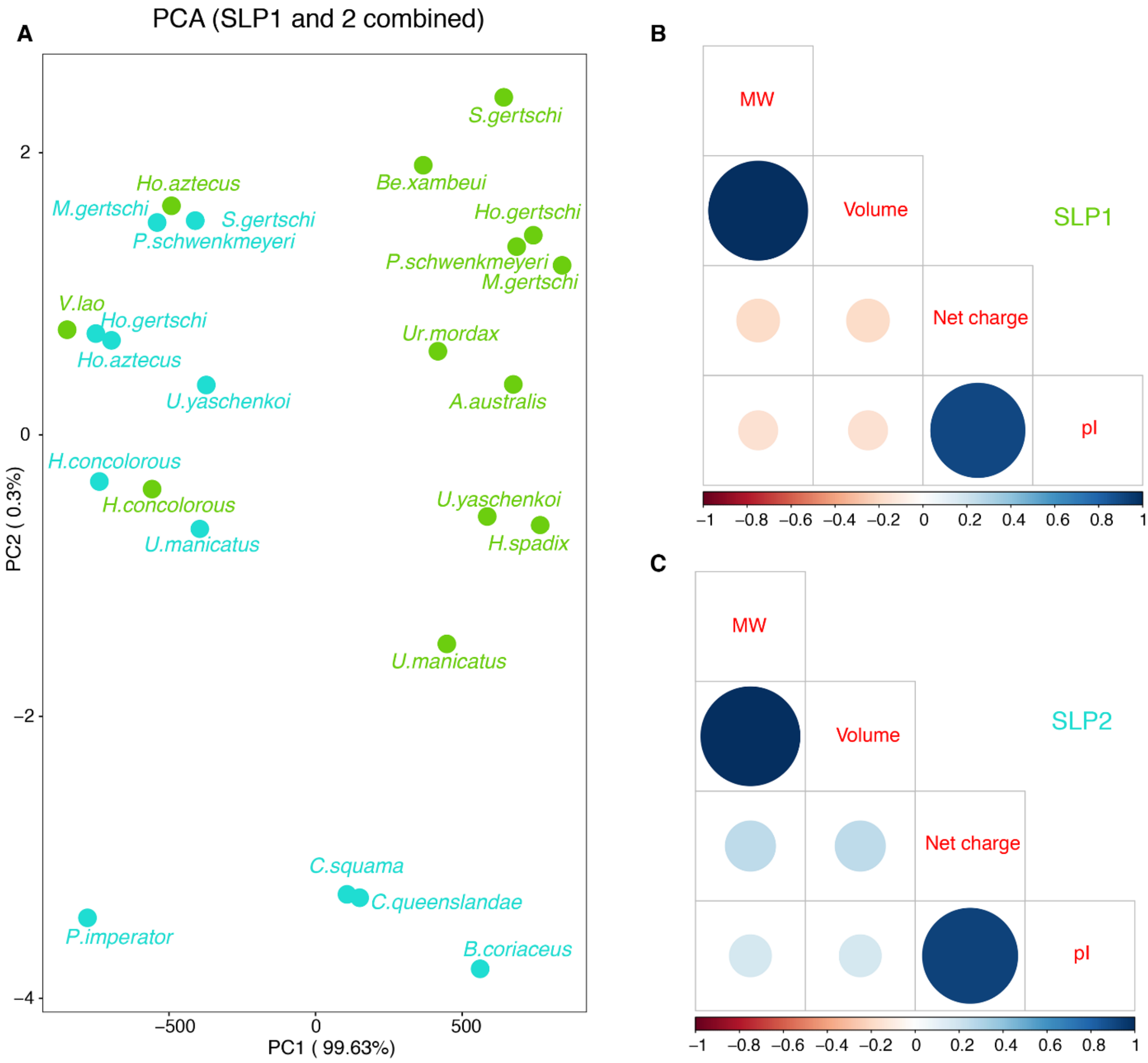

Figure S6. (**A**) Visualization of the variation of the four biochemical properties of SLP1-2 in the first two principal components. (**B**-**C**). Kendall rank correlation test between the four biochemical properties from SLP1 (B) and SLP2 (**C**). MW = Molecular weight, Volume = Molecular volume, Net charge and pI = Isoelectric point. Color of the circle indicates positive or negative correlation coefficient, increasing size of the circle indicates smaller *p*-value.

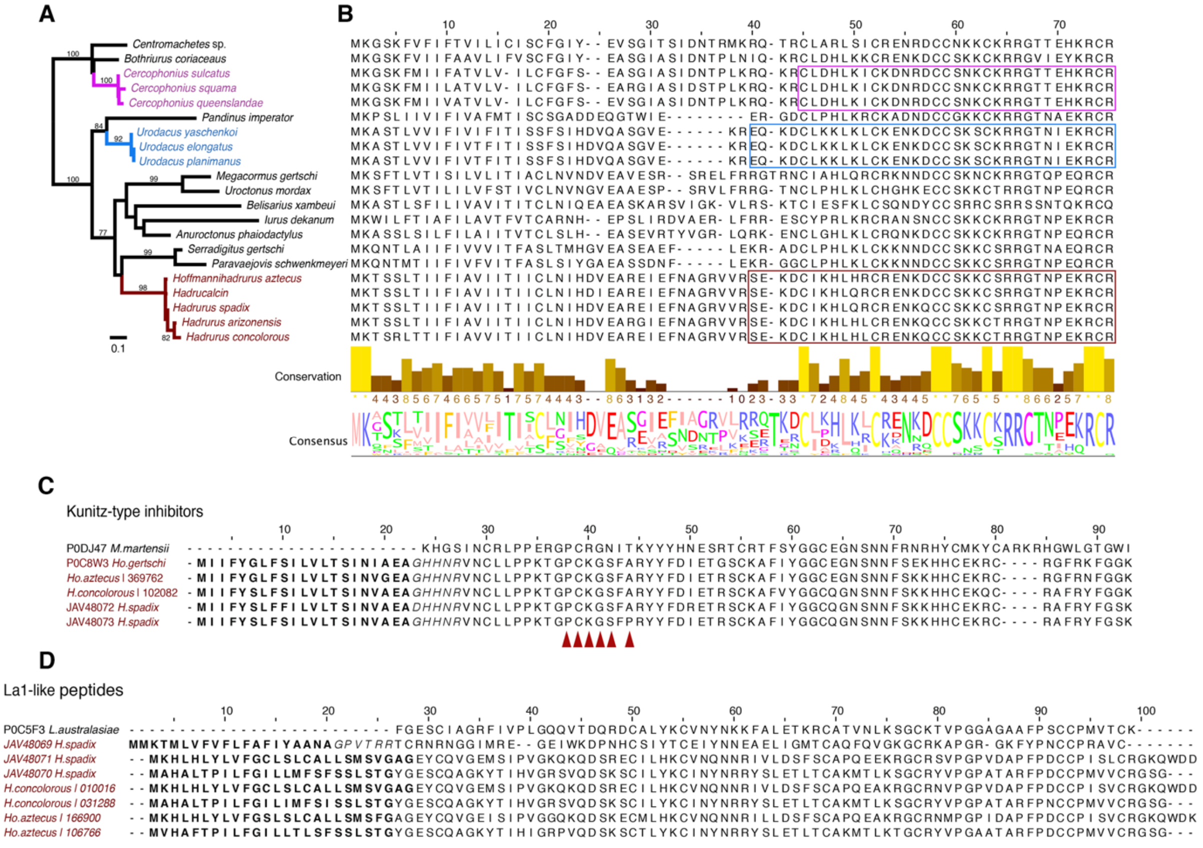

Figure S7. (A) Evolutionary tree of calcins (ICK peptides) from ML analysis of 21 sequences reported from iurid scorpions. Ultrafast boostrap values reported above node (>75%). (**B**) Multiple sequence alignment of the calcin full precursor used in the phylogenetic analysis. The mature peptide of hadrurids, urodacids and cercophonids are highlighted in squares colored by genera. (**C**) Multiple sequence alignment of the Kunitz-type inhibitors deduced from the cDNA of hadrurid species and one buthid. Signal peptide is highlighted in bold, propeptide in light italics. Trypsin interaction sites indicate by red triangles. (**D**) Multiple sequence alignment of the La1-like peptides. Signal peptide is highlighted in bold, propeptide in light italics.

References

1. Sharma, P.P.; Baker, C.M.; Cosgrove, J.G.; Johnson, J.E.; Oberski, J.T.; Raven, R.J.; Harvey, M.S.; Boyer, S.L.; Giribet, G. A revised dated phylogeny of scorpions—Phylogenomic support for ancient divergence of the temperate Gondwanan family Bothriuridae. *Mol. Phylogen. Evol.* **2018**, *122*, 37–45.
2. Sharma, P.P.; Fernández, R.; Esposito, L.A.; González-Santillán, E.; Monod, L. Phylogenomic resolution of scorpions reveals multilevel discordance with morphological phylogenetic signal. *Proc. Biol. Sci.* **2015**, *282*, 20142953.
3. Santibáñez-López, C.E.; Cid-Uribe, J.I.; Zamudio, F.Z.; Batista, C.V.F.; Ortiz, E.; Possani, L.D. Venom gland transcriptomic and venom proteomic analyses of the scorpion *Megacormus gertschi* Díaz-Najera, 1966 (Scorpiones: Euscorpiidae: Megacorminae). *Toxicon* **2017**, *133*, 95–109.
4. Rokyta, D.R.; Ward, M.J. Venom-gland transcriptomics and venom proteomics of the black- back scorpion (*Hadrurus spadix*) reveal detectability challenges and an unexplored realm of animal toxin diversity. *Toxicon* **2017**, *128*, 23–37.
5. Schwartz, E.F.; Diego-Garcia, E.; Rodríguez de la Vega, R.C.; Possani, L.D. Transcriptome analysis of the venom gland of the Mexican scorpion *Hadrurus gertschi* (Arachnida: Scorpiones). *BMC Genom.* **2007**, *8*, 119.
6. Santibáñez-López, C.E.; González-Santillán, E.; Monod, L.; Sharma, P.P. Phylogenomics facilitates stable scorpion systematics_ Reassessing the relationships of Vaejovidae and a new higher-level classification of Scorpiones (Arachnida). *Mol. Phylogen. Evol.* **2019**, *135*, 22–30.

7. Santibáñez-López, C.E.; Kriebel, R.; Ballesteros, J.A.; Rush, N.; Witter, Z.; Williams, J.; Janies, D.A.; Sharma, P.P. Integration of phylogenomics and molecular modeling reveals lineage-specific diversification of toxins in scorpions. *PeerJ* **2018**, *6*, e5902.
8. Sunagar, K.; Undheim, E.; Chan, A.; Koludarov, I.; Muñoz-Gómez, S.; Antunes, A.; Fry, B. Evolution Stings: The Origin and Diversification of Scorpion Toxin Peptide Scaffolds. *Toxins* **2013**, *5*, 2456–2487.
9. Luna-Ramírez, K.; Quintero-Hernández, V.; Juárez-González, V.R.; Possani, L.D. Whole Transcriptome of the Venom Gland from *Urodacus yaschenkoi* Scorpion. *PLoS ONE* **2015**, *10*, e0127883.
10. Cid-Uribe JI, Santibáñez-López CE, Meneses EP, Batista CVF, Jiménez-Vargas JM, Ortiz, E., Possani LD. The diversity of venom components of the scorpion species *Paravaejovis schwenkmeyeri* (Scorpiones: Vaejovidae) revealed by transcriptome and proteome analyses. *Toxicon* **2018**, *151*, 47–62.
11. Romero-Gutiérrez, M.; Santibáñez-López, C.; Jiménez-Vargas, J.; Batista, C.; Ortiz, E.; Possani, L. Transcriptomic and Proteomic Analyses Reveal the Diversity of Venom Components from the Vaejovid Scorpion *Serradigitus gertschi*. *Toxins* **2018**, *10*, 359.
12. He, Y.; Zhao, R.; Di, Z.; Li, Z.; Xu, X.; Hong, W.; Wu, Y.; Zhao, H.; Li, W.; Cao, Z. Molecular diversity of Chaerilidae venom peptides reveals the dynamic evolution of scorpion venom components from Buthidae to non-Buthidae. *J. Proteom*. **2013**, *89*, 1–14.
13. Quintero-Hernández, V.; Ramírez-Carreto, S.; Romero-Gutiérrez, M.T.; Valdez-Velázquez, L.L.; Becerril, B.; Possani, L.D.; Ortiz, E. Transcriptome analysis of scorpion species belonging to the *Vaejovis* genus. *PLoS ONE* **2015**, *10*, e0117188.
